# Increasing cancer risk over calendar year in people with multiple sclerosis: a case–control study

**DOI:** 10.1007/s00415-020-10170-5

**Published:** 2020-10-21

**Authors:** Chiara Zecca, Giulio Disanto, Rosaria Sacco, Sharon MacLachlan, Jens Kuhle, Sreeram V. Ramagopalan, Claudio Gobbi

**Affiliations:** 1 Neurocenter of Southern Switzerland, Ospedale Regionale di Lugano Civico e Italiano, Via Tesserete 46, 6903 Lugano, Switzerland; 2 Faculty of Biomedical Sciences, Università della Svizzera Italiana, Via Buffi 13, 6900 Lugano, Switzerland; 3 Evidera, The Ark, 201 Talgarth Rd, London, W6 8BJ UK; 4 Departments of Medicine, Biomedicine and Clinical Research, Neurologic Clinic and Policlinic, University Hospital Basel, University of Basel, Petersgraben 4, 4031 Basel, Switzerland; 5 Bristol-Myers Squibb, Sanderson Rd, Denham, Uxbridge, UB8 1DH UK

**Keywords:** Cancer, Demyelinating autoimmune diseases, CNS, Multiple sclerosis, Neoplasms, Risk

## Abstract

**Background:**

Data on cancer prevalence and incidence in multiple sclerosis (MS) patients are controversial. This study is aimed at estimating cancer risk in MS patients.

**Methods:**

Nested case–control study using data collected between 01/01/1987 and 28/02/2016 from the United Kingdom Clinical Practice Research Datalink. Cancer diagnoses after first MS code (index date) was counted in 10,204 MS patients and 39,448 controls matched by sex, age, general practitioner, and registration year. Cancer rates were compared using multivariable Cox regression models. Ethics approval was not required.

**Results:**

Cancer was reported in 433 (4.41%) MS patients and 2014 (5.31%) controls after index date. Cancer risk was associated with gender (HR for female = 0.88, 95% CI = 0.81–0.96, *p* = 0.004), age at index date (HR = 1.06, 95% CI = 1.06–1.07, *p* < 0.001), and index year (HR = 1.01, 95% CI = 1.00–1.02, *p* = 0.016), but not with MS status (HR = 0.95, 95% CI = 0.86–1.05, *p* = 0.323). A significant interaction between MS status and index year was found (HR = 1.02, 95% CI = 1.00–1.04, *p* = 0.022). Cancer risk was positively associated with index year among MS patients (HR = 1.03, 95% CI = 1.01–1.05; *p* = 0.010), but not controls (HR = 1.01, 95% CI = 0.99–1.02; *p* = 0.144). MS patients compared to controls had no increased risk for any specific cancer type.

**Conclusions:**

Overall cancer risk was similar in multiple sclerosis patients and matched controls. The frequency of cancer diagnoses has increased over time among MS patients but not in controls.

**Electronic supplementary material:**

The online version of this article (10.1007/s00415-020-10170-5) contains supplementary material, which is available to authorized users.

## Introduction

The interplay between cancer and immune-mediated diseases as multiple sclerosis (MS) is intriguing and has been debated for several years. It has been suggested that the abnormal immune response seen in MS could improve surveillance against malignancy [1]. However, chronic inflammation also represents a recognized risk factors for cancer development [2].

Studies assessing the prevalence and incidence of cancer in MS reflect this controversy, inconsistently showing similar [3–6], reduced [7–10], or increased risk [11, 12] as compared to the general population.

Different study designs, methods of case ascertainment and study periods may well explain at least part of such conflicting findings [13]. Genetics, as well as established MS-associated environmental factors such as smoking, obesity, physical activity, and socioeconomic status may also modulate risk of cancer in patients with MS [7, 14], acting as relevant confounders when not accounted for. Additionally, the continuous evolution of MS immunomodulatory and immunosuppressive agents possibly influences immune surveillance and cancer development in MS. Finally, only few studies have included MS patients diagnosed with MS during the last decade and no clear temporal trends in cancer diagnoses among MS patients has emerged [13–17].

We, therefore, aimed at investigating in primary care settings the occurrence of cancer in MS patients as compared to matched controls from the general population, and how this has evolved over time in the last 25 years, using the United Kingdom (UK) Clinical Practice Research Datalink (CPRD) [18]. As an exploratory aim, we also estimated cancer occurrence in MS patients and paired controls before a diagnosis of MS is made.

## Methods

### Study population

We conducted a population-based nested case–control study using data from the validated UK’s CPRD [19, 20] as described in Disanto et al. [21]. This governmental research service prospectively collects electronically routine primary care data since 1987 (https://www.cprd.com/home/) [18]. These include demographic and clinical information such as diagnoses, symptoms, medications, and tests. Validation studies have been performed supporting the reliability and quality of CPRD data and CPRD-coded diagnoses, including MS [19, 20]. Such data were provided to our group upon request and approval by the Independent Scientific Advisory Committee of the CPRD [21].

### Study design and selection of cases and controls

**Fig. 1:**
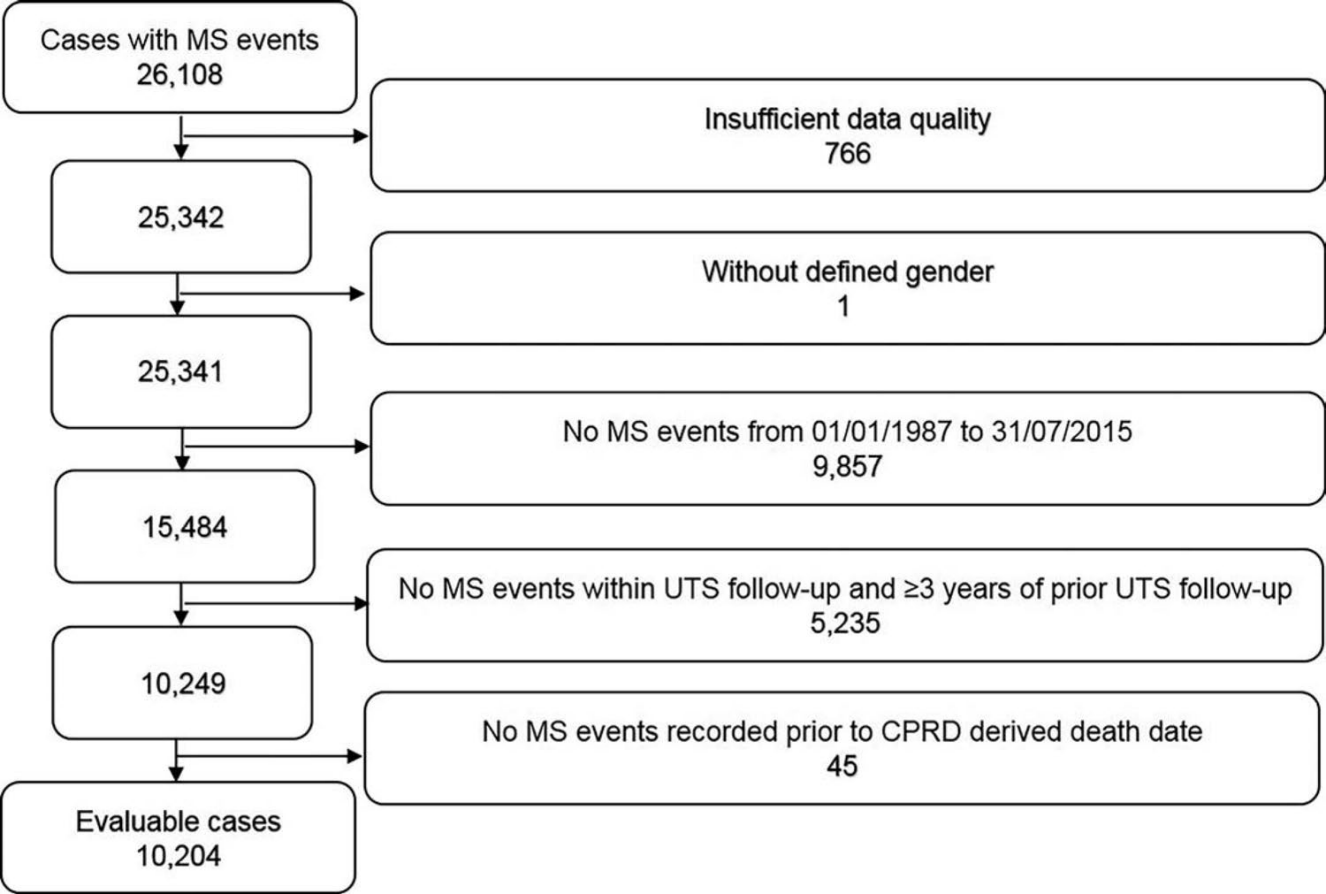
Selection of evaluable Cases with multiple sclerosis events. *CPRD* Clinical Practice Research Datalink, *UTS* up-to-standard

This was a nested case–control study that used data collected from the CPRD to compare the occurrence of cancer between cases [i.e. individuals who received a diagnosis of MS or clinically isolated syndrome (CIS)] and controls (i.e. individuals with no MS or CIS record). Cases and controls were recorded in CPRD GOLD at March 2016. Inclusion criteria for cases were: (1) a clinical or referral MS event record with a specified read code indicating a diagnosis of MS or CIS at any time in the clinical or referral files; (2) validity of the records in terms of continuous follow-up and data recording (as defined by CPRD standard criteria); (3) a defined gender (male and female only); (4) at least one MS event occurring within the study period (01/01/1987–28/02/2016); (5) MS events occurring within the up-to-standard (UTS) follow-up period and after at least 3 years of prior UTS follow-up. The UTS is defined as the date from which the practice fulfils high-quality data criteria based on continuity and death recording; (6) MS events recorded before the death date derived from CPRD (Fig. 1). Each case was matched to up to four controls with no record of MS by sex, year of birth (5-year bands), general practitioner practice, and year of registration. The index date was defined as the date of the first MS code reported in the dataset for cases, and a matched index date for controls.

### Diagnoses of interest

More than 100,000 single unique read codes with a related medical term are available in the CPRD. This list was systematically reviewed for read codes indicating diagnoses of cancer, and grouping similar pathological entities according to the following list: any cancer, brain, eye, ear–nose–throat, Hodgkin lymphoma, non-Hodgkin lymphomas, leukemias, lung, breast, hepatic, gastrointestinal, prostate, urinary tract, genital (male and female), connective tissue, non-melanoma skin, and melanoma skin cancer. All read codes used in the analysis are listed in Supplementary Table 1.

**Table 1:** Demographic characteristics and length of up-to-standard follow-up before and after index date

Demographics, follow-up		All individuals (*n* = 49,652)	
		MS patients (*n* = 10,204)	Controls (*n* = 39,448)
Male	*n* (%)	2896 (28.4)	11,200 (28.4)
Female	*n* (%)	7308 (71.6)	28,248 (71.6)
Age at index date (years)	median (IQR)	47 (39–57)	47 (39–56)
UTS follow-up after index date (years)	median (IQR)	5.6 (2.4–9.9)	6.2 (2.7–10.6)
UTS follow-up before index date (years)	median (IQR)	5.9 (4.0–9.5)	5.8 (4.0–9.5)

*IQR* interquartile range, *UTS* up-to-standard

### Statistical analyses

Categorical and continuous variables were described using median with interquartile range (IQR) and counts with percentages, as appropriate. We first estimated the proportion of cases and controls with a record indicating the occurrence of any and each category of cancer from index date until last observation or death. For this analysis, all individuals (both MS patients and controls) with a cancer code of interest appearing before index date were excluded. All analyses for gender-specific cancers (e.g. breast and prostate cancer for females and males), were performed considering the subgroups of patients and controls with the gender of interest. Survival analyses were used to compare the occurrence of a cancer diagnosis between MS patients and controls over time, with index date as the baseline. The end of the follow-up was defined as the date of death, practice last collection date or the date the patient transferred out. Hazard ratios (HR) with 95% confidence intervals (CI) were estimated using multivariable Cox regression models, adjusted for gender, age at index date and index calendar year. As an exploratory analysis, we also estimated the proportion of cases and controls with a record of any cancer within 0–2, 2–5 and 5–10 years before index date. Case/control rates were then compared and odds ratios (OR) with 95% CI generated using multivariable logistic regression, with MS status as the predicted and cancer occurrence as the predicting variable, adjusted by age and gender.

## Results

### Demographic characteristics of cases and controls

A total of 10,204 MS patients fulfilled the inclusion criteria (Fig. 1) and were matched with 39,448 controls. More than 99% (*n* = 10,117) of MS patients had at least one, and 94% (*N* = 9,585) had at least four matched controls. Females were 71.6% in both groups, while the median (IQR) age at index date was 47 (39–57) in MS patients and 47 (39–56) years in controls. The median (IQR) time between start of UTS follow-up and index date was 5.9 (4.0–9.5) in MS patients and 5.8 (4.0–9.5) years in controls. The median (IQR) follow-up after index date was 5.6 (2.4–9.9) and 6.2 (2.7–10.6) years in MS patients and controls, respectively (Table 1).

### Risk of cancer after index date in MS patients and controls

#### Risk of any cancer

A total of 388 MS patients and 1547 controls received a cancer-related code before index date and were, therefore, excluded, leaving 9816 MS patients and 37,901 controls available for analysis. Out of these, 433 (4.41%) MS patients and 2014 (5.31%) controls received a read code related to any cancer between index date and last available follow-up (Table 2).

**Table 2:** Occurrence of any cancer among MS patients and matched controls after index date, overall and stratified by 5 year bands

Epoch after index date	MS patients		Matched controls	
	All cases	Cancer cases	All cases	Cancer cases
	*n*	*n* (%)	*n*	*n* (%)
1991–2016	9816	433 (4.41)	37,901	2014 (5.31)
1991–1995	809	53 (6.55)	3114	369 (11.85)
1996–2000	1352	99 (7.32)	5218	429 (8.22)
2001–2005	2705	140 (5.17)	10,473	709 (6.77)
2006–2010	2787	112 (4.02)	10,713	379 (3.54)
2011–2016	2163	29 (1.34)	8383	128 (1.53)

**Fig. 2:**
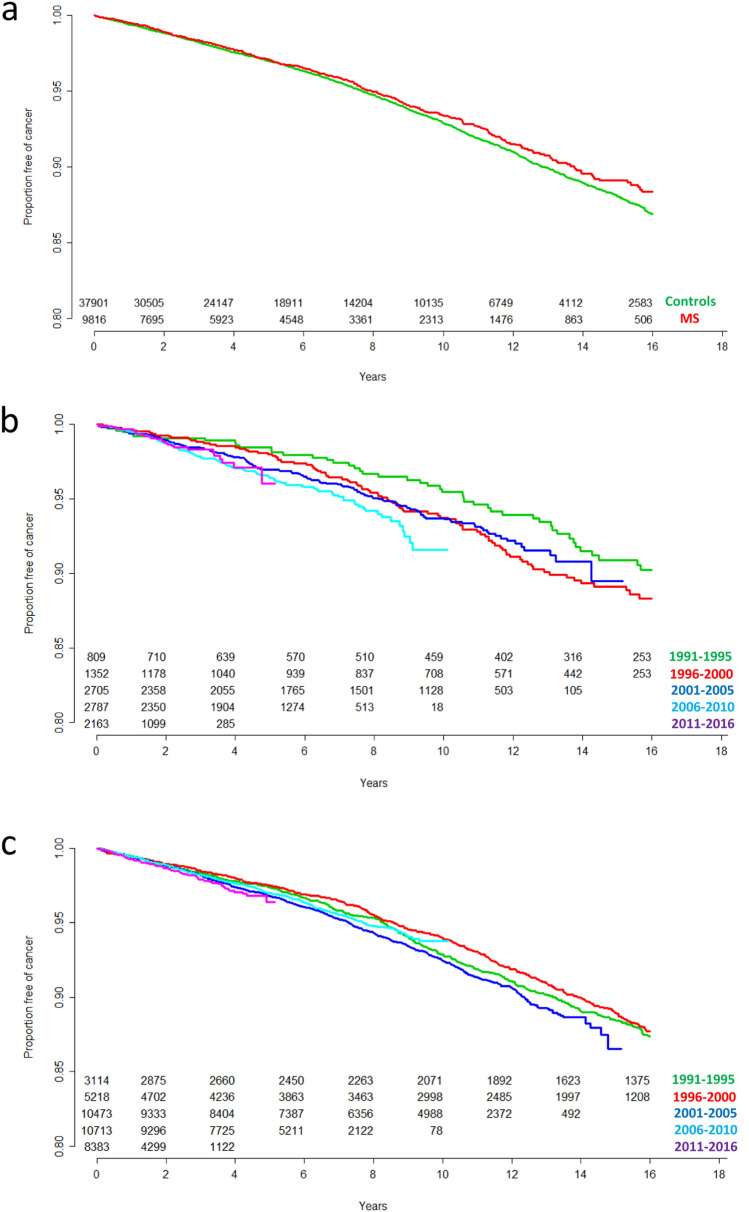
**a** Proportion of MS patients (red) and controls (green) free of any cancer diagnosis across time; **b** Proportion of MS patients free of any cancer diagnosis across time stratified by calendar year at index date in 5 year bands; **c** Proportion of matched controls free of any cancer diagnosis across time stratified by calendar year at index date in 5 year bands. *MS* multiple sclerosis

When using a multivariable Cox regression model, the risk of any cancer after index date was negatively associated with female gender (HR = 0.88, 95% CI = 0.81–0.96, *p* = 0.004), while a positive association was present with age at index date (HR = 1.06, 95% CI = 1.06–1.07, *p* < 0.001) and calendar year at index date (HR = 1.01, 95% CI = 1.00–1.02, *p* = 0.016). Notably, disease status (MS vs control) was not associated with risk of cancer (HR = 0.95, 95% CI 0.86–1.05, *p* = 0.323) (Table 3, Fig. 2a).

**Table 3:** Multivariable Cox regression models testing variables associated with frequency of any cancer after index date among the overall population, only MS patients and only controls

Population	Predicting variable	HR	95% CI	*p*
MS patients and controls	Disease status			
	MS	0.95	0.86–1.05	0.323
	Control	–	–	–
	Gender			
	Female	0.88	0.81–0.96	0.004
	Male	–	–	–
	Age at index date	1.06	1.06–1.07	< 0.001
	Calendar year at index date	1.01	1.00–1.02	0.016
MS patients	Gender			
	Female	0.86	0.70–1.06	0.153
	Male	–	–	–
	Age at index date	1.06	1.05–1.07	< 0.001
	Calendar year at index date	1.03	1.01–1.05	0.010
Controls	Gender			
	Female	0.89	0.81–0.97	0.012
	Male	–	–	–
	Age at index date	1.06	1.06–1.07	< 0.001
	Calendar year at index date	1.01	0.99–1.02	0.144

*HR* hazard ratio, *CI* confidence interval

We wondered whether the positive association between calendar year at index date and risk of cancer was influenced in any way by MS status. When including an interaction term between MS status and index year in the model, a significant association was found (HR = 1.02, 95% CI = 1.00–1.04, *p* = 0.022), whereby the influence of index year on cancer risk was larger among MS patients than in controls. Accordingly, when stratifying individuals by MS status, the risk of any cancer remained associated with index year in MS patients (HR = 1.03, 95% CI = 1.01–1.05, *p* = 0.010), but not in controls (HR = 1.01, 95% CI 0.99–1.02, *p* = 0.144) (Table 3, Fig. 2b and c). In contrast to index year, the association of both age at index date and gender with cancer risk was similarly present in MS patients and controls (Table 3).

#### Risk of specific types of cancer

The most frequent cancer subtypes recorded after index date were breast cancer (MS: 132 [1.83%]; controls 510 [1.83%]), non-melanoma skin cancers (MS: 115 [1.14%], controls 586 [1.5%]), and prostate cancer (MS: 26 [0.9%]; controls 153 [1.37%]) (Supplementary Table 2). The risk of several cancer subtypes consistently increased with increasing age at index date. Male gender was also associated with increased risk of ear–nose–throat, lung, gastrointestinal and urinary tract, and non-melanoma skin cancers. MS status was not associated with the risk of developing any particular cancer subtype (Supplementary Table 3).

### Frequency of cancer before index date in MS patients and controls

During 2, between 2 and 5 and between 5 and 10 years before index date 72 (0.71%), 64 (1.04%) and 24 (1.04%) MS patients, and 320 (0.81%), 244 (1.04%), and 109 (1.23%) controls had a reported diagnosis of any cancer (Table 4). The risk of developing MS was similar between those individuals with vs those without a cancer code before index date (0–2 years: OR = 0.85, 95% CI = 0.66–1.10, *p* = 0.230; 2–5 years: OR = 0.99, 95% CI = 0.75–1.31, *p* = 0.979; 5–10 years: OR = 0.83, 95% CI = 0.53–1.30, *p* = 0.413, all adjusted by age and gender).

**Table 4:** Frequency of cancer among MS patients and matched controls at 0–2, 2–5 and 5–10 years before index date

Years before index date	MS patients		Matched controls	
	All cases	Cancer cases	All cases	Cancer cases
	*n*	*n* (%)	*n*	*n* (%)
0–2	10,204	72 (0.71)	39,448	320 (0.81)
2–5	6105	64 (1.04)	23,523	244 (1.04)
5–10	2309	24 (1.04)	8848	109 (1.23)

## Discussion

We observed that individuals who received a diagnosis of MS between 1990 and 2016 in the UK had an overall comparable occurrence of cancer diagnoses as compared to age, sex, and general practitioner matched subjects. A trend for a lower incidence of cancer among MS patients was actually present, but did not reach statistical significance. These results are in line with those of several studies from Northern Europe and Iran, reporting no differences in cancer risk between MS patients and the general population [3, 5, 15, 22]. Several other studies from the US, Canada, and Europe instead, found this risk to be overall decreased [7–9, 13, 17]. These findings appear even stronger in light of the expected risk of surveillance bias among individuals suffering from a chronic condition such as MS [4]. Interestingly, a recent study from Norway found an increased risk of cancer among MS patients, with a particular involvement of the respiratory, urinary, and central nervous systems [11]. In our MS cohort, however, no signals for any specific type of cancer were detected. Taken together, we can conclude that MS patients in the UK appear to be at overall similar (if not lower) risk of malignancies as compared to the general population, and this is in line with the majority of reports on this topic [3–6].

A recent systematic review of cancer incidence and prevalence in the MS population showed a significant variability and inconsistencies among results [23]. Such conflicting findings are likely related to a variety of confounding factors including different study designs, methods of data collection, time periods of the studies, differences in cancer screening programs across countries, and last but not least population-specific genetic and environmental exposures.

We noted that earlier and recent studies have generally assessed cancer occurrence irrespective of the epoch of MS diagnosis. Only one study from Sweden investigated the relationship between cancer risk and the calendar year of study entry, showing similar cancer rates in MS patients diagnosed between 1969–1980 vs 1980–2005 [7]. However, categorizing time according to pre- vs post-1980 appears rather arbitrary and the most recent years (when the majority of new immunosuppressive therapies have become available) were also not included. We, therefore, aimed at investigating a potential change in cancer occurrence across time. Interestingly, we found that, while overall cancer risk was stable over time in the control group, it consistently increased among MS patients by approximately 2% per calendar year at index date (date of a first MS code in CPRD).

It is not easy to explain these findings. It is intriguing to hypothesize that the abnormal immune response seen in MS patients may exert a protective effect against cancer development through increased immune surveillance. This may explain the historically comparable or even reduced rates of cancer among MS patients, despite the likely surveillance bias and the presence of risk factors common to MS and cancer such as smoking and obesity [24, 25]. Our results suggest, however, an apparent change in cancer diagnoses among MS patients in the UK with a variety of possible explanations. Several changes have definitely occurred between 1990 and 2016 in the field of MS, among those the introduction of several new potent therapeutic agents (many with a strong immunosuppressive effect), changes in clinical care, standardized and regular cancer screening programs and changes in lifestyle behaviours [26].

If a protective effect of MS was present against cancer, one might expect it to precede MS symptoms due to ongoing subclinical pathological immune processes, or intrinsic genetically and/or environmentally determined individual factors influencing both MS and cancer risk. To investigate this hypothesis, we also assessed cancer risk in MS individuals at 0–2, 2–5, and 5–10 years before index date, finding no differences as compared to matched controls. Similarly, a previous study by Fois et al. based on UK hospital admissions between 1963 and 1999, found no increased risk of cancer, irrespective of whether MS diagnosis preceded or followed cancer diagnosis [22]. Thormann et al. also found a similar cancer risk before a first record of MS in 8,947 Danish MS patients diagnosed between 1980 and 2005, as compared to matched individuals [17]. Despite the limited number of studies investigating cancer risk before MS diagnosis, taken together these findings suggest that any possible protective effect of MS against cancer does not anyhow appear before a definite diagnosis of MS is made [17, 22]. A caveat should be, however, mentioned, that the power of these studies might be insufficient to detect any differences due to the lower number of cancer events in younger individuals.

Our study has several limitations. Electronic medical records represent invaluable tools able to provide sample sizes that are large enough to investigate associations of small effect and subtle changes in disease rates over time. This, however, does not come without problems. MS as well as cancer diagnoses were identified when the first respective code was reported in CPRD, but this does not necessarily reflect the year of diagnosis. This is even more complicated by the fact that MS diagnostic criteria have been revised several times during the study period. Validation studies have, however, been performed supporting the reliability and quality of CPRD data and CPRD-coded diagnoses, including MS [19, 20]. Moreover, we could replicate some known associations, i.e. increasing risk of cancer with age, and increasing risk of specific cancer subtypes such as lung cancer with male gender, making our results more reliable. Second, we do not provide any data concerning possible confounding factors, such as lifestyle behaviours, sun exposure, smoking, social status, body weight and mostly disease modifying therapies, whose potential role cannot be disentangled in this context. Cases and controls were matched by GP practice, which is an indirect indicator of geographical area. Despite the evidence for a good correlation between area of residence and socioeconomic status [27], individual measures of socioeconomic status were not available and imbalances in the matching of cases and controls in this regard are possible. We also did not attempt to investigate the effect of specific treatments on cancer risk, as information regarding drugs and infusions prescribed by the treating neurologist may be absent or incomplete in a primary care database such as the CPRD.

In conclusion, we showed no significant differences in occurrence of cancer in the UK between MS patients and the general population. However, we highlight a mild progressive increase in cancer diagnoses among patients with a first record MS between 1990 and 2016, a finding that requires further investigations. It would be particularly interesting to see whether similar changes have indeed occurred in other countries than the UK. While several explanations appear to be possible, including increasing surveillance and more careful cancer screening programs in MS patients, we believe the rapid and continuous evolution of MS care, treatments and related potential secondary effects, require maximal attention in routine neurological care.

## Electronic supplementary material

Below is the link to the electronic supplementary material.

Supplementary file1 (PDF 206 kb)

Supplementary file2 (PDF 5 kb)

Supplementary file3 (PDF 13 kb)

## Data Availability

Code used for statistical analysis may be obtained from the corresponding author upon request.

## Data availability

Read codes related to cancers of interest are displayed in Supplementary Table 1. Datasets analyzed may be obtained from the corresponding author upon request.
