# Supplementary material for: Increasing cancer risk over calendar year in people with multiple sclerosis: a case–control study
Source: J Neurol. 2020 Oct 21;268(3):817–24. doi: 10.1007/s00415-020-10170-5 (PMC7914231; doi:10.1007/s00415-020-10170-5)
Supplement: Supplementary file 1 — Supplementary file1 (PDF 206 kb) [file 415_2020_10170_MOESM1_ESM.pdf]

Supplementary Table 1 List of read codes related to cancers of interest

Medcode	Readcode	Description	Cancer type
2462	B61..00	Hodgkin's disease	Hodgkin lymphoma
104291	B61..11	Hodgkin lymphoma	Hodgkin lymphoma
65489	B610.00	Hodgkin's paragranuloma	Hodgkin lymphoma
100423	B610100	Hodgkin's paragranuloma of lymph nodes of head, face, neck	Hodgkin lymphoma
98840	B610300	Hodgkin's paragranuloma of intra-abdominal lymph nodes	Hodgkin lymphoma
44196	B611.00	Hodgkin's granuloma	Hodgkin lymphoma
98909	B611100	Hodgkin's granuloma of lymph nodes of head, face and neck	Hodgkin lymphoma
64036	B612.00	Hodgkin's sarcoma	Hodgkin lymphoma
68039	B612400	Hodgkin's sarcoma of lymph nodes of axilla and upper limb	Hodgkin lymphoma
38939	B613.00	Hodgkin's disease, lymphocytic-histiocytic predominance	Hodgkin lymphoma
71142	B613000	Hodgkin's, lymphocytic-histiocytic predominance unspec site	Hodgkin lymphoma
68330	B613100	Hodgkin's, lymphocytic-histiocytic pred of head, face, neck	Hodgkin lymphoma
92245	B613200	Hodgkin's, lymphocytic-histiocytic pred intrathoracic nodes	Hodgkin lymphoma
73532	B613300	Hodgkin's, lymphocytic-histiocytic pred intra-abdominal node	Hodgkin lymphoma
93951	B613500	Hodgkin's, lymphocytic-histiocytic pred inguinal and leg	Hodgkin lymphoma
95338	B613600	Hodgkin's, lymphocytic-histiocytic pred intrapelvic nodes	Hodgkin lymphoma
106911	B613700	Hodgkin's, lymphocytic-histiocytic predominance of spleen	Hodgkin lymphoma
104743	B613800	Hodgkin's, lymphocytic-histiocytic pred of multiple sites	Hodgkin lymphoma
29876	B613z00	Hodgkin's, lymphocytic-histiocytic predominance NOS	Hodgkin lymphoma
29178	B614.00	Hodgkin's disease, nodular sclerosis	Hodgkin lymphoma
57225	B614000	Hodgkin's disease, nodular sclerosis of unspecified site	Hodgkin lymphoma
55303	B614100	Hodgkin's nodular sclerosis of head, face and neck	Hodgkin lymphoma
67506	B614200	Hodgkin's nodular sclerosis of intrathoracic lymph nodes	Hodgkin lymphoma
61149	B614300	Hodgkin's nodular sclerosis of intra-abdominal lymph nodes	Hodgkin lymphoma
65483	B614400	Hodgkin's nodular sclerosis of lymph nodes of axilla and arm	Hodgkin lymphoma
105472	B614700	Hodgkin's disease, nodular sclerosis of spleen	Hodgkin lymphoma
19140	B614800	Hodgkin's nodular sclerosis of lymph nodes of multiple sites	Hodgkin lymphoma
63054	B614z00	Hodgkin's disease, nodular sclerosis NOS	Hodgkin lymphoma
49605	B615.00	Hodgkin's disease, mixed cellularity	Hodgkin lymphoma
97863	B615000	Hodgkin's disease, mixed cellularity of unspecified site	Hodgkin lymphoma
94407	B615100	Hodgkin's mixed cellularity of lymph nodes head, face, neck	Hodgkin lymphoma
58684	B615200	Hodgkin's mixed cellularity of intrathoracic lymph nodes	Hodgkin lymphoma
108886	B615500	Hodgkin's mixed cellularity of lymph nodes inguinal and leg	Hodgkin lymphoma
94005	B615z00	Hodgkin's disease, mixed cellularity NOS	Hodgkin lymphoma
67703	B616.00	Hodgkin's disease, lymphocytic depletion	Hodgkin lymphoma
95049	B616000	Hodgkin's lymphocytic depletion of unspecified site	Hodgkin lymphoma
63625	B616400	Hodgkin's lymphocytic depletion lymph nodes axilla and arm	Hodgkin lymphoma
110563	B616500	Hodgkin's lymphocytic depletion lymph nodes inguinal and leg	Hodgkin lymphoma
101715	B616700	Hodgkin's disease, lymphocytic depletion of spleen	Hodgkin lymphoma
107032	B616800	Hodgkin's lymphocytic depletion lymph nodes multiple sites	Hodgkin lymphoma
101530	B616z00	Hodgkin's disease, lymphocytic depletion NOS	Hodgkin lymphoma
104895	B617.00	Nodular lymphocyte predominant Hodgkin lymphoma	Hodgkin lymphoma
105841	B618.00	Nodular sclerosis classical Hodgkin lymphoma	Hodgkin lymphoma

Medcode	Readcode	Description	Cancer type
108775	B619.00	Mixed cellularity classical Hodgkin lymphoma	Hodgkin lymphoma
106597	B61B.00	Lymphocyte-rich classical Hodgkin lymphoma	Hodgkin lymphoma
104484	B61C.00	Other classical Hodgkin lymphoma	Hodgkin lymphoma
53397	B61z.00	Hodgkin's disease NOS	Hodgkin lymphoma
106349	B61z.11	Hodgkin lymphoma NOS	Hodgkin lymphoma
61662	B61z000	Hodgkin's disease NOS, unspecified site	Hodgkin lymphoma
59778	B61z100	Hodgkin's disease NOS of lymph nodes of head, face and neck	Hodgkin lymphoma
59755	B61z200	Hodgkin's disease NOS of intrathoracic lymph nodes	Hodgkin lymphoma
107804	B61z300	Hodgkin's disease NOS of intra-abdominal lymph nodes	Hodgkin lymphoma
91900	B61z400	Hodgkin's disease NOS of lymph nodes of axilla and arm	Hodgkin lymphoma
99012	B61z500	Hodgkin's disease NOS of lymph nodes inguinal region and leg	Hodgkin lymphoma
94279	B61z700	Hodgkin's disease NOS of spleen	Hodgkin lymphoma
97746	B61z800	Hodgkin's disease NOS of lymph nodes of multiple sites	Hodgkin lymphoma
42461	B61zz00	Hodgkin's disease NOS	Hodgkin lymphoma
43415	ByuD000	[X]Other Hodgkin's disease	Hodgkin lymphoma
41369	B60..00	Lymphosarcoma and reticulosarcoma	Non Hodgkin lymphoma
1481	B600.00	Reticulosarcoma	Non Hodgkin lymphoma
60242	B600000	Reticulosarcoma of unspecified site	Non Hodgkin lymphoma
71031	B600100	Reticulosarcoma of lymph nodes of head, face and neck	Non Hodgkin lymphoma
70374	B600300	Reticulosarcoma of intra-abdominal lymph nodes	Non Hodgkin lymphoma
95058	B600700	Reticulosarcoma of spleen	Non Hodgkin lymphoma
99240	B600z00	Reticulosarcoma NOS	Non Hodgkin lymphoma
27416	B601.00	Lymphosarcoma	Non Hodgkin lymphoma
71625	B601000	Lymphosarcoma of unspecified site	Non Hodgkin lymphoma
71238	B601100	Lymphosarcoma of lymph nodes of head, face and neck	Non Hodgkin lymphoma
62380	B601200	Lymphosarcoma of intrathoracic lymph nodes	Non Hodgkin lymphoma
64670	B601300	Lymphosarcoma of intra-abdominal lymph nodes	Non Hodgkin lymphoma
100352	B601500	Lymphosarcoma of lymph nodes of inguinal region and leg	Non Hodgkin lymphoma
103245	B601700	Lymphosarcoma of spleen	Non Hodgkin lymphoma
104790	B601800	Lymphosarcoma of lymph nodes of multiple sites	Non Hodgkin lymphoma
63723	B601z00	Lymphosarcoma NOS	Non Hodgkin lymphoma
21402	B602.00	Burkitt's lymphoma	Non Hodgkin lymphoma
59115	B602100	Burkitt's lymphoma of lymph nodes of head, face and neck	Non Hodgkin lymphoma
100006	B602200	Burkitt's lymphoma of intrathoracic lymph nodes	Non Hodgkin lymphoma
97577	B602300	Burkitt's lymphoma of intra-abdominal lymph nodes	Non Hodgkin lymphoma

Medcode	Readcode	Description	Cancer type
92380	B602500	Burkitt's lymphoma of lymph nodes of inguinal region and leg	Non Hodgkin lymphoma
71304	B602z00	Burkitt's lymphoma NOS	Non Hodgkin lymphoma
99887	B60y.00	Other specified reticulosarcoma or lymphosarcoma	Non Hodgkin lymphoma
99951	B60z.00	Reticulosarcoma or lymphosarcoma NOS	Non Hodgkin lymphoma
33333	B62..00	Other malignant neoplasm of lymphoid and histiocytic tissue	Non Hodgkin lymphoma
5179	B620.00	Nodular lymphoma (Brill-Symmers disease)	Non Hodgkin lymphoma
66327	B620000	Nodular lymphoma of unspecified site	Non Hodgkin lymphoma
45264	B620100	Nodular lymphoma of lymph nodes of head, face and neck	Non Hodgkin lymphoma
105203	B620200	Nodular lymphoma of intrathoracic lymph nodes	Non Hodgkin lymphoma
92068	B620300	Nodular lymphoma of intra-abdominal lymph nodes	Non Hodgkin lymphoma
94995	B620500	Nodular lymphoma of lymph nodes of inguinal region and leg	Non Hodgkin lymphoma
58082	B620800	Nodular lymphoma of lymph nodes of multiple sites	Non Hodgkin lymphoma
65701	B620z00	Nodular lymphoma NOS	Non Hodgkin lymphoma
12006	B621.00	Mycosis fungoides	Non Hodgkin lymphoma
95949	B621000	Mycosis fungoides of unspecified site	Non Hodgkin lymphoma
91674	B621300	Mycosis fungoides of intra-abdominal lymph nodes	Non Hodgkin lymphoma
96379	B621400	Mycosis fungoides of lymph nodes of axilla and upper limb	Non Hodgkin lymphoma
72714	B621500	Mycosis fungoides of lymph nodes of inguinal region and leg	Non Hodgkin lymphoma
95012	B621800	Mycosis fungoides of lymph nodes of multiple sites	Non Hodgkin lymphoma
38005	B621z00	Mycosis fungoides NOS	Non Hodgkin lymphoma
35014	B622.00	Sezary's disease	Non Hodgkin lymphoma
100532	B622z00	Sezary's disease NOS	Non Hodgkin lymphoma
44267	B623.00	Malignant histiocytosis	Non Hodgkin lymphoma
69497	B623000	Malignant histiocytosis of unspecified site	Non Hodgkin lymphoma
94415	B623100	Malignant histiocytosis of lymph nodes head, face and neck	Non Hodgkin lymphoma
65642	B623300	Malignant histiocytosis of intra-abdominal lymph nodes	Non Hodgkin lymphoma
58871	B623z00	Malignant histiocytosis NOS	Non Hodgkin lymphoma
27330	B624.00	Leukaemic reticuloendotheliosis	Non Hodgkin lymphoma
5137	B624.11	Leukaemic reticuloendotheliosis	Non Hodgkin lymphoma
65122	B624000	Leukaemic reticuloendotheliosis of unspecified sites	Non Hodgkin lymphoma

Medcode	Readcode	Description	Cancer type
65123	B624300	Leukaemic reticuloend of intra-abdominal lymph nodes	Non Hodgkin lymphoma
73777	B624z00	Leukaemic reticuloendotheliosis NOS	Non Hodgkin lymphoma
34926	B625.00	Letterer-Siwe disease	Non Hodgkin lymphoma
4870	B625.11	Histiocytosis X (acute, progressive)	Non Hodgkin lymphoma
102715	B625000	Letterer-Siwe disease of unspecified sites	Non Hodgkin lymphoma
102158	B625200	Letterer-Siwe disease of intrathoracic lymph nodes	Non Hodgkin lymphoma
54083	B625800	Letterer-Siwe disease of lymph nodes of multiple sites	Non Hodgkin lymphoma
47204	B625z00	Letterer-Siwe disease NOS	Non Hodgkin lymphoma
15036	B626.00	Malignant mast cell tumours	Non Hodgkin lymphoma
103900	B626000	Mast cell malignancy of unspecified site	Non Hodgkin lymphoma
100615	B626500	Mast cell malignancy of lymph nodes inguinal region and leg	Non Hodgkin lymphoma
31324	B626800	Mast cell malignancy of lymph nodes of multiple sites	Non Hodgkin lymphoma
89657	B626z00	Malignant mast cell tumour NOS	Non Hodgkin lymphoma
3604	B627.00	Non-Hodgkin's lymphoma	Non Hodgkin lymphoma
104391	B627.11	Non-Hodgkin lymphoma	Non Hodgkin lymphoma
28639	B627000	Follicular non-Hodgkin's small cleaved cell lymphoma	Non Hodgkin lymphoma
70842	B627100	Follicular non-Hodg mixed sml cleavd & lge cell lymphoma	Non Hodgkin lymphoma
49262	B627200	Follicular non-Hodgkin's large cell lymphoma	Non Hodgkin lymphoma
50668	B627300	Diffuse non-Hodgkin's small cell (diffuse) lymphoma	Non Hodgkin lymphoma
108182	B627400	Diffuse non-Hodgkin's small cleaved cell (diffuse) lymphoma	Non Hodgkin lymphoma
50695	B627500	Diffuse non-Hodgkin mixed sml & lge cell (diffuse) lymphoma	Non Hodgkin lymphoma
53551	B627600	Diffuse non-Hodgkin's immunoblastic (diffuse) lymphoma	Non Hodgkin lymphoma
17460	B627700	Diffuse non-Hodgkin's lymphoblastic (diffuse) lymphoma	Non Hodgkin lymphoma
65180	B627800	Diffuse non-Hodgkin's lymphoma undifferentiated (diffuse)	Non Hodgkin lymphoma
95715	B627900	Mucosa-associated lymphoma	Non Hodgkin lymphoma
95545	B627911	Maltoma	Non Hodgkin lymphoma
101114	B627A00	Diffuse non-Hodgkin's large cell lymphoma	Non Hodgkin lymphoma
31576	B627B00	Other types of follicular non-Hodgkin's lymphoma	Non Hodgkin lymphoma
21549	B627C00	Follicular non-Hodgkin's lymphoma	Non Hodgkin lymphoma
17182	B627C11	Follicular lymphoma NOS	Non Hodgkin lymphoma

Medcode	Readcode	Description	Cancer type
70509	B627D00	Diffuse non-Hodgkin's centroblastic lymphoma	Non Hodgkin lymphoma
102594	B627E00	Diffuse large B-cell lymphoma	Non Hodgkin lymphoma
105966	B627F00	Extranod marg zone B-cell lymphom mucosa-assoc lymphoid tiss	Non Hodgkin lymphoma
105038	B627G00	Mediastinal (thymic) large B-cell lymphoma	Non Hodgkin lymphoma
31794	B627W00	Unspecified B-cell non-Hodgkin's lymphoma	Non Hodgkin lymphoma
39798	B627X00	Diffuse non-Hodgkin's lymphoma, unspecified	Non Hodgkin lymphoma
104152	B628.00	Follicular lymphoma	Non Hodgkin lymphoma
105889	B628000	Follicular lymphoma grade 1	Non Hodgkin lymphoma
105095	B628100	Follicular lymphoma grade 2	Non Hodgkin lymphoma
107166	B628200	Follicular lymphoma grade 3	Non Hodgkin lymphoma
105020	B628300	Follicular lymphoma grade 3a	Non Hodgkin lymphoma
107973	B628400	Follicular lymphoma grade 3b	Non Hodgkin lymphoma
106969	B628500	Diffuse follicle centre lymphoma	Non Hodgkin lymphoma
108719	B628600	Cutaneous follicle centre lymphoma	Non Hodgkin lymphoma
106063	B628700	Other types of follicular lymphoma	Non Hodgkin lymphoma
105792	B629.00	Multifocal multisystemic dissem Langerhans-cell histiocytosi	Non Hodgkin lymphoma
105335	B62A.00	Sarcoma of dendritic cells	Non Hodgkin lymphoma
110191	B62B.00	Multifocal and unisystemic Langerhans-cell histiocytosis	Non Hodgkin lymphoma
105762	B62C.00	Unifocal Langerhans-cell histiocytosis	Non Hodgkin lymphoma
105083	B62D.00	Histiocytic sarcoma	Non Hodgkin lymphoma
105085	B62E.00	T/NK-cell lymphoma	Non Hodgkin lymphoma
105559	B62E100	Anaplastic large cell lymphoma, ALK-positive	Non Hodgkin lymphoma
105955	B62E200	Anaplastic large cell lymphoma, ALK-negative	Non Hodgkin lymphoma
104862	B62E300	Cutaneous T-cell lymphoma	Non Hodgkin lymphoma
109780	B62E400	Extranodal NK/T-cell lymphoma, nasal type	Non Hodgkin lymphoma
107949	B62E500	Hepatosplenic T-cell lymphoma	Non Hodgkin lymphoma
105709	B62E600	Enteropathy-associated T-cell lymphoma	Non Hodgkin lymphoma
105925	B62E700	Subcutaneous panniculitic T-cell lymphoma	Non Hodgkin lymphoma
105375	B62E800	Blastic NK-cell lymphoma	Non Hodgkin lymphoma
105636	B62E900	Angioimmunoblastic T-cell lymphoma	Non Hodgkin lymphoma

Medcode	Readcode	Description	Cancer type
105286	B62EA00	Primary cutaneous CD30-positive T-cell proliferations	Non Hodgkin lymphoma
104934	B62Ew00	Other mature T/NK-cell lymphoma	Non Hodgkin lymphoma
106884	B62F.00	Nonfollicular lymphoma	Non Hodgkin lymphoma
106867	B62F.11	Non-follicular lymphoma	Non Hodgkin lymphoma
104386	B62F000	Small cell B-cell lymphoma	Non Hodgkin lymphoma
104620	B62F100	Mantle cell lymphoma	Non Hodgkin lymphoma
104412	B62F200	Lymphoblastic (diffuse) lymphoma	Non Hodgkin lymphoma
17887	B62x.00	Malignant lymphoma otherwise specified	Non Hodgkin lymphoma
90201	B62x000	T-zone lymphoma	Non Hodgkin lymphoma
57737	B62x100	Lymphoepithelioid lymphoma	Non Hodgkin lymphoma
12464	B62x200	Peripheral T-cell lymphoma	Non Hodgkin lymphoma
62437	B62x400	Malignant reticulosis	Non Hodgkin lymphoma
58962	B62x500	Malignant immunoproliferative small intestinal disease	Non Hodgkin lymphoma
95630	B62x600	True histiocytic lymphoma	Non Hodgkin lymphoma
44318	B62xX00	Oth and unspecif peripheral & cutaneous T-cell lymphomas	Non Hodgkin lymphoma
12335	B62y.00	Malignant lymphoma NOS	Non Hodgkin lymphoma
57427	B62y000	Malignant lymphoma NOS of unspecified site	Non Hodgkin lymphoma
50696	B62y100	Malignant lymphoma NOS of lymph nodes of head, face and neck	Non Hodgkin lymphoma
72725	B62y200	Malignant lymphoma NOS of intrathoracic lymph nodes	Non Hodgkin lymphoma
42579	B62y300	Malignant lymphoma NOS of intra-abdominal lymph nodes	Non Hodgkin lymphoma
34089	B62y400	Malignant lymphoma NOS of lymph nodes of axilla and arm	Non Hodgkin lymphoma
63105	B62y500	Malignant lymphoma NOS of lymph node inguinal region and leg	Non Hodgkin lymphoma
71262	B62y600	Malignant lymphoma NOS of intrapelvic lymph nodes	Non Hodgkin lymphoma
60092	B62y700	Malignant lymphoma NOS of spleen	Non Hodgkin lymphoma
15504	B62y800	Malignant lymphoma NOS of lymph nodes of multiple sites	Non Hodgkin lymphoma
15027	B62yz00	Malignant lymphoma NOS	Non Hodgkin lymphoma
65434	B62z.00	Malignant neoplasms of lymphoid and histiocytic tissue NOS	Non Hodgkin lymphoma
108037	B62z000	Unspec malig neop lymphoid/histiocytic of unspecified site	Non Hodgkin lymphoma
64427	B62z100	Unspec malig neop lymphoid/histiocytic lymph node head/neck	Non Hodgkin lymphoma
93384	B62z200	Unspec malig neop lymphoid/histiocytic of intrathoracic node	Non Hodgkin lymphoma

Medcode	Readcode	Description	Cancer type
103353	B62z300	Unspec malig neop lymphoid/histiocytic intra-abdominal nodes	Non Hodgkin lymphoma
107638	B62z400	Unspec malig neop lymphoid/histiocytic lymph node axilla/arm	Non Hodgkin lymphoma
71609	B62z500	Unspec malig neop lymphoid/histiocytic nodes inguinal/leg	Non Hodgkin lymphoma
109342	B62z600	Unspec malig neop lymphoid/histiocytic of intrapelvic nodes	Non Hodgkin lymphoma
101465	B62z800	Unspec malig neop lymphoid/histiocytic of multiple sites	Non Hodgkin lymphoma
95792	B62zz00	Lymphoid and histiocytic malignancy NOS	Non Hodgkin lymphoma
70716	B62zz11	Immunoproliferative neoplasm	Non Hodgkin lymphoma
67518	ByuD100	[X]Other types of follicular non-Hodgkin's lymphoma	Non Hodgkin lymphoma
98596	ByuD200	[X]Other types of diffuse non-Hodgkin's lymphoma	Non Hodgkin lymphoma
64336	ByuD300	[X]Other specified types of non-Hodgkin's lymphoma	Non Hodgkin lymphoma
64515	ByuDC00	[X]Diffuse non-Hodgkin's lymphoma, unspecified	Non Hodgkin lymphoma
109714	ByuDD00	[X]Oth and unspecif peripheral & cutaneous T-cell lymphomas	Non Hodgkin lymphoma
63375	ByuDE00	[X]Unspecified B-cell non-Hodgkin's lymphoma	Non Hodgkin lymphoma
8649	ByuDF00	[X]Non-Hodgkin's lymphoma, unspecified type	Non Hodgkin lymphoma
7940	ByuDF11	[X]Non-Hodgkin's lymphoma NOS	Non Hodgkin lymphoma
87335	B624.12	Hairy cell leukaemia	Leukaemia
37182	B63..00	Multiple myeloma and immunoproliferative neoplasms	Leukaemia
4944	B630.00	Multiple myeloma	Leukaemia
43552	B630.11	Kahler's disease	Leukaemia
15211	B630.12	Myelomatosis	Leukaemia
22158	B630000	Malignant plasma cell neoplasm, extramedullary plasmacytoma	Leukaemia
19028	B630100	Solitary myeloma	Leukaemia
21329	B630200	Plasmacytoma NOS	Leukaemia
46042	B630300	Lambda light chain myeloma	Leukaemia
104418	B630400	Solitary plasmacytoma	Leukaemia
39187	B631.00	Plasma cell leukaemia	Leukaemia
64567	B63y.00	Other immunoproliferative neoplasms	Leukaemia
43450	B63z.00	Immunoproliferative neoplasm or myeloma NOS	Leukaemia
19372	B64..00	Lymphoid leukaemia	Leukaemia
4222	B64..11	Lymphatic leukaemia	Leukaemia
4251	B640.00	Acute lymphoid leukaemia	Leukaemia
104325	B640000	B-cell acute lymphoblastic leukaemia	Leukaemia
8625	B641.00	Chronic lymphoid leukaemia	Leukaemia
27790	B641.11	Chronic lymphatic leukaemia	Leukaemia
104328	B641000	B-cell chronic lymphocytic leukaemia	Leukaemia
107017	B641011	Chronic lymphocytic leukaemia of B-cell type	Leukaemia
107052	B641100	Clinical stage A chronic lymphocytic leukaemia	Leukaemia
106924	B641200	Clinical stage B chronic lymphocytic leukaemia	Leukaemia

Medcode	Readcode	Description	Cancer type
107163	B641300	Clinical stage C chronic lymphocytic leukaemia	Leukaemia
72774	B642.00	Subacute lymphoid leukaemia	Leukaemia
49725	B64y.00	Other lymphoid leukaemia	Leukaemia
31586	B64y100	Prolymphocytic leukaemia	Leukaemia
37461	B64y200	Adult T-cell leukaemia	Leukaemia
108656	B64y300	B-cell prolymphocytic leukaemia	Leukaemia
107643	B64y400	T-cell prolymphocytic leukaemia	Leukaemia
104939	B64y500	Adult T-cell lymphoma/leukaemia (HTLV-1-associated)	Leukaemia
38331	B64yz00	Other lymphoid leukaemia NOS	Leukaemia
38914	B64z.00	Lymphoid leukaemia NOS	Leukaemia
7176	B65..00	Myeloid leukaemia	Leukaemia
4413	B650.00	Acute myeloid leukaemia	Leukaemia
10726	B651.00	Chronic myeloid leukaemia	Leukaemia
31701	B651.11	Chronic granulocytic leukaemia	Leukaemia
100786	B651000	Chronic eosinophilic leukaemia	Leukaemia
105957	B651100	Chronic myeloid leukaemia, BCR/ABL positive	Leukaemia
102783	B651200	Chronic neutrophilic leukaemia	Leukaemia
107236	B651300	Atypical chronic myeloid leukaemia, BCR/ABL negative	Leukaemia
27520	B651z00	Chronic myeloid leukaemia NOS	Leukaemia
63475	B652.00	Subacute myeloid leukaemia	Leukaemia
70724	B653.00	Myeloid sarcoma	Leukaemia
52327	B653000	Chloroma	Leukaemia
39629	B653100	Granulocytic sarcoma	Leukaemia
104788	B654.00	Acute myeloblastic leukaemia	Leukaemia
27664	B65y100	Acute promyelocytic leukaemia	Leukaemia
66089	B65yz00	Other myeloid leukaemia NOS	Leukaemia
33344	B65z.00	Myeloid leukaemia NOS	Leukaemia
35875	B66..00	Monocytic leukaemia	Leukaemia
108715	B66..11	Histiocytic leukaemia	Leukaemia
67700	B66..12	Monoblastic leukaemia	Leukaemia
19974	B660.00	Acute monocytic leukaemia	Leukaemia
27458	B661.00	Chronic monocytic leukaemia	Leukaemia
101606	B662.00	Subacute monocytic leukaemia	Leukaemia
108424	B663.00	Acute monoblastic leukaemia	Leukaemia
99015	B66y.00	Other monocytic leukaemia	Leukaemia
103645	B66yz00	Other monocytic leukaemia NOS	Leukaemia
93342	B66z.00	Monocytic leukaemia NOS	Leukaemia
37272	B67..00	Other specified leukaemia	Leukaemia
42539	B670.00	Acute erythraemia and erythroleukaemia	Leukaemia
27340	B670.11	Di Guglielmo's disease	Leukaemia
37468	B671.00	Chronic erythraemia	Leukaemia
63653	B671.11	Heilmeyer-Schoner disease	Leukaemia
57671	B672.00	Megakaryocytic leukaemia	Leukaemia
65777	B672.11	Thrombocytic leukaemia	Leukaemia
65721	B673.00	Mast cell leukaemia	Leukaemia
50858	B674.00	Acute panmyelosis	Leukaemia

Medcode	Readcode	Description	Cancer type
28276	B675.00	Acute myelofibrosis	Leukaemia
104273	B677.00	Myelodysplastic and myeloproliferative disease	Leukaemia
94174	B67y.00	Other and unspecified leukaemia	Leukaemia
72197	B67y000	Lymphosarcoma cell leukaemia	Leukaemia
99413	B67yz00	Other and unspecified leukaemia NOS	Leukaemia
30632	B67z.00	Other specified leukaemia NOS	Leukaemia
25191	B68..00	Leukaemia of unspecified cell type	Leukaemia
4072	B680.00	Acute leukaemia NOS	Leukaemia
16416	B681.00	Chronic leukaemia NOS	Leukaemia
54793	B682.00	Subacute leukaemia NOS	Leukaemia
34692	B68y.00	Other leukaemia of unspecified cell type	Leukaemia
4250	B68z.00	Leukaemia NOS	Leukaemia
20440	B69..00	Myelomonocytic leukaemia	Leukaemia
61500	B690.00	Acute myelomonocytic leukaemia	Leukaemia
22050	B691.00	Chronic myelomonocytic leukaemia	Leukaemia
104475	B692.00	Subacute myelomonocytic leukaemia	Leukaemia
105069	B693.00	Juvenile myelomonocytic leukaemia	Leukaemia
30646	B6y..00	Malignant neoplasm lymphatic or haematopoietic tissue OS	Leukaemia
6115	B6y0.00	Myeloproliferative disorder	Leukaemia
17056	B6y0.11	Myeloproliferative disease	Leukaemia
39336	B6y1.00	Myelosclerosis with myeloid metaplasia	Leukaemia
110065	B6y1.12	Osteomyelofibrosis	Leukaemia
67029	ByuD500	[X]Other lymphoid leukaemia	Leukaemia
61693	ByuD600	[X]Other myeloid leukaemia	Leukaemia
89762	ByuD700	[X]Other monocytic leukaemia	Leukaemia
89329	ByuD800	[X]Other specified leukaemias	Leukaemia
65165	ByuD900	[X]Other leukaemia of unspecified cell type	Leukaemia
18617	B51..00	Malignant neoplasm of brain	Brain cancer
10851	B51..11	Cerebral tumour-malignant	Brain cancer
15711	B510.00	Malignant neoplasm cerebrum (excluding lobes and ventricles)	Brain cancer
48073	B510000	Malignant neoplasm of basal ganglia	Brain cancer
61399	B510100	Malignant neoplasm of cerebral cortex	Brain cancer
99913	B510300	Malignant neoplasm of globus pallidus	Brain cancer
70942	B510400	Malignant neoplasm of hypothalamus	Brain cancer
62126	B510500	Malignant neoplasm of thalamus	Brain cancer
54133	B510z00	Malignant neoplasm of cerebrum NOS	Brain cancer
42426	B511.00	Malignant neoplasm of frontal lobe	Brain cancer
46792	B512.00	Malignant neoplasm of temporal lobe	Brain cancer
67236	B512000	Malignant neoplasm of hippocampus	Brain cancer
47556	B512z00	Malignant neoplasm of temporal lobe NOS	Brain cancer
19226	B513.00	Malignant neoplasm of parietal lobe	Brain cancer
39088	B514.00	Malignant neoplasm of occipital lobe	Brain cancer
52511	B515.00	Malignant neoplasm of cerebral ventricles	Brain cancer
46789	B515000	Malignant neoplasm of choroid plexus	Brain cancer
45154	B516.00	Malignant neoplasm of cerebellum	Brain cancer
44089	B517.00	Malignant neoplasm of brain stem	Brain cancer

Medcode	Readcode	Description	Cancer type
64557	B517000	Malignant neoplasm of cerebral peduncle	Brain cancer
49132	B517100	Malignant neoplasm of medulla oblongata	Brain cancer
93537	B517200	Malignant neoplasm of midbrain	Brain cancer
91240	B517300	Malignant neoplasm of pons	Brain cancer
68641	B517z00	Malignant neoplasm of brain stem NOS	Brain cancer
71139	B51y.00	Malignant neoplasm of other parts of brain	Brain cancer
59170	B51y000	Malignant neoplasm of corpus callosum	Brain cancer
65241	B51y200	Malignant neoplasm, overlapping lesion of brain	Brain cancer
100733	B51yz00	Malignant neoplasm of other part of brain NOS	Brain cancer
41520	B51z.00	Malignant neoplasm of brain NOS	Brain cancer
65458	B52..00	Malig neop of other and unspecified parts of nervous system	Brain cancer
99621	B520.00	Malignant neoplasm of cranial nerves	Brain cancer
64971	B520000	Malignant neoplasm of olfactory bulb	Brain cancer
70126	B520100	Malignant neoplasm of optic nerve	Brain cancer
65599	B520200	Malignant neoplasm of acoustic nerve	Brain cancer
101086	B520z00	Malignant neoplasm of cranial nerves NOS	Brain cancer
28919	B521.00	Malignant neoplasm of cerebral meninges	Brain cancer
109473	B521200	Malignant neoplasm of cerebral pia mater	Brain cancer
70104	B521z00	Malignant neoplasm of cerebral meninges NOS	Brain cancer
41515	ByuA100	[X]Malignant neoplasm/central nervous system, unspecified	Brain cancer
63925	ByuA200	[X]Malignant neoplasm of meninges, unspecified	Brain cancer
47633	ByuA300	[X]Malig neopl, overlap lesion brain & other part of CNS	Brain cancer
20160	B50..00	Malignant neoplasm of eye	Eye cancer
98813	B500.00	Malig neop eyeball excl conjunctiva, cornea, retina, choroid	Eye cancer
59041	B500000	Malignant neoplasm of ciliary body	Eye cancer
59381	B500100	Malignant neoplasm of iris	Eye cancer
106569	B500200	Malignant neoplasm of crystalline lens	Eye cancer
56718	B500z00	Malignant neoplasm of eyeball NOS	Eye cancer
45667	B501.00	Malignant neoplasm of orbit	Eye cancer
86996	B501000	Malignant neoplasm of connective tissue of orbit	Eye cancer
63104	B501z00	Malignant neoplasm of orbit NOS	Eye cancer
64817	B502.00	Malignant neoplasm of lacrimal gland	Eye cancer
63657	B503.00	Malignant neoplasm of conjunctiva	Eye cancer
73992	B504.00	Malignant neoplasm of cornea	Eye cancer
28069	B505.00	Malignant neoplasm of retina	Eye cancer
15991	B506.00	Malignant neoplasm of choroid	Eye cancer
71584	B507.00	Malignant neoplasm of lacrimal duct	Eye cancer
101805	B507000	Malignant neoplasm of lacrimal sac	Eye cancer
65357	B507100	Malignant neoplasm of nasolacrimal duct	Eye cancer
45922	B508.00	Malignant neoplasm, overlapping lesion of eye and adnexa	Eye cancer
40437	B50y.00	Malignant neoplasm of other specified site of eye	Eye cancer
54956	B50z.00	Malignant neoplasm of eye NOS	Eye cancer
19415	B0...00	Malignant neoplasm of lip, oral cavity and pharynx	Ear-nose-throat cancer
24374	B0...11	Carcinoma of lip, oral cavity and pharynx	Ear-nose-throat cancer
14712	B00..00	Malignant neoplasm of lip	Ear-nose-throat cancer
9984	B00..11	Carcinoma of lip	Ear-nose-throat cancer

Medcode	Readcode	Description	Cancer type
73962	B000.00	Malignant neoplasm of upper lip, vermilion border	Ear-nose-throat cancer
66270	B000000	Malignant neoplasm of upper lip, external	Ear-nose-throat cancer
50296	B000100	Malignant neoplasm of upper lip, lipstick area	Ear-nose-throat cancer
98740	B000z00	Malignant neoplasm of upper lip, vermilion border NOS	Ear-nose-throat cancer
67446	B001.00	Malignant neoplasm of lower lip, vermilion border	Ear-nose-throat cancer
66384	B001000	Malignant neoplasm of lower lip, external	Ear-nose-throat cancer
95480	B001100	Malignant neoplasm of lower lip, lipstick area	Ear-nose-throat cancer
101707	B001z00	Malignant neoplasm of lower lip, vermilion border NOS	Ear-nose-throat cancer
99493	B002.00	Malignant neoplasm of upper lip, inner aspect	Ear-nose-throat cancer
99001	B002100	Malignant neoplasm of upper lip, frenulum	Ear-nose-throat cancer
98500	B002200	Malignant neoplasm of upper lip, mucosa	Ear-nose-throat cancer
90610	B002300	Malignant neoplasm of upper lip, oral aspect	Ear-nose-throat cancer
100721	B002z00	Malignant neoplasm of upper lip, inner aspect NOS	Ear-nose-throat cancer
71147	B003.00	Malignant neoplasm of lower lip, inner aspect	Ear-nose-throat cancer
67504	B003000	Malignant neoplasm of lower lip, buccal aspect	Ear-nose-throat cancer
91843	B003100	Malignant neoplasm of lower lip, frenulum	Ear-nose-throat cancer
89909	B003200	Malignant neoplasm of lower lip, mucosa	Ear-nose-throat cancer
94441	B003300	Malignant neoplasm of lower lip, oral aspect	Ear-nose-throat cancer
96782	B003z00	Malignant neoplasm of lower lip, inner aspect NOS	Ear-nose-throat cancer
61692	B004.00	Malignant neoplasm of lip unspecified, inner aspect	Ear-nose-throat cancer
73614	B004000	Malignant neoplasm of lip unspecified, buccal aspect	Ear-nose-throat cancer
68399	B004200	Malignant neoplasm of lip unspecified, mucosa	Ear-nose-throat cancer
100144	B004300	Malignant neoplasm of lip, oral aspect	Ear-nose-throat cancer
96783	B005.00	Malignant neoplasm of commissure of lip	Ear-nose-throat cancer
18882	B006.00	Malignant neoplasm of overlapping lesion of lip	Ear-nose-throat cancer
37553	B007.00	Malignant neoplasm of lip, unspecified	Ear-nose-throat cancer
100906	B00z000	Malignant neoplasm of lip, unspecified, external	Ear-nose-throat cancer
94251	B00z100	Malignant neoplasm of lip, unspecified, lipstick area	Ear-nose-throat cancer
69761	B00zz00	Malignant neoplasm of lip, vermilion border NOS	Ear-nose-throat cancer
10283	B01..00	Malignant neoplasm of tongue	Ear-nose-throat cancer
43431	B010.00	Malignant neoplasm of base of tongue	Ear-nose-throat cancer
69671	B010.11	Malignant neoplasm of posterior third of tongue	Ear-nose-throat cancer
34409	B010000	Malignant neoplasm of base of tongue dorsal surface	Ear-nose-throat cancer
91035	B010z00	Malignant neoplasm of fixed part of tongue NOS	Ear-nose-throat cancer
43642	B011.00	Malignant neoplasm of dorsal surface of tongue	Ear-nose-throat cancer
107258	B011100	Malignant neoplasm of midline of tongue	Ear-nose-throat cancer
43781	B011z00	Malignant neoplasm of dorsum of tongue NOS	Ear-nose-throat cancer
36161	B012.00	Malignant neoplasm of tongue, tip and lateral border	Ear-nose-throat cancer
62840	B013.00	Malignant neoplasm of ventral surface of tongue	Ear-nose-throat cancer
102142	B013000	Malignant neoplasm of anterior 2/3 of tongue ventral surface	Ear-nose-throat cancer
63979	B013100	Malignant neoplasm of frenulum linguae	Ear-nose-throat cancer
38488	B013z00	Malignant neoplasm of ventral tongue surface NOS	Ear-nose-throat cancer
58121	B014.00	Malignant neoplasm of anterior 2/3 of tongue unspecified	Ear-nose-throat cancer
37096	B015.00	Malignant neoplasm of tongue, junctional zone	Ear-nose-throat cancer
24852	B016.00	Malignant neoplasm of lingual tonsil	Ear-nose-throat cancer
47205	B017.00	Malignant overlapping lesion of tongue	Ear-nose-throat cancer

Medcode	Readcode	Description	Cancer type
41530	B01y.00	Malignant neoplasm of other sites of tongue	Ear-nose-throat cancer
40557	B01z.00	Malignant neoplasm of tongue NOS	Ear-nose-throat cancer
20292	B02..00	Malignant neoplasm of major salivary glands	Ear-nose-throat cancer
4388	B020.00	Malignant neoplasm of parotid gland	Ear-nose-throat cancer
51786	B021.00	Malignant neoplasm of submandibular gland	Ear-nose-throat cancer
70928	B022.00	Malignant neoplasm of sublingual gland	Ear-nose-throat cancer
70696	B02y.00	Malignant neoplasm of other major salivary glands	Ear-nose-throat cancer
50475	B02z.00	Malignant neoplasm of major salivary gland NOS	Ear-nose-throat cancer
43400	B03..00	Malignant neoplasm of gum	Ear-nose-throat cancer
32024	B030.00	Malignant neoplasm of upper gum	Ear-nose-throat cancer
49360	B031.00	Malignant neoplasm of lower gum	Ear-nose-throat cancer
101753	B03y.00	Malignant neoplasm of other sites of gum	Ear-nose-throat cancer
93218	B03z.00	Malignant neoplasm of gum NOS	Ear-nose-throat cancer
20092	B04..00	Malignant neoplasm of floor of mouth	Ear-nose-throat cancer
45408	B040.00	Malignant neoplasm of anterior portion of floor of mouth	Ear-nose-throat cancer
45986	B041.00	Malignant neoplasm of lateral portion of floor of mouth	Ear-nose-throat cancer
17912	B042.00	Malignant neoplasm, overlapping lesion of floor of mouth	Ear-nose-throat cancer
56709	B04y.00	Malignant neoplasm of other sites of floor of mouth	Ear-nose-throat cancer
36716	B04z.00	Malignant neoplasm of floor of mouth NOS	Ear-nose-throat cancer
14792	B05..00	Malignant neoplasm of other and unspecified parts of mouth	Ear-nose-throat cancer
31364	B050.00	Malignant neoplasm of cheek mucosa	Ear-nose-throat cancer
30402	B050.11	Malignant neoplasm of buccal mucosa	Ear-nose-throat cancer
103796	B051.00	Malignant neoplasm of vestibule of mouth	Ear-nose-throat cancer
95772	B051000	Malignant neoplasm of upper buccal sulcus	Ear-nose-throat cancer
97530	B051100	Malignant neoplasm of lower buccal sulcus	Ear-nose-throat cancer
37590	B052.00	Malignant neoplasm of hard palate	Ear-nose-throat cancer
40292	B053.00	Malignant neoplasm of soft palate	Ear-nose-throat cancer
37516	B054.00	Malignant neoplasm of uvula	Ear-nose-throat cancer
70819	B055.00	Malignant neoplasm of palate unspecified	Ear-nose-throat cancer
96003	B055000	Malignant neoplasm of junction of hard and soft palate	Ear-nose-throat cancer
69951	B055100	Malignant neoplasm of roof of mouth	Ear-nose-throat cancer
28559	B055z00	Malignant neoplasm of palate NOS	Ear-nose-throat cancer
37724	B056.00	Malignant neoplasm of retromolar area	Ear-nose-throat cancer
10314	B057.00	Overlapping lesion of other and unspecified parts of mouth	Ear-nose-throat cancer
37916	B05y.00	Malignant neoplasm of other specified mouth parts	Ear-nose-throat cancer
55015	B05z.00	Malignant neoplasm of mouth NOS	Ear-nose-throat cancer
37549	B05z000	Kaposi's sarcoma of palate	Ear-nose-throat cancer
22893	B06..00	Malignant neoplasm of oropharynx	Ear-nose-throat cancer
16241	B060.00	Malignant neoplasm of tonsil	Ear-nose-throat cancer
26448	B060000	Malignant neoplasm of faucial tonsil	Ear-nose-throat cancer
101988	B060100	Malignant neoplasm of palatine tonsil	Ear-nose-throat cancer
102151	B060200	Malignant neoplasm of overlapping lesion of tonsil	Ear-nose-throat cancer
53884	B060z00	Malignant neoplasm tonsil NOS	Ear-nose-throat cancer
24397	B061.00	Malignant neoplasm of tonsillar fossa	Ear-nose-throat cancer
55066	B062.00	Malignant neoplasm of tonsillar pillar	Ear-nose-throat cancer
51926	B062000	Malignant neoplasm of faucial pillar	Ear-nose-throat cancer

Medcode	Readcode	Description	Cancer type
99185	B062100	Malignant neoplasm of glossopalatine fold	Ear-nose-throat cancer
61510	B062200	Malignant neoplasm of palatoglossal arch	Ear-nose-throat cancer
93842	B062300	Malignant neoplasm of palatopharyngeal arch	Ear-nose-throat cancer
100002	B062z00	Malignant neoplasm of tonsillar fossa NOS	Ear-nose-throat cancer
39554	B063.00	Malignant neoplasm of vallecula	Ear-nose-throat cancer
46728	B064.00	Malignant neoplasm of anterior epiglottis	Ear-nose-throat cancer
26134	B064000	Malignant neoplasm of epiglottis, free border	Ear-nose-throat cancer
91895	B064100	Malignant neoplasm of glossoepiglottic fold	Ear-nose-throat cancer
73439	B064z00	Malignant neoplasm of anterior epiglottis NOS	Ear-nose-throat cancer
48519	B065.00	Malignant neoplasm of junctional region of epiglottis	Ear-nose-throat cancer
56355	B066.00	Malignant neoplasm of lateral wall of oropharynx	Ear-nose-throat cancer
90124	B067.00	Malignant neoplasm of posterior wall of oropharynx	Ear-nose-throat cancer
67323	B06y.00	Malignant neoplasm of oropharynx, other specified sites	Ear-nose-throat cancer
91037	B06yz00	Malignant neoplasm of other specified site of oropharynx NOS	Ear-nose-throat cancer
43200	B06z.00	Malignant neoplasm of oropharynx NOS	Ear-nose-throat cancer
24675	B07..00	Malignant neoplasm of nasopharynx	Ear-nose-throat cancer
94390	B070.00	Malignant neoplasm of roof of nasopharynx	Ear-nose-throat cancer
95429	B071.00	Malignant neoplasm of posterior wall of nasopharynx	Ear-nose-throat cancer
33388	B071000	Malignant neoplasm of adenoid	Ear-nose-throat cancer
46548	B071100	Malignant neoplasm of pharyngeal tonsil	Ear-nose-throat cancer
96869	B071z00	Malignant neoplasm of posterior wall of nasopharynx NOS	Ear-nose-throat cancer
59004	B072.00	Malignant neoplasm of lateral wall of nasopharynx	Ear-nose-throat cancer
37940	B072000	Malignant neoplasm of pharyngeal recess	Ear-nose-throat cancer
102205	B072z00	Malignant neoplasm of lateral wall of nasopharynx NOS	Ear-nose-throat cancer
44139	B073.00	Malignant neoplasm of anterior wall of nasopharynx	Ear-nose-throat cancer
106915	B073100	Malignant neoplasm of nasopharyngeal soft palate surface	Ear-nose-throat cancer
99386	B073200	Malignant neoplasm posterior margin nasal septum and choanae	Ear-nose-throat cancer
100918	B073z00	Malignant neoplasm of anterior wall of nasopharynx NOS	Ear-nose-throat cancer
66422	B074.00	Malignant neoplasm, overlapping lesion of nasopharynx	Ear-nose-throat cancer
55630	B07y.00	Malignant neoplasm of other specified site of nasopharynx	Ear-nose-throat cancer
28665	B07z.00	Malignant neoplasm of nasopharynx NOS	Ear-nose-throat cancer
34012	B08..00	Malignant neoplasm of hypopharynx	Ear-nose-throat cancer
43548	B080.00	Malignant neoplasm of postcricoid region	Ear-nose-throat cancer
39897	B081.00	Malignant neoplasm of pyriform sinus	Ear-nose-throat cancer
57248	B082.00	Malignant neoplasm aryepiglottic fold, hypopharyngeal aspect	Ear-nose-throat cancer
64462	B083.00	Malignant neoplasm of posterior pharynx	Ear-nose-throat cancer
88362	B08y.00	Malignant neoplasm of other specified hypopharyngeal site	Ear-nose-throat cancer
28451	B08z.00	Malignant neoplasm of hypopharynx NOS	Ear-nose-throat cancer
46114	B0z..00	Malig neop other/ill-defined sites lip, oral cavity, pharynx	Ear-nose-throat cancer
16297	B0z0.00	Malignant neoplasm of pharynx unspecified	Ear-nose-throat cancer
95016	B0z1.00	Malignant neoplasm of Waldeyer's ring	Ear-nose-throat cancer
39084	B0z2.00	Malignant neoplasm of laryngopharynx	Ear-nose-throat cancer
49758	B0zy.00	Malignant neoplasm of other sites lip, oral cavity, pharynx	Ear-nose-throat cancer
39430	B0zz.00	Malignant neoplasm of lip, oral cavity and pharynx NOS	Ear-nose-throat cancer
23389	B200.00	Malignant neoplasm of nasal cavities	Ear-nose-throat cancer
71204	B200000	Malignant neoplasm of cartilage of nose	Ear-nose-throat cancer

Medcode	Readcode	Description	Cancer type
98911	B200100	Malignant neoplasm of nasal conchae	Ear-nose-throat cancer
62761	B200200	Malignant neoplasm of septum of nose	Ear-nose-throat cancer
62182	B200300	Malignant neoplasm of vestibule of nose	Ear-nose-throat cancer
42856	B200z00	Malignant neoplasm of nasal cavities NOS	Ear-nose-throat cancer
24456	B201.00	Malig neop auditory tube, middle ear and mastoid air cells	Ear-nose-throat cancer
107916	B201000	Malignant neoplasm of auditory (Eustachian) tube	Ear-nose-throat cancer
98537	B201100	Malignant neoplasm of tympanic cavity	Ear-nose-throat cancer
54613	B201200	Malignant neoplasm of tympanic antrum	Ear-nose-throat cancer
71946	B201300	Malignant neoplasm of mastoid air cells	Ear-nose-throat cancer
73537	B201z00	Malig neop auditory tube, middle ear, mastoid air cells NOS	Ear-nose-throat cancer
32174	B202.00	Malignant neoplasm of maxillary sinus	Ear-nose-throat cancer
54636	B203.00	Malignant neoplasm of ethmoid sinus	Ear-nose-throat cancer
15684	B204.00	Malignant neoplasm of frontal sinus	Ear-nose-throat cancer
65215	B205.00	Malignant neoplasm of sphenoidal sinus	Ear-nose-throat cancer
39590	B206.00	Malignant neoplasm, overlapping lesion of accessory sinuses	Ear-nose-throat cancer
96971	B20y.00	Malig neop other site nasal cavity, middle ear and sinuses	Ear-nose-throat cancer
55246	B20z.00	Malignant neoplasm of accessory sinus NOS	Ear-nose-throat cancer
95390	B800.00	Carcinoma in situ of lip, oral cavity and pharynx	Ear-nose-throat cancer
37505	B800.11	Carcinoma in situ of oral cavity	Ear-nose-throat cancer
42129	B800.12	Carcinoma in situ of pharynx	Ear-nose-throat cancer
47737	B800000	Carcinoma in situ of lip	Ear-nose-throat cancer
27944	B800100	Carcinoma in situ of tongue	Ear-nose-throat cancer
50288	B800200	Carcinoma in situ of salivary glands	Ear-nose-throat cancer
57866	B800300	Carcinoma in situ of gums	Ear-nose-throat cancer
24801	B800400	Carcinoma in situ of floor of mouth	Ear-nose-throat cancer
34823	B800500	Carcinoma in situ of cheek	Ear-nose-throat cancer
30966	B800600	Carcinoma in situ of palate	Ear-nose-throat cancer
36104	B800700	Carcinoma in situ of nasopharynx	Ear-nose-throat cancer
50419	B800800	Carcinoma in situ of oropharynx	Ear-nose-throat cancer
44663	B800900	Carcinoma in situ of hypopharynx	Ear-nose-throat cancer
37187	B800z00	Carcinoma in situ of lip, oral cavity and pharynx NOS	Ear-nose-throat cancer
58973	Byu0.00	[X]Malignant neoplasm of lip, oral cavity and pharynx	Ear-nose-throat cancer
319	B21..00	Malignant neoplasm of larynx	Lung and respiratory cancer
318	B210.00	Malignant neoplasm of glottis	Lung and respiratory cancer
26165	B211.00	Malignant neoplasm of supraglottis	Lung and respiratory cancer
22441	B212.00	Malignant neoplasm of subglottis	Lung and respiratory cancer
43111	B213.00	Malignant neoplasm of laryngeal cartilage	Lung and respiratory cancer
63460	B213000	Malignant neoplasm of arytenoid cartilage	Lung and respiratory cancer
37805	B213100	Malignant neoplasm of cricoid cartilage	Lung and respiratory cancer
107878	B213200	Malignant neoplasm of cuneiform cartilage	Lung and respiratory cancer
47862	B213300	Malignant neoplasm of thyroid cartilage	Lung and respiratory cancer

Medcode	Readcode	Description	Cancer type
97332	B213z00	Malignant neoplasm of laryngeal cartilage NOS	Lung and respiratory cancer
50579	B214.00	Malignant neoplasm, overlapping lesion of larynx	Lung and respiratory cancer
55374	B215.00	Malignant neoplasm of epiglottis NOS	Lung and respiratory cancer
26813	B21y.00	Malignant neoplasm of larynx, other specified site	Lung and respiratory cancer
9237	B21z.00	Malignant neoplasm of larynx NOS	Lung and respiratory cancer
13243	B22..00	Malignant neoplasm of trachea, bronchus and lung	Lung and respiratory cancer
15221	B220.00	Malignant neoplasm of trachea	Lung and respiratory cancer
103946	B220100	Malignant neoplasm of mucosa of trachea	Lung and respiratory cancer
37810	B220z00	Malignant neoplasm of trachea NOS	Lung and respiratory cancer
12870	B221.00	Malignant neoplasm of main bronchus	Lung and respiratory cancer
17391	B221000	Malignant neoplasm of carina of bronchus	Lung and respiratory cancer
33444	B221100	Malignant neoplasm of hilus of lung	Lung and respiratory cancer
21698	B221z00	Malignant neoplasm of main bronchus NOS	Lung and respiratory cancer
10358	B222.00	Malignant neoplasm of upper lobe, bronchus or lung	Lung and respiratory cancer
20170	B222.11	Pancoast's syndrome	Lung and respiratory cancer
31700	B222000	Malignant neoplasm of upper lobe bronchus	Lung and respiratory cancer
25886	B222100	Malignant neoplasm of upper lobe of lung	Lung and respiratory cancer
44169	B222z00	Malignant neoplasm of upper lobe, bronchus or lung NOS	Lung and respiratory cancer
31268	B223.00	Malignant neoplasm of middle lobe, bronchus or lung	Lung and respiratory cancer
41523	B223000	Malignant neoplasm of middle lobe bronchus	Lung and respiratory cancer
39923	B223100	Malignant neoplasm of middle lobe of lung	Lung and respiratory cancer
54134	B223z00	Malignant neoplasm of middle lobe, bronchus or lung NOS	Lung and respiratory cancer
31188	B224.00	Malignant neoplasm of lower lobe, bronchus or lung	Lung and respiratory cancer
18678	B224000	Malignant neoplasm of lower lobe bronchus	Lung and respiratory cancer
12582	B224100	Malignant neoplasm of lower lobe of lung	Lung and respiratory cancer
42566	B224z00	Malignant neoplasm of lower lobe, bronchus or lung NOS	Lung and respiratory cancer
36371	B225.00	Malignant neoplasm of overlapping lesion of bronchus & lung	Lung and respiratory cancer
7484	B226.00	Mesothelioma	Lung and respiratory cancer
38961	B22y.00	Malignant neoplasm of other sites of bronchus or lung	Lung and respiratory cancer
3903	B22z.00	Malignant neoplasm of bronchus or lung NOS	Lung and respiratory cancer

Medcode	Readcode	Description	Cancer type
2587	B22z.11	Lung cancer	Lung and respiratory cancer
31573	B23..00	Malignant neoplasm of pleura	Lung and respiratory cancer
67107	B230.00	Malignant neoplasm of parietal pleura	Lung and respiratory cancer
106194	B231.00	Malignant neoplasm of visceral pleura	Lung and respiratory cancer
9600	B232.00	Mesothelioma of pleura	Lung and respiratory cancer
98104	B23y.00	Malignant neoplasm of other specified pleura	Lung and respiratory cancer
34742	B23z.00	Malignant neoplasm of pleura NOS	Lung and respiratory cancer
64050	B81..00	Carcinoma in situ of respiratory system	Lung and respiratory cancer
11403	B810.00	Carcinoma in situ of larynx	Lung and respiratory cancer
35772	B810000	Carcinoma in situ of thyroid cartilage	Lung and respiratory cancer
36948	B810100	Carcinoma in situ of cricoid cartilage	Lung and respiratory cancer
53460	B810200	Carcinoma in situ of epiglottis	Lung and respiratory cancer
65953	B810300	Carcinoma in situ of arytenoid cartilage	Lung and respiratory cancer
31860	B810600	Carcinoma in situ of aryepiglottic fold	Lung and respiratory cancer
73076	B810700	Carcinoma in situ of vestibular fold	Lung and respiratory cancer
7697	B810800	Carcinoma in situ of vocal fold-glottis	Lung and respiratory cancer
10375	B810811	Carcinoma in situ of glottis	Lung and respiratory cancer
53882	B810z00	Carcinoma in situ of larynx NOS	Lung and respiratory cancer
51714	B811.00	Carcinoma in situ of trachea	Lung and respiratory cancer
9267	B812.00	Carcinoma in situ of bronchus and lung	Lung and respiratory cancer
49159	B812000	Carcinoma in situ of carina of bronchus	Lung and respiratory cancer
35058	B812100	Carcinoma in situ of main bronchus	Lung and respiratory cancer
37579	B812200	Carcinoma in situ of upper lobe bronchus and lung	Lung and respiratory cancer
47897	B812300	Carcinoma in situ of middle lobe bronchus and lung	Lung and respiratory cancer
52373	B812400	Carcinoma in situ of lower lobe bronchus and lung	Lung and respiratory cancer
25372	B812z00	Carcinoma in situ of bronchus or lung NOS	Lung and respiratory cancer
97954	B81y.00	Carcinoma in situ of other specified part respiratory system	Lung and respiratory cancer
59426	B81y.11	Carcinoma in situ of nasal sinuses	Lung and respiratory cancer
46497	B81y000	Carcinoma in situ of pleura	Lung and respiratory cancer
95559	B81yz00	Carcinoma in situ of specified parts respiratory system NOS	Lung and respiratory cancer

Medcode	Readcode	Description	Cancer type
62610	B81z.00	Carcinoma in situ of respiratory organ NOS	Lung and respiratory cancer
35325	Byu2.00	[X]Malignant neoplasm of respiratory and intrathoracic orga	Lung and respiratory cancer
40595	Byu2000	[X]Malignant neoplasm of bronchus or lung, unspecified	Lung and respiratory cancer
21715	Byu5011	[X]Mesothelioma of lung	Lung and respiratory cancer
100781	ByuF300	[X]Carcinoma in situ of other parts of respiratory system	Lung and respiratory cancer
15709	B1...00	Malignant neoplasm of digestive organs and peritoneum	Gastro-intestinal cancer
3357	B1...11	Carcinoma of digestive organs and peritoneum	Gastro-intestinal cancer
1062	B10..00	Malignant neoplasm of oesophagus	Gastro-intestinal cancer
61695	B100.00	Malignant neoplasm of cervical oesophagus	Gastro-intestinal cancer
41362	B101.00	Malignant neoplasm of thoracic oesophagus	Gastro-intestinal cancer
63470	B102.00	Malignant neoplasm of abdominal oesophagus	Gastro-intestinal cancer
50789	B103.00	Malignant neoplasm of upper third of oesophagus	Gastro-intestinal cancer
54171	B104.00	Malignant neoplasm of middle third of oesophagus	Gastro-intestinal cancer
42416	B105.00	Malignant neoplasm of lower third of oesophagus	Gastro-intestinal cancer
67497	B106.00	Malignant neoplasm, overlapping lesion of oesophagus	Gastro-intestinal cancer
98142	B107.00	Siewert type I adenocarcinoma	Gastro-intestinal cancer
53591	B10y.00	Malignant neoplasm of other specified part of oesophagus	Gastro-intestinal cancer
30700	B10z.00	Malignant neoplasm of oesophagus NOS	Gastro-intestinal cancer
4865	B10z.11	Oesophageal cancer	Gastro-intestinal cancer
8386	B11..00	Malignant neoplasm of stomach	Gastro-intestinal cancer
10368	B11..11	Gastric neoplasm	Gastro-intestinal cancer
32022	B110.00	Malignant neoplasm of cardia of stomach	Gastro-intestinal cancer
100584	B110000	Malignant neoplasm of cardiac orifice of stomach	Gastro-intestinal cancer
22894	B110100	Malignant neoplasm of cardio-oesophageal junction of stomach	Gastro-intestinal cancer
94278	B110111	Malignant neoplasm of gastro-oesophageal junction	Gastro-intestinal cancer
37859	B110z00	Malignant neoplasm of cardia of stomach NOS	Gastro-intestinal cancer
21620	B111.00	Malignant neoplasm of pylorus of stomach	Gastro-intestinal cancer
48237	B111000	Malignant neoplasm of prepylorus of stomach	Gastro-intestinal cancer
41215	B111100	Malignant neoplasm of pyloric canal of stomach	Gastro-intestinal cancer
59092	B111z00	Malignant neoplasm of pylorus of stomach NOS	Gastro-intestinal cancer

Medcode	Readcode	Description	Cancer type
19318	B112.00	Malignant neoplasm of pyloric antrum of stomach	Gastro-intestinal cancer
32362	B113.00	Malignant neoplasm of fundus of stomach	Gastro-intestinal cancer
43572	B114.00	Malignant neoplasm of body of stomach	Gastro-intestinal cancer
42193	B115.00	Malignant neoplasm of lesser curve of stomach unspecified	Gastro-intestinal cancer
55434	B116.00	Malignant neoplasm of greater curve of stomach unspecified	Gastro-intestinal cancer
51690	B117.00	Malignant neoplasm, overlapping lesion of stomach	Gastro-intestinal cancer
97499	B118.00	Siewert type II adenocarcinoma	Gastro-intestinal cancer
96094	B119.00	Siewert type III adenocarcinoma	Gastro-intestinal cancer
55019	B11y.00	Malignant neoplasm of other specified site of stomach	Gastro-intestinal cancer
65312	B11y000	Malignant neoplasm of anterior wall of stomach NEC	Gastro-intestinal cancer
96802	B11y100	Malignant neoplasm of posterior wall of stomach NEC	Gastro-intestinal cancer
65372	B11yz00	Malignant neoplasm of other specified site of stomach NOS	Gastro-intestinal cancer
14800	B11z.00	Malignant neoplasm of stomach NOS	Gastro-intestinal cancer
6806	B12..00	Malignant neoplasm of small intestine and duodenum	Gastro-intestinal cancer
18613	B120.00	Malignant neoplasm of duodenum	Gastro-intestinal cancer
43479	B121.00	Malignant neoplasm of jejunum	Gastro-intestinal cancer
33871	B122.00	Malignant neoplasm of ileum	Gastro-intestinal cancer
63995	B123.00	Malignant neoplasm of Meckel's diverticulum	Gastro-intestinal cancer
66166	B124.00	Malignant neoplasm, overlapping lesion of small intestine	Gastro-intestinal cancer
99896	B12y.00	Malignant neoplasm of other specified site small intestine	Gastro-intestinal cancer
43390	B12z.00	Malignant neoplasm of small intestine NOS	Gastro-intestinal cancer
1220	B13..00	Malignant neoplasm of colon	Gastro-intestinal cancer
9088	B130.00	Malignant neoplasm of hepatic flexure of colon	Gastro-intestinal cancer
6935	B131.00	Malignant neoplasm of transverse colon	Gastro-intestinal cancer
10864	B132.00	Malignant neoplasm of descending colon	Gastro-intestinal cancer
2815	B133.00	Malignant neoplasm of sigmoid colon	Gastro-intestinal cancer
3811	B134.00	Malignant neoplasm of caecum	Gastro-intestinal cancer
22163	B134.11	Carcinoma of caecum	Gastro-intestinal cancer
18632	B135.00	Malignant neoplasm of appendix	Gastro-intestinal cancer
10946	B136.00	Malignant neoplasm of ascending colon	Gastro-intestinal cancer

Medcode	Readcode	Description	Cancer type
18619	B137.00	Malignant neoplasm of splenic flexure of colon	Gastro-intestinal cancer
93478	B138.00	Malignant neoplasm, overlapping lesion of colon	Gastro-intestinal cancer
101700	B139.00	Hereditary nonpolyposis colon cancer	Gastro-intestinal cancer
48231	B13y.00	Malignant neoplasm of other specified sites of colon	Gastro-intestinal cancer
28163	B13z.00	Malignant neoplasm of colon NOS	Gastro-intestinal cancer
9118	B13z.11	Colonic cancer	Gastro-intestinal cancer
35357	B14..00	Malignant neoplasm of rectum, rectosigmoid junction and anus	Gastro-intestinal cancer
27855	B140.00	Malignant neoplasm of rectosigmoid junction	Gastro-intestinal cancer
1800	B141.00	Malignant neoplasm of rectum	Gastro-intestinal cancer
7219	B141.11	Carcinoma of rectum	Gastro-intestinal cancer
5901	B141.12	Rectal carcinoma	Gastro-intestinal cancer
24370	B142.00	Malignant neoplasm of anal canal	Gastro-intestinal cancer
9491	B142.11	Anal carcinoma	Gastro-intestinal cancer
46159	B142000	Malignant neoplasm of cloacogenic zone	Gastro-intestinal cancer
27897	B143.00	Malignant neoplasm of anus unspecified	Gastro-intestinal cancer
55659	B14y.00	Malig neop other site rectum, rectosigmoid junction and anus	Gastro-intestinal cancer
50974	B14z.00	Malignant neoplasm rectum,rectosigmoid junction and anus NOS	Gastro-intestinal cancer
17559	B1z0.00	Malignant neoplasm of intestinal tract, part unspecified	Gastro-intestinal cancer
11628	B1z0.11	Cancer of bowel	Gastro-intestinal cancer
94776	B1z2.00	Malignant neoplasm, overlapping lesion of digestive system	Gastro-intestinal cancer
56918	B1zy.00	Malignant neoplasm other spec digestive tract and peritoneum	Gastro-intestinal cancer
51255	B1zz.00	Malignant neoplasm of digestive tract and peritoneum NOS	Gastro-intestinal cancer
8244	B801.00	Carcinoma in situ of oesophagus	Gastro-intestinal cancer
99155	B801000	Carcinoma in situ of upper 1/3 oesophagus	Gastro-intestinal cancer
64274	B801100	Carcinoma in situ of middle 1/3 oesophagus	Gastro-intestinal cancer
56077	B801200	Carcinoma in situ of lower 1/3 oesophagus	Gastro-intestinal cancer
44228	B801z00	Carcinoma in situ of oesophagus NOS	Gastro-intestinal cancer
17093	B802.00	Carcinoma in situ of stomach	Gastro-intestinal cancer
17258	B802000	Carcinoma in situ of cardia of stomach	Gastro-intestinal cancer
72947	B802100	Carcinoma in situ of fundus of stomach	Gastro-intestinal cancer

Medcode	Readcode	Description	Cancer type
63087	B802200	Carcinoma in situ of body of stomach	Gastro-intestinal cancer
51748	B802300	Carcinoma in situ of pyloric antrum	Gastro-intestinal cancer
58883	B802400	Carcinoma in situ of pyloric canal	Gastro-intestinal cancer
37774	B802z00	Carcinoma in situ of stomach NOS	Gastro-intestinal cancer
6903	B803.00	Carcinoma in situ of colon	Gastro-intestinal cancer
39080	B803000	Carcinoma in situ of hepatic flexure of colon	Gastro-intestinal cancer
37125	B803100	Carcinoma in situ of transverse colon	Gastro-intestinal cancer
47667	B803200	Carcinoma in situ of descending colon	Gastro-intestinal cancer
17144	B803300	Carcinoma in situ of sigmoid colon	Gastro-intestinal cancer
16916	B803400	Carcinoma in situ of caecum	Gastro-intestinal cancer
47656	B803500	Carcinoma in situ of appendix	Gastro-intestinal cancer
31893	B803600	Carcinoma in situ of ascending colon	Gastro-intestinal cancer
22699	B803700	Carcinoma in situ of splenic flexure of colon	Gastro-intestinal cancer
105228	B803800	High grade dysplasia of colon	Gastro-intestinal cancer
33561	B803z00	Carcinoma in situ of colon NOS	Gastro-intestinal cancer
60477	B804.00	Carcinoma in situ of rectum and rectosigmoid junction	Gastro-intestinal cancer
27811	B804000	Carcinoma in situ of rectosigmoid junction	Gastro-intestinal cancer
29975	B804100	Carcinoma in situ of rectum	Gastro-intestinal cancer
38883	B804z00	Carcinoma in situ of rectum or rectosigmoid junction NOS	Gastro-intestinal cancer
51054	B805.00	Carcinoma in situ of anal canal	Gastro-intestinal cancer
34094	B805000	Anal intraepithelial neoplasia grade III	Gastro-intestinal cancer
12273	B806.00	Carcinoma in situ of anus NOS	Gastro-intestinal cancer
22392	B807.00	Carcinoma in situ of other and unspecified small intestine	Gastro-intestinal cancer
45070	B807000	Carcinoma in situ of duodenum	Gastro-intestinal cancer
63804	B807100	Carcinoma in situ of jejunum	Gastro-intestinal cancer
45217	B807200	Carcinoma in situ of ileum	Gastro-intestinal cancer
100183	B807300	Carcinoma in situ of Meckel's diverticulum	Gastro-intestinal cancer
70728	B807z00	Carcinoma in situ other and unspecified small intestine NOS	Gastro-intestinal cancer
35180	Byu1.00	[X]Malignant neoplasm of digestive organs	Gastro-intestinal cancer
45766	Byu1200	[X]Malignant neoplasm of intestinal tract, part unspecified	Gastro-intestinal cancer

Medcode	Readcode	Description	Cancer type
49292	Byu1300	[X]Malignant neoplsm/ill-defin sites within digestive system	Gastro-intestinal cancer
102708	ByuF100	[X]Carcinoma in situ of other specified digestive organs	Gastro-intestinal cancer
8918	B15..00	Malignant neoplasm of liver and intrahepatic bile ducts	Liver and and biliopancreatic cancer
25535	B150.00	Primary malignant neoplasm of liver	Liver and and biliopancreatic cancer
16126	B150000	Primary carcinoma of liver	Liver and and biliopancreatic cancer
31210	B150100	Hepatoblastoma of liver	Liver and and biliopancreatic cancer
68410	B150200	Primary angiosarcoma of liver	Liver and and biliopancreatic cancer
22187	B150300	Hepatocellular carcinoma	Liver and and biliopancreatic cancer
44399	B150z00	Primary malignant neoplasm of liver NOS	Liver and and biliopancreatic cancer
16915	B151.00	Malignant neoplasm of intrahepatic bile ducts	Liver and and biliopancreatic cancer
65124	B151000	Malignant neoplasm of interlobular bile ducts	Liver and and biliopancreatic cancer
89593	B151200	Malignant neoplasm of intrahepatic biliary passages	Liver and and biliopancreatic cancer
58088	B151400	Malignant neoplasm of intrahepatic gall duct	Liver and and biliopancreatic cancer
61643	B151z00	Malignant neoplasm of intrahepatic bile ducts NOS	Liver and and biliopancreatic cancer
26393	B152.00	Malignant neoplasm of liver unspecified	Liver and and biliopancreatic cancer
36147	B153.00	Secondary malignant neoplasm of liver	Liver and and biliopancreatic cancer
38978	B15z.00	Malignant neoplasm of liver and intrahepatic bile ducts NOS	Liver and and biliopancreatic cancer
54103	B16..00	Malignant neoplasm gallbladder and extrahepatic bile ducts	Liver and and biliopancreatic cancer
16105	B160.00	Malignant neoplasm of gallbladder	Liver and and biliopancreatic cancer
31393	B160.11	Carcinoma gallbladder	Liver and and biliopancreatic cancer
23433	B161.00	Malignant neoplasm of extrahepatic bile ducts	Liver and and biliopancreatic cancer
72445	B161000	Malignant neoplasm of cystic duct	Liver and and biliopancreatic cancer
52537	B161100	Malignant neoplasm of hepatic duct	Liver and and biliopancreatic cancer
7982	B161200	Malignant neoplasm of common bile duct	Liver and and biliopancreatic cancer
36495	B161211	Carcinoma common bile duct	Liver and and biliopancreatic cancer
105613	B161300	Malignant neoplasm of sphincter of Oddi	Liver and and biliopancreatic cancer
74896	B161z00	Malignant neoplasm of extrahepatic bile ducts NOS	Liver and and biliopancreatic cancer
10949	B162.00	Malignant neoplasm of ampulla of Vater	Liver and and biliopancreatic cancer
35039	B163.00	Malignant neoplasm, overlapping lesion of biliary tract	Liver and and biliopancreatic cancer
60312	B16y.00	Malignant neoplasm other gallbladder/extrahepatic bile duct	Liver and and biliopancreatic cancer

Medcode	Readcode	Description	Cancer type
15907	B16z.00	Malignant neoplasm gallbladder/extrahepatic bile ducts NOS	Liver and and biliopancreatic cancer
8166	B17..00	Malignant neoplasm of pancreas	Liver and and biliopancreatic cancer
8771	B170.00	Malignant neoplasm of head of pancreas	Liver and and biliopancreatic cancer
40810	B171.00	Malignant neoplasm of body of pancreas	Liver and and biliopancreatic cancer
39870	B172.00	Malignant neoplasm of tail of pancreas	Liver and and biliopancreatic cancer
35535	B173.00	Malignant neoplasm of pancreatic duct	Liver and and biliopancreatic cancer
35795	B174.00	Malignant neoplasm of Islets of Langerhans	Liver and and biliopancreatic cancer
97875	B175.00	Malignant neoplasm, overlapping lesion of pancreas	Liver and and biliopancreatic cancer
109782	B176.00	Somatostatinoma of pancreas	Liver and and biliopancreatic cancer
48537	B17y.00	Malignant neoplasm of other specified sites of pancreas	Liver and and biliopancreatic cancer
96635	B17y000	Malignant neoplasm of ectopic pancreatic tissue	Liver and and biliopancreatic cancer
95783	B17yz00	Malignant neoplasm of specified site of pancreas NOS	Liver and and biliopancreatic cancer
34388	B17z.00	Malignant neoplasm of pancreas NOS	Liver and and biliopancreatic cancer
66673	B808.00	Carcinoma in situ of liver and biliary system	Liver and and biliopancreatic cancer
51934	B808.11	Carcinoma in situ of biliary system	Liver and and biliopancreatic cancer
25310	B808000	Carcinoma in situ of liver	Liver and and biliopancreatic cancer
99580	B808100	Carcinoma in situ of intrahepatic bile ducts	Liver and and biliopancreatic cancer
37501	B808200	Carcinoma in situ of hepatic duct	Liver and and biliopancreatic cancer
46594	B808300	Carcinoma in situ of gall bladder	Liver and and biliopancreatic cancer
73164	B808400	Carcinoma in situ of cystic duct	Liver and and biliopancreatic cancer
64089	B808500	Carcinoma in situ of common bile duct	Liver and and biliopancreatic cancer
21792	B808600	Carcinoma in situ of ampulla of Vater	Liver and and biliopancreatic cancer
98540	B808z00	Carcinoma in situ of liver or biliary system NOS	Liver and and biliopancreatic cancer
44166	B80z.00	Carcinoma in situ of other and unspecified digestive organs	Liver and and biliopancreatic cancer
16931	B80z000	Carcinoma in situ of pancreas	Liver and and biliopancreatic cancer
43490	Byu1100	[X]Other specified carcinomas of liver	Liver and and biliopancreatic cancer
108667	B1z1000	Angiosarcoma of spleen	Connective tissue cancer
72224	B1z1100	Fibrosarcoma of spleen	Connective tissue cancer
12539	B3...12	Sarcoma of bone and connective tissue	Connective tissue cancer
18314	B30..00	Malignant neoplasm of bone and articular cartilage	Connective tissue cancer

Medcode	Readcode	Description	Cancer type
5062	B30..11	Chondroma	Connective tissue cancer
29735	B30..12	Osteoma	Connective tissue cancer
59036	B300.00	Malignant neoplasm of bones of skull and face	Connective tissue cancer
53594	B300000	Malignant neoplasm of ethmoid bone	Connective tissue cancer
53599	B300100	Malignant neoplasm of frontal bone	Connective tissue cancer
59520	B300200	Malignant neoplasm of malar bone	Connective tissue cancer
95458	B300300	Malignant neoplasm of nasal bone	Connective tissue cancer
55953	B300400	Malignant neoplasm of occipital bone	Connective tissue cancer
50298	B300500	Malignant neoplasm of orbital bone	Connective tissue cancer
54747	B300600	Malignant neoplasm of parietal bone	Connective tissue cancer
55595	B300700	Malignant neoplasm of sphenoid bone	Connective tissue cancer
62104	B300800	Malignant neoplasm of temporal bone	Connective tissue cancer
50299	B300900	Malignant neoplasm of zygomatic bone	Connective tissue cancer
17475	B300A00	Malignant neoplasm of maxilla	Connective tissue cancer
96445	B300B00	Malignant neoplasm of turbinate	Connective tissue cancer
44452	B300C00	Malignant neoplasm of vomer	Connective tissue cancer
69146	B300z00	Malignant neoplasm of bones of skull and face NOS	Connective tissue cancer
33833	B301.00	Malignant neoplasm of mandible	Connective tissue cancer
16704	B302.00	Malignant neoplasm of vertebral column	Connective tissue cancer
46939	B302000	Malignant neoplasm of cervical vertebra	Connective tissue cancer
32372	B302100	Malignant neoplasm of thoracic vertebra	Connective tissue cancer
54691	B302200	Malignant neoplasm of lumbar vertebra	Connective tissue cancer
49701	B302z00	Malignant neoplasm of vertebral column NOS	Connective tissue cancer
27528	B303.00	Malignant neoplasm of ribs, sternum and clavicle	Connective tissue cancer
37842	B303000	Malignant neoplasm of rib	Connective tissue cancer
49491	B303100	Malignant neoplasm of sternum	Connective tissue cancer
66639	B303200	Malignant neoplasm of clavicle	Connective tissue cancer
60403	B303300	Malignant neoplasm of costal cartilage	Connective tissue cancer
67763	B303400	Malignant neoplasm of costo-vertebral joint	Connective tissue cancer
54493	B303500	Malignant neoplasm of xiphoid process	Connective tissue cancer

Medcode	Readcode	Description	Cancer type
51237	B303z00	Malignant neoplasm of rib, sternum and clavicle NOS	Connective tissue cancer
71810	B304.00	Malignant neoplasm of scapula and long bones of upper arm	Connective tissue cancer
49054	B304000	Malignant neoplasm of scapula	Connective tissue cancer
105797	B304100	Malignant neoplasm of acromion	Connective tissue cancer
61741	B304200	Malignant neoplasm of humerus	Connective tissue cancer
92371	B304300	Malignant neoplasm of radius	Connective tissue cancer
64848	B304400	Malignant neoplasm of ulna	Connective tissue cancer
65880	B304z00	Malig neop of scapula and long bones of upper arm NOS	Connective tissue cancer
73530	B305.00	Malignant neoplasm of hand bones	Connective tissue cancer
106069	B305.11	Malignant neoplasm of carpal bones	Connective tissue cancer
72464	B305.12	Malignant neoplasm of metacarpal bones	Connective tissue cancer
57988	B305000	Malignant neoplasm of carpal bone-scaphoid	Connective tissue cancer
69104	B305100	Malignant neoplasm of carpal bone-lunate	Connective tissue cancer
108638	B305A00	Malignant neoplasm of third metacarpal bone	Connective tissue cancer
94427	B305C00	Malignant neoplasm of fifth metacarpal bone	Connective tissue cancer
86812	B305D00	Malignant neoplasm of phalanges of hand	Connective tissue cancer
73556	B305z00	Malignant neoplasm of hand bones NOS	Connective tissue cancer
54631	B306.00	Malignant neoplasm of pelvic bones, sacrum and coccyx	Connective tissue cancer
44609	B306000	Malignant neoplasm of ilium	Connective tissue cancer
59223	B306100	Malignant neoplasm of ischium	Connective tissue cancer
51921	B306200	Malignant neoplasm of pubis	Connective tissue cancer
40966	B306300	Malignant neoplasm of sacral vertebra	Connective tissue cancer
66908	B306400	Malignant neoplasm of coccygeal vertebra	Connective tissue cancer
50152	B306500	Malignant sacral teratoma	Connective tissue cancer
38938	B306z00	Malignant neoplasm of pelvis, sacrum or coccyx NOS	Connective tissue cancer
68055	B307.00	Malignant neoplasm of long bones of leg	Connective tissue cancer
56513	B307000	Malignant neoplasm of femur	Connective tissue cancer
50402	B307100	Malignant neoplasm of fibula	Connective tissue cancer
40814	B307200	Malignant neoplasm of tibia	Connective tissue cancer
62630	B307z00	Malignant neoplasm of long bones of leg NOS	Connective tissue cancer

Medcode	Readcode	Description	Cancer type
105475	B308.00	Malignant neoplasm of short bones of leg	Connective tissue cancer
95182	B308100	Malignant neoplasm of talus	Connective tissue cancer
72212	B308200	Malignant neoplasm of calcaneum	Connective tissue cancer
34878	B308300	Malignant neoplasm of medial cuneiform	Connective tissue cancer
69927	B308800	Malignant neoplasm of first metatarsal bone	Connective tissue cancer
92382	B308B00	Malignant neoplasm of fourth metatarsal bone	Connective tissue cancer
58949	B308D00	Malignant neoplasm of phalanges of foot	Connective tissue cancer
103354	B308z00	Malignant neoplasm of short bones of leg NOS	Connective tissue cancer
67451	B30W.00	Malignant neoplasm/overlap lesion/bone+articulr cartilage	Connective tissue cancer
43614	B30X.00	Malignant neoplasm/bones+articular cartilage/limb,unspfd	Connective tissue cancer
16075	B30z.00	Malignant neoplasm of bone and articular cartilage NOS	Connective tissue cancer
19437	B30z000	Osteosarcoma	Connective tissue cancer
34451	B31..00	Malignant neoplasm of connective and other soft tissue	Connective tissue cancer
43475	B310.00	Malig neop of connective and soft tissue head, face and neck	Connective tissue cancer
59382	B310000	Malignant neoplasm of soft tissue of head	Connective tissue cancer
40014	B310100	Malignant neoplasm of soft tissue of face	Connective tissue cancer
48517	B310200	Malignant neoplasm of soft tissue of neck	Connective tissue cancer
60035	B310300	Malignant neoplasm of cartilage of ear	Connective tissue cancer
49463	B310400	Malignant neoplasm of tarsus of eyelid	Connective tissue cancer
108389	B310500	Malignant neoplasm soft tissues of cervical spine	Connective tissue cancer
73718	B310z00	Malig neop connective and soft tissue head, face, neck NOS	Connective tissue cancer
53989	B311.00	Malig neop connective and soft tissue upper limb/shoulder	Connective tissue cancer
50222	B311000	Malignant neoplasm of connective and soft tissue of shoulder	Connective tissue cancer
64345	B311100	Malignant neoplasm of connective and soft tissue, upper arm	Connective tissue cancer
57482	B311200	Malignant neoplasm of connective and soft tissue of fore-arm	Connective tissue cancer
19321	B311300	Malignant neoplasm of connective and soft tissue of hand	Connective tissue cancer
91586	B311400	Malignant neoplasm of connective and soft tissue of finger	Connective tissue cancer
63988	B311500	Malignant neoplasm of connective and soft tissue of thumb	Connective tissue cancer
104913	B311z00	Malig neop connective soft tissue upper limb/shoulder NOS	Connective tissue cancer
66088	B312.00	Malig neop of connective and soft tissue of hip and leg	Connective tissue cancer

Medcode	Readcode	Description	Cancer type
102949	B312000	Malignant neoplasm of connective and soft tissue of hip	Connective tissue cancer
44805	B312100	Malig neop of connective and soft tissue thigh and upper leg	Connective tissue cancer
54965	B312200	Malig neop connective and soft tissue of popliteal space	Connective tissue cancer
30542	B312300	Malig neop of connective and soft tissue of lower leg	Connective tissue cancer
54222	B312400	Malignant neoplasm of connective and soft tissue of foot	Connective tissue cancer
99572	B312500	Malignant neoplasm of connective and soft tissue of toe	Connective tissue cancer
90546	B312z00	Malig neop connective and soft tissue hip and leg NOS	Connective tissue cancer
22290	B313.00	Malignant neoplasm of connective and soft tissue of thorax	Connective tissue cancer
29160	B313000	Malignant neoplasm of connective and soft tissue of axilla	Connective tissue cancer
54186	B313100	Malignant neoplasm of diaphragm	Connective tissue cancer
72522	B313200	Malignant neoplasm of great vessels	Connective tissue cancer
104139	B313300	Malig neoplasm of connective and soft tissues of thor spine	Connective tissue cancer
98408	B313z00	Malig neop of connective and soft tissue of thorax NOS	Connective tissue cancer
45071	B314.00	Malignant neoplasm of connective and soft tissue of abdomen	Connective tissue cancer
66488	B314000	Malig neop of connective and soft tissue of abdominal wall	Connective tissue cancer
94272	B314100	Malig neoplasm of connective and soft tissues of lumb spine	Connective tissue cancer
60247	B314z00	Malig neop of connective and soft tissue of abdomen NOS	Connective tissue cancer
51965	B315.00	Malignant neoplasm of connective and soft tissue of pelvis	Connective tissue cancer
70463	B315000	Malignant neoplasm of connective and soft tissue of buttock	Connective tissue cancer
67324	B315100	Malig neop of connective and soft tissue of inguinal region	Connective tissue cancer
59152	B315200	Malignant neoplasm of connective and soft tissue of perineum	Connective tissue cancer
110192	B315300	Malig neopl of connective and soft tissue-sacrum or coccyx	Connective tissue cancer
58836	B315z00	Malig neop of connective and soft tissue of pelvis NOS	Connective tissue cancer
57471	B316.00	Malig neop of connective and soft tissue trunk unspecified	Connective tissue cancer
65233	B31y.00	Malig neop connective and soft tissue other specified site	Connective tissue cancer
15182	B31z.00	Malignant neoplasm of connective and soft tissue, site NOS	Connective tissue cancer
104128	B31z000	Kaposi's sarcoma of soft tissue	Connective tissue cancer
27931	B33z000	Kaposi's sarcoma of skin	Connective tissue cancer
40749	Byu3.00	[X]Malignant neoplasm of bone and articular cartilage	Connective tissue cancer
73296	Byu3100	[X]Malignant neoplasm/bones+articular cartilage/limb,unspfd	Connective tissue cancer

Medcode	Readcode	Description	Cancer type
63300	Byu3200	[X]Malignant neoplasm/overlap lesion/bone+articulr cartilage	Connective tissue cancer
43151	Byu3300	[X]Malignant neoplasm/bone+articular cartilage, unspecified	Connective tissue cancer
4632	B33..00	Other malignant neoplasm of skin	Non melanoma skin cancer
876	B33..11	Basal cell carcinoma	Non melanoma skin cancer
5034	B33..12	Epithelioma	Non melanoma skin cancer
1940	B33..13	Rodent ulcer	Non melanoma skin cancer
37016	B33..14	Malignant neoplasm of sebaceous gland	Non melanoma skin cancer
40443	B33..15	Malignant neoplasm of sweat gland	Non melanoma skin cancer
3445	B33..16	Epithelioma basal cell	Non melanoma skin cancer
18245	B330.00	Malignant neoplasm of skin of lip	Non melanoma skin cancer
43087	B331.00	Malignant neoplasm of eyelid including canthus	Non melanoma skin cancer
36731	B331000	Malignant neoplasm of canthus	Non melanoma skin cancer
55550	B331100	Malignant neoplasm of upper eyelid	Non melanoma skin cancer
41958	B331200	Malignant neoplasm of lower eyelid	Non melanoma skin cancer
53515	B332.00	Malignant neoplasm skin of ear and external auricular canal	Non melanoma skin cancer
33997	B332000	Malignant neoplasm of skin of auricle (ear)	Non melanoma skin cancer
62080	B332100	Malignant neoplasm of skin of external auditory meatus	Non melanoma skin cancer
33271	B332200	Malignant neoplasm of pinna NEC	Non melanoma skin cancer
62399	B332z00	Malig neop skin of ear and external auricular canal NOS	Non melanoma skin cancer
27370	B333.00	Malignant neoplasm skin of other and unspecified parts face	Non melanoma skin cancer
30645	B333000	Malignant neoplasm of skin of cheek, external	Non melanoma skin cancer
49403	B333100	Malignant neoplasm of skin of chin	Non melanoma skin cancer
55670	B333200	Malignant neoplasm of skin of eyebrow	Non melanoma skin cancer
30576	B333300	Malignant neoplasm of skin of forehead	Non melanoma skin cancer
16202	B333400	Malignant neoplasm of skin of nose (external)	Non melanoma skin cancer
21327	B333500	Malignant neoplasm of skin of temple	Non melanoma skin cancer
46008	B333z00	Malignant neoplasm skin other and unspec part of face NOS	Non melanoma skin cancer
54234	B334.00	Malignant neoplasm of scalp and skin of neck	Non melanoma skin cancer
37165	B334000	Malignant neoplasm of scalp	Non melanoma skin cancer
43619	B334100	Malignant neoplasm of skin of neck	Non melanoma skin cancer

Medcode	Readcode	Description	Cancer type
73760	B334z00	Malignant neoplasm of scalp or skin of neck NOS	Non melanoma skin cancer
57446	B335.00	Malignant neoplasm of skin of trunk, excluding scrotum	Non melanoma skin cancer
70380	B335000	Malignant neoplasm of skin of axillary fold	Non melanoma skin cancer
37969	B335100	Malignant neoplasm of skin of chest, excluding breast	Non melanoma skin cancer
30543	B335200	Malignant neoplasm of skin of breast	Non melanoma skin cancer
18618	B335300	Malignant neoplasm of skin of abdominal wall	Non melanoma skin cancer
67748	B335400	Malignant neoplasm of skin of umbilicus	Non melanoma skin cancer
66319	B335500	Malignant neoplasm of skin of groin	Non melanoma skin cancer
46458	B335600	Malignant neoplasm of skin of perineum	Non melanoma skin cancer
45077	B335700	Malignant neoplasm of skin of back	Non melanoma skin cancer
62305	B335800	Malignant neoplasm of skin of buttock	Non melanoma skin cancer
23480	B335900	Malignant neoplasm of perianal skin	Non melanoma skin cancer
66447	B335A00	Malignant neoplasm of skin of scapular region	Non melanoma skin cancer
15868	B335z00	Malignant neoplasm of skin of trunk, excluding scrotum, NOS	Non melanoma skin cancer
30747	B336.00	Malignant neoplasm of skin of upper limb and shoulder	Non melanoma skin cancer
43122	B336000	Malignant neoplasm of skin of shoulder	Non melanoma skin cancer
42707	B336100	Malignant neoplasm of skin of upper arm	Non melanoma skin cancer
30577	B336200	Malignant neoplasm of skin of fore-arm	Non melanoma skin cancer
54352	B336300	Malignant neoplasm of skin of hand	Non melanoma skin cancer
25245	B336400	Malignant neoplasm of skin of finger	Non melanoma skin cancer
64406	B336500	Malignant neoplasm of skin of thumb	Non melanoma skin cancer
60526	B336z00	Malignant neoplasm of skin of upper limb or shoulder NOS	Non melanoma skin cancer
57442	B337.00	Malignant neoplasm of skin of lower limb and hip	Non melanoma skin cancer
70988	B337000	Malignant neoplasm of skin of hip	Non melanoma skin cancer
58601	B337100	Malignant neoplasm of skin of thigh	Non melanoma skin cancer
56954	B337200	Malignant neoplasm of skin of knee	Non melanoma skin cancer
68197	B337300	Malignant neoplasm of skin of popliteal fossa area	Non melanoma skin cancer
33682	B337400	Malignant neoplasm of skin of lower leg	Non melanoma skin cancer
64270	B337500	Malignant neoplasm of skin of ankle	Non melanoma skin cancer
104025	B337600	Malignant neoplasm of skin of heel	Non melanoma skin cancer

Medcode	Readcode	Description	Cancer type
70587	B337700	Malignant neoplasm of skin of foot	Non melanoma skin cancer
65782	B337800	Malignant neoplasm of skin of toe	Non melanoma skin cancer
67914	B337900	Malignant neoplasm of skin of great toe	Non melanoma skin cancer
61194	B337z00	Malignant neoplasm of skin of lower limb or hip NOS	Non melanoma skin cancer
93352	B338.00	Squamous cell carcinoma of skin	Non melanoma skin cancer
24375	B339.00	Dermatofibrosarcoma protuberans	Non melanoma skin cancer
42429	B33X.00	Malignant neoplasm overlapping lesion of skin	Non melanoma skin cancer
18354	B33y.00	Malignant neoplasm of other specified skin sites	Non melanoma skin cancer
2492	B33z.00	Malignant neoplasm of skin NOS	Non melanoma skin cancer
93490	B33z.11	Squamous cell carcinoma of skin NOS	Non melanoma skin cancer
12084	B82..00	Carcinoma in situ of skin	Non melanoma skin cancer
63957	B820.00	Carcinoma in situ of skin of lip	Non melanoma skin cancer
57550	B821.00	Carcinoma in situ of skin of eyelid including canthus	Non melanoma skin cancer
50189	B822.00	Carcinoma in situ skin of ear and external auricular canal	Non melanoma skin cancer
32249	B822.11	Carcinoma in situ of ear	Non melanoma skin cancer
59614	B822000	Carcinoma in situ of skin of auricle	Non melanoma skin cancer
70295	B822z00	Carcinoma in situ skin of ear/external auricular canal NOS	Non melanoma skin cancer
49254	B823.00	Carcinoma in situ of skin of other parts of face	Non melanoma skin cancer
47789	B823000	Carcinoma in situ of skin of forehead skin	Non melanoma skin cancer
69720	B823100	Carcinoma in situ of skin of eyebrow	Non melanoma skin cancer
61103	B823300	Carcinoma in situ of skin of cheek	Non melanoma skin cancer
3135	B823400	Carcinoma in situ of skin of nose	Non melanoma skin cancer
31511	B823500	Carcinoma in situ of skin of temple	Non melanoma skin cancer
65222	B823600	Carcinoma in situ of skin of jaw	Non melanoma skin cancer
110614	B823z00	Carcinoma in situ of skin of other parts of face NOS	Non melanoma skin cancer
69345	B824.00	Carcinoma in situ of scalp and skin of neck	Non melanoma skin cancer
19665	B824000	Carcinoma in situ of scalp	Non melanoma skin cancer
54140	B824100	Carcinoma in situ of skin of neck	Non melanoma skin cancer
52328	B825.00	Carcinoma in situ of skin of trunk, excluding scrotum	Non melanoma skin cancer
8647	B825000	Carcinoma in situ of skin of breast	Non melanoma skin cancer

Medcode	Readcode	Description	Cancer type
62939	B825100	Carcinoma in situ of skin of chest wall NOS	Non melanoma skin cancer
39390	B825200	Carcinoma in situ of skin of axilla	Non melanoma skin cancer
38032	B825300	Carcinoma in situ of skin of back	Non melanoma skin cancer
42212	B825400	Carcinoma in situ of skin of abdominal wall	Non melanoma skin cancer
57358	B825500	Carcinoma in situ of skin of groin	Non melanoma skin cancer
38777	B825600	Carcinoma in situ of skin of perineum	Non melanoma skin cancer
61321	B825700	Carcinoma in situ of skin of buttock	Non melanoma skin cancer
56374	B825800	Carcinoma in situ of perianal skin	Non melanoma skin cancer
60563	B825z00	Carcinoma in situ of skin of trunk NOS	Non melanoma skin cancer
46568	B826.00	Carcinoma in situ of skin of upper limb and shoulder	Non melanoma skin cancer
56554	B826000	Carcinoma in situ of skin of shoulder	Non melanoma skin cancer
57284	B826100	Carcinoma in situ of skin of upper arm	Non melanoma skin cancer
54790	B826200	Carcinoma in situ of skin of lower arm	Non melanoma skin cancer
49358	B826300	Carcinoma in situ of skin of hand	Non melanoma skin cancer
90339	B826z00	Carcinoma in situ of skin of upper limb or shoulder NOS	Non melanoma skin cancer
14815	B827.00	Carcinoma in situ of skin of lower limb and hip	Non melanoma skin cancer
708	B827.11	Carcinoma in situ of skin of leg	Non melanoma skin cancer
71655	B827000	Carcinoma in situ of skin of hip	Non melanoma skin cancer
46469	B827100	Carcinoma in situ of skin of thigh	Non melanoma skin cancer
69601	B827200	Carcinoma in situ of skin of knee	Non melanoma skin cancer
27542	B827300	Carcinoma in situ of skin of lower leg	Non melanoma skin cancer
67755	B827400	Carcinoma in situ of skin of foot	Non melanoma skin cancer
64630	B827z00	Carcinoma in situ of skin of lower limb or hip NOS	Non melanoma skin cancer
865	B32..00	Malignant melanoma of skin	Melanoma
70637	B320.00	Malignant melanoma of lip	Melanoma
54632	B321.00	Malignant melanoma of eyelid including canthus	Melanoma
57260	B322.00	Malignant melanoma of ear and external auricular canal	Melanoma
59061	B322000	Malignant melanoma of auricle (ear)	Melanoma
102145	B322100	Malignant melanoma of external auditory meatus	Melanoma
73744	B322z00	Malignant melanoma of ear and external auricular canal NOS	Melanoma
47252	B323.00	Malignant melanoma of other and unspecified parts of face	Melanoma
41278	B323000	Malignant melanoma of external surface of cheek	Melanoma
71136	B323100	Malignant melanoma of chin	Melanoma
47094	B323200	Malignant melanoma of eyebrow	Melanoma

Medcode	Readcode	Description	Cancer type
68133	B323300	Malignant melanoma of forehead	Melanoma
45139	B323400	Malignant melanoma of external surface of nose	Melanoma
58958	B323500	Malignant melanoma of temple	Melanoma
67806	B323z00	Malignant melanoma of face NOS	Melanoma
65625	B324.00	Malignant melanoma of scalp and neck	Melanoma
55881	B324000	Malignant melanoma of scalp	Melanoma
45306	B324100	Malignant melanoma of neck	Melanoma
99257	B324z00	Malignant melanoma of scalp and neck NOS	Melanoma
38689	B325.00	Malignant melanoma of trunk (excluding scrotum)	Melanoma
49814	B325000	Malignant melanoma of axilla	Melanoma
32768	B325100	Malignant melanoma of breast	Melanoma
53629	B325200	Malignant melanoma of buttock	Melanoma
34259	B325300	Malignant melanoma of groin	Melanoma
109002	B325400	Malignant melanoma of perianal skin	Melanoma
95629	B325500	Malignant melanoma of perineum	Melanoma
43715	B325600	Malignant melanoma of umbilicus	Melanoma
43463	B325700	Malignant melanoma of back	Melanoma
51209	B325800	Malignant melanoma of chest wall	Melanoma
45760	B325z00	Malignant melanoma of trunk, excluding scrotum, NOS	Melanoma
65164	B326.00	Malignant melanoma of upper limb and shoulder	Melanoma
50505	B326000	Malignant melanoma of shoulder	Melanoma
54685	B326100	Malignant melanoma of upper arm	Melanoma
45755	B326200	Malignant melanoma of fore-arm	Melanoma
62475	B326300	Malignant melanoma of hand	Melanoma
25602	B326400	Malignant melanoma of finger	Melanoma
63997	B326500	Malignant melanoma of thumb	Melanoma
55292	B326z00	Malignant melanoma of upper limb or shoulder NOS	Melanoma
46255	B327.00	Malignant melanoma of lower limb and hip	Melanoma
73536	B327000	Malignant melanoma of hip	Melanoma
51873	B327100	Malignant melanoma of thigh	Melanoma
54305	B327200	Malignant melanoma of knee	Melanoma
39878	B327300	Malignant melanoma of popliteal fossa area	Melanoma
37872	B327400	Malignant melanoma of lower leg	Melanoma
42714	B327500	Malignant melanoma of ankle	Melanoma
61246	B327600	Malignant melanoma of heel	Melanoma
41490	B327700	Malignant melanoma of foot	Melanoma
36899	B327800	Malignant melanoma of toe	Melanoma
53369	B327900	Malignant melanoma of great toe	Melanoma
64327	B327z00	Malignant melanoma of lower limb or hip NOS	Melanoma
42153	B32y.00	Malignant melanoma of other specified skin site	Melanoma
96585	B32y000	Overlapping malignant melanoma of skin	Melanoma
28556	B32z.00	Malignant melanoma of skin NOS	Melanoma
19686	B828.00	Melanoma in situ of skin	Melanoma
46536	B828000	Melanoma in situ of lip	Melanoma
37108	B828100	Melanoma in situ of eyelid, including canthus	Melanoma
72032	B828200	Melanoma in situ of ear and external auricular canal	Melanoma

Medcode	Readcode	Description	Cancer type
97858	B828300	Melanoma in situ of scalp and neck	Melanoma
59768	B828400	Melanoma in situ of trunk	Melanoma
56694	B828500	Melanoma in situ of upper limb, including shoulder	Melanoma
47850	B828600	Melanoma in situ of lower limb, including hip	Melanoma
49572	B828700	Melanoma in situ of scalp	Melanoma
52332	B828800	Melanoma in situ of back of hand	Melanoma
71044	B828900	Melanoma in situ of back	Melanoma
54246	B828W00	Melanoma in situ, unspecified	Melanoma
61989	B828X00	Melanoma in situ of other and unspecified parts of face	Melanoma
56925	Byu4000	[X]Malignant melanoma of other+unspecified parts of face	Melanoma
19444	Byu4100	[X]Malignant melanoma of skin, unspecified	Melanoma
97628	ByuF600	[X]Melanoma in situ of other sites	Melanoma
73261	ByuFF00	[X]Melanoma in situ, unspecified	Melanoma
779	B49..00	Malignant neoplasm of urinary bladder	Urinary tract cancer
38862	B490.00	Malignant neoplasm of trigone of urinary bladder	Urinary tract cancer
44996	B491.00	Malignant neoplasm of dome of urinary bladder	Urinary tract cancer
35963	B492.00	Malignant neoplasm of lateral wall of urinary bladder	Urinary tract cancer
19162	B493.00	Malignant neoplasm of anterior wall of urinary bladder	Urinary tract cancer
42012	B494.00	Malignant neoplasm of posterior wall of urinary bladder	Urinary tract cancer
41571	B495.00	Malignant neoplasm of bladder neck	Urinary tract cancer
28241	B496.00	Malignant neoplasm of ureteric orifice	Urinary tract cancer
42023	B497.00	Malignant neoplasm of urachus	Urinary tract cancer
105388	B498.00	Local recurrence of malignant tumour of urinary bladder	Urinary tract cancer
36949	B49y.00	Malignant neoplasm of other site of urinary bladder	Urinary tract cancer
47801	B49y000	Malignant neoplasm, overlapping lesion of bladder	Urinary tract cancer
31102	B49z.00	Malignant neoplasm of urinary bladder NOS	Urinary tract cancer
13559	B4A..00	Malig neop of kidney and other unspecified urinary organs	Urinary tract cancer
18712	B4A..11	Renal malignant neoplasm	Urinary tract cancer
1599	B4A0.00	Malignant neoplasm of kidney parenchyma	Urinary tract cancer
7978	B4A0000	Hypernephroma	Urinary tract cancer
12389	B4A1.00	Malignant neoplasm of renal pelvis	Urinary tract cancer
27540	B4A1000	Malignant neoplasm of renal calyces	Urinary tract cancer
101608	B4A1100	Malignant neoplasm of ureteropelvic junction	Urinary tract cancer
54184	B4A1z00	Malignant neoplasm of renal pelvis NOS	Urinary tract cancer
15223	B4A2.00	Malignant neoplasm of ureter	Urinary tract cancer
15644	B4A3.00	Malignant neoplasm of urethra	Urinary tract cancer
72174	B4A4.00	Malignant neoplasm of paraurethral glands	Urinary tract cancer
44884	B4Ay.00	Malignant neoplasm of other urinary organs	Urinary tract cancer
59286	B4Ay000	Malignant neoplasm of overlapping lesion of urinary organs	Urinary tract cancer
29462	B4Az.00	Malignant neoplasm of kidney or urinary organs NOS	Urinary tract cancer
38931	B4y..00	Malignant neoplasm of genitourinary organ OS	Urinary tract cancer
52594	B4z..00	Malignant neoplasm of genitourinary organ NOS	Urinary tract cancer
35113	Byu9.00	[X]Malignant neoplasm of urinary tract	Urinary tract cancer
45260	Byu9000	[X]Malignant neoplasm of urinary organ, unspecified	Urinary tract cancer
3968	B34..00	Malignant neoplasm of female breast	Breast cancer
348	B34..11	Ca female breast	Breast cancer

Medcode	Readcode	Description	Cancer type
26853	B340.00	Malignant neoplasm of nipple and areola of female breast	Breast cancer
23380	B340000	Malignant neoplasm of nipple of female breast	Breast cancer
64686	B340100	Malignant neoplasm of areola of female breast	Breast cancer
59831	B340z00	Malignant neoplasm of nipple or areola of female breast NOS	Breast cancer
31546	B341.00	Malignant neoplasm of central part of female breast	Breast cancer
29826	B342.00	Malignant neoplasm of upper-inner quadrant of female breast	Breast cancer
45222	B343.00	Malignant neoplasm of lower-inner quadrant of female breast	Breast cancer
23399	B344.00	Malignant neoplasm of upper-outer quadrant of female breast	Breast cancer
42070	B345.00	Malignant neoplasm of lower-outer quadrant of female breast	Breast cancer
20685	B346.00	Malignant neoplasm of axillary tail of female breast	Breast cancer
49148	B347.00	Malignant neoplasm, overlapping lesion of breast	Breast cancer
56715	B34y.00	Malignant neoplasm of other site of female breast	Breast cancer
95057	B34y000	Malignant neoplasm of ectopic site of female breast	Breast cancer
38475	B34yz00	Malignant neoplasm of other site of female breast NOS	Breast cancer
9470	B34z.00	Malignant neoplasm of female breast NOS	Breast cancer
19423	B35..00	Malignant neoplasm of male breast	Breast cancer
54494	B350.00	Malignant neoplasm of nipple and areola of male breast	Breast cancer
68480	B350000	Malignant neoplasm of nipple of male breast	Breast cancer
67884	B350100	Malignant neoplasm of areola of male breast	Breast cancer
54202	B35z.00	Malignant neoplasm of other site of male breast	Breast cancer
95323	B35z000	Malignant neoplasm of ectopic site of male breast	Breast cancer
48809	B35zz00	Malignant neoplasm of male breast NOS	Breast cancer
105488	B36..00	Local recurrence of malignant tumour of breast	Breast cancer
7833	B830.00	Carcinoma in situ of breast	Breast cancer
10387	B830000	Lobular carcinoma in situ of breast	Breast cancer
18694	B830100	Intraductal carcinoma in situ of breast	Breast cancer
12499	Byu6.00	[X]Malignant neoplasm of breast	Breast cancer
53803	ByuFG00	[X]Other carcinoma in situ of breast	Breast cancer
2744	B40..00	Malignant neoplasm of uterus, part unspecified	Gynecologic cancer
2747	B41..00	Malignant neoplasm of cervix uteri	Gynecologic cancer
3230	B41..11	Cervical carcinoma (uterus)	Gynecologic cancer
48820	B410.00	Malignant neoplasm of endocervix	Gynecologic cancer
57235	B410000	Malignant neoplasm of endocervical canal	Gynecologic cancer
53103	B410100	Malignant neoplasm of endocervical gland	Gynecologic cancer
50285	B410z00	Malignant neoplasm of endocervix NOS	Gynecologic cancer
50297	B411.00	Malignant neoplasm of exocervix	Gynecologic cancer
58094	B412.00	Malignant neoplasm, overlapping lesion of cervix uteri	Gynecologic cancer
32955	B41y.00	Malignant neoplasm of other site of cervix	Gynecologic cancer
95505	B41y000	Malignant neoplasm of cervical stump	Gynecologic cancer
57719	B41y100	Malignant neoplasm of squamocolumnar junction of cervix	Gynecologic cancer
43435	B41yz00	Malignant neoplasm of other site of cervix NOS	Gynecologic cancer
28311	B41z.00	Malignant neoplasm of cervix uteri NOS	Gynecologic cancer
93762	B42..00	Malignant neoplasm of placenta	Gynecologic cancer
28003	B420.00	Choriocarcinoma	Gynecologic cancer
7046	B43..00	Malignant neoplasm of body of uterus	Gynecologic cancer
3213	B430.00	Malignant neoplasm of corpus uteri, excluding isthmus	Gynecologic cancer

Medcode	Readcode	Description	Cancer type
72723	B430000	Malignant neoplasm of cornu of corpus uteri	Gynecologic cancer
68155	B430100	Malignant neoplasm of fundus of corpus uteri	Gynecologic cancer
2890	B430200	Malignant neoplasm of endometrium of corpus uteri	Gynecologic cancer
49400	B430211	Malignant neoplasm of endometrium	Gynecologic cancer
45793	B430300	Malignant neoplasm of myometrium of corpus uteri	Gynecologic cancer
45490	B430z00	Malignant neoplasm of corpus uteri NOS	Gynecologic cancer
43940	B431.00	Malignant neoplasm of isthmus of uterine body	Gynecologic cancer
59097	B431000	Malignant neoplasm of lower uterine segment	Gynecologic cancer
70729	B431z00	Malignant neoplasm of isthmus of uterine body NOS	Gynecologic cancer
16967	B432.00	Malignant neoplasm of overlapping lesion of corpus uteri	Gynecologic cancer
31608	B43y.00	Malignant neoplasm of other site of uterine body	Gynecologic cancer
33617	B43z.00	Malignant neoplasm of body of uterus NOS	Gynecologic cancer
19141	B44..00	Malignant neoplasm of ovary and other uterine adnexa	Gynecologic cancer
7805	B440.00	Malignant neoplasm of ovary	Gynecologic cancer
1986	B440.11	Cancer of ovary	Gynecologic cancer
49828	B441.00	Malignant neoplasm of fallopian tube	Gynecologic cancer
101778	B442.00	Malignant neoplasm of broad ligament	Gynecologic cancer
46153	B443.00	Malignant neoplasm of parametrium	Gynecologic cancer
97996	B44y.00	Malignant neoplasm of other site of uterine adnexa	Gynecologic cancer
65106	B44z.00	Malignant neoplasm of uterine adnexa NOS	Gynecologic cancer
4555	B45..00	Malig neop of other and unspecified female genital organs	Gynecologic cancer
37328	B450.00	Malignant neoplasm of vagina	Gynecologic cancer
10698	B450100	Malignant neoplasm of vaginal vault	Gynecologic cancer
60772	B450z00	Malignant neoplasm of vagina NOS	Gynecologic cancer
43761	B451.00	Malignant neoplasm of labia majora	Gynecologic cancer
47899	B451000	Malignant neoplasm of greater vestibular (Bartholin's) gland	Gynecologic cancer
59362	B451z00	Malignant neoplasm of labia majora NOS	Gynecologic cancer
58061	B452.00	Malignant neoplasm of labia minora	Gynecologic cancer
53910	B453.00	Malignant neoplasm of clitoris	Gynecologic cancer
4554	B454.00	Malignant neoplasm of vulva unspecified	Gynecologic cancer
11991	B454.11	Primary vulval cancer	Gynecologic cancer
26454	B45X.00	Malignant neoplasm/overlapping lesion/feml genital organs	Gynecologic cancer
95421	B45y.00	Malignant neoplasm of other specified female genital organ	Gynecologic cancer
27617	B45y000	Malignant neoplasm of overlapping lesion of vulva	Gynecologic cancer
20166	B45z.00	Malignant neoplasm of female genital organ NOS	Gynecologic cancer
3279	B831.00	Carcinoma in situ of cervix uteri	Gynecologic cancer
4087	B831.11	CIN III-carcinoma in situ of cervix	Gynecologic cancer
5295	B831.12	Cervical intraepithelial neoplasia	Gynecologic cancer
21886	B831.13	Cervical intraepithelial neoplasia grade III	Gynecologic cancer
24228	B831000	Carcinoma in situ of endocervix	Gynecologic cancer
50126	B831100	Carcinoma in situ of exocervix	Gynecologic cancer
29898	B832.00	Carcinoma in situ of other and unspecified parts of uterus	Gynecologic cancer
61803	B832.11	Carcinoma in situ of body of uterus	Gynecologic cancer
7904	B832000	Carcinoma in situ of endometrium	Gynecologic cancer
44915	B833.00	Carcinoma in situ other and unspecified female genital organ	Gynecologic cancer
17137	B833000	Carcinoma in situ of ovary	Gynecologic cancer

Medcode	Readcode	Description	Cancer type
59499	B833100	Carcinoma in situ of fallopian tube	Gynecologic cancer
34946	B833200	Carcinoma in situ of vagina	Gynecologic cancer
12119	B833300	Carcinoma in situ of vulva	Gynecologic cancer
3281	B833311	Vulval intraepithelial neoplasia	Gynecologic cancer
40598	Byu7.00	[X]Malignant neoplasm of female genital organs	Gynecologic cancer
64497	Byu7000	[X]Malignant neoplasm of uterine adnexa, unspecified	Gynecologic cancer
57756	Byu7100	[X]Malignant neoplasm/other specified female genital organs	Gynecologic cancer
55588	Byu7300	[X]Malignant neoplasm of female genital organ, unspecified	Gynecologic cancer
72695	ByuFA00	[X]Carcinoma in situ of other parts of cervix	Gynecologic cancer
15148	B47..00	Malignant neoplasm of testis	Male genital cancer
64602	B470.00	Malignant neoplasm of undescended testis	Male genital cancer
7740	B470200	Seminoma of undescended testis	Male genital cancer
36325	B470300	Teratoma of undescended testis	Male genital cancer
96429	B470z00	Malignant neoplasm of undescended testis NOS	Male genital cancer
19475	B471.00	Malignant neoplasm of descended testis	Male genital cancer
21786	B471000	Seminoma of descended testis	Male genital cancer
9476	B471100	Teratoma of descended testis	Male genital cancer
91509	B471z00	Malignant neoplasm of descended testis NOS	Male genital cancer
38510	B47z.00	Malignant neoplasm of testis NOS	Male genital cancer
2961	B47z.11	Seminoma of testis	Male genital cancer
15989	B47z.12	Teratoma of testis	Male genital cancer
3541	B48..00	Malignant neoplasm of penis and other male genital organs	Male genital cancer
50681	B480.00	Malignant neoplasm of prepuce (foreskin)	Male genital cancer
17841	B481.00	Malignant neoplasm of glans penis	Male genital cancer
48743	B482.00	Malignant neoplasm of body of penis	Male genital cancer
43392	B483.00	Malignant neoplasm of penis, part unspecified	Male genital cancer
72127	B484.00	Malignant neoplasm of epididymis	Male genital cancer
63331	B485.00	Malignant neoplasm of spermatic cord	Male genital cancer
47767	B486.00	Malignant neoplasm of scrotum	Male genital cancer
52570	B487.00	Malignant neoplasm, overlapping lesion of penis	Male genital cancer
67949	B48y.00	Malignant neoplasm of other male genital organ	Male genital cancer
68161	B48y000	Malignant neoplasm of seminal vesicle	Male genital cancer
47668	B48y100	Malignant neoplasm of tunica vaginalis	Male genital cancer
68824	B48y200	Malignant neoplasm, overlapping lesion male genital orgs	Male genital cancer
92329	B48yz00	Malignant neoplasm of other male genital organ NOS	Male genital cancer
63224	B48z.00	Malignant neoplasm of penis and other male genital organ NOS	Male genital cancer
27311	B835.00	Carcinoma in situ of penis	Male genital cancer
107958	B836.00	Carcinoma in situ other and unspecified male genital organs	Male genital cancer
8177	B836000	Carcinoma in situ of testis	Male genital cancer
58879	B836300	Carcinoma in situ of scrotum	Male genital cancer
109831	B836z00	Carcinoma in situ of male genital organs NOS	Male genital cancer
7187	B837.00	Carcinoma in situ of bladder	Male genital cancer
68358	B83z.00	Carcinoma in situ of urinary organs NOS	Male genital cancer
40671	Byu8.00	[X]Malignant neoplasm of male genital organs	Male genital cancer
57191	Byu8000	[X]Malignant neoplasm/other specified male genital organs	Male genital cancer
45262	Byu8200	[X]Malignant neoplasm of male genital organ, unspecified	Male genital cancer

Medcode	Readcode	Description	Cancer type
106003	ByuFC00	[X]Carcinoma in situ of oth+unspecified male genital organs	Male genital cancer
102314	1427000	H/O: prostate cancer	Prostate cancer
780	B46..00	Malignant neoplasm of prostate	Prostate cancer
6328	B834.00	Carcinoma in situ of prostate	Prostate cancer
54599	B834000	High grade prostatic intraepithelial neoplasia	Prostate cancer
105236	B834100	Prostatic intraepithelial neoplasia	Prostate cancer
37306	ZV10415	[V]Personal history of malignant neoplasm of prostate	Prostate cancer
2462	B61..00	Hodgkin's disease	Any cancer
104291	B61..11	Hodgkin lymphoma	Any cancer
65489	B610.00	Hodgkin's paragranuloma	Any cancer
100423	B610100	Hodgkin's paragranuloma of lymph nodes of head, face, neck	Any cancer
98840	B610300	Hodgkin's paragranuloma of intra-abdominal lymph nodes	Any cancer
44196	B611.00	Hodgkin's granuloma	Any cancer
98909	B611100	Hodgkin's granuloma of lymph nodes of head, face and neck	Any cancer
64036	B612.00	Hodgkin's sarcoma	Any cancer
68039	B612400	Hodgkin's sarcoma of lymph nodes of axilla and upper limb	Any cancer
38939	B613.00	Hodgkin's disease, lymphocytic-histiocytic predominance	Any cancer
71142	B613000	Hodgkin's, lymphocytic-histiocytic predominance unspec site	Any cancer
68330	B613100	Hodgkin's, lymphocytic-histiocytic pred of head, face, neck	Any cancer
92245	B613200	Hodgkin's, lymphocytic-histiocytic pred intrathoracic nodes	Any cancer
73532	B613300	Hodgkin's, lymphocytic-histiocytic pred intra-abdominal node	Any cancer
93951	B613500	Hodgkin's, lymphocytic-histiocytic pred inguinal and leg	Any cancer
95338	B613600	Hodgkin's, lymphocytic-histiocytic pred intrapelvic nodes	Any cancer
106911	B613700	Hodgkin's, lymphocytic-histiocytic predominance of spleen	Any cancer
104743	B613800	Hodgkin's, lymphocytic-histiocytic pred of multiple sites	Any cancer
29876	B613z00	Hodgkin's, lymphocytic-histiocytic predominance NOS	Any cancer
29178	B614.00	Hodgkin's disease, nodular sclerosis	Any cancer
57225	B614000	Hodgkin's disease, nodular sclerosis of unspecified site	Any cancer
55303	B614100	Hodgkin's nodular sclerosis of head, face and neck	Any cancer
67506	B614200	Hodgkin's nodular sclerosis of intrathoracic lymph nodes	Any cancer
61149	B614300	Hodgkin's nodular sclerosis of intra-abdominal lymph nodes	Any cancer
65483	B614400	Hodgkin's nodular sclerosis of lymph nodes of axilla and arm	Any cancer
105472	B614700	Hodgkin's disease, nodular sclerosis of spleen	Any cancer
19140	B614800	Hodgkin's nodular sclerosis of lymph nodes of multiple sites	Any cancer
63054	B614z00	Hodgkin's disease, nodular sclerosis NOS	Any cancer
49605	B615.00	Hodgkin's disease, mixed cellularity	Any cancer
97863	B615000	Hodgkin's disease, mixed cellularity of unspecified site	Any cancer
94407	B615100	Hodgkin's mixed cellularity of lymph nodes head, face, neck	Any cancer
58684	B615200	Hodgkin's mixed cellularity of intrathoracic lymph nodes	Any cancer
108886	B615500	Hodgkin's mixed cellularity of lymph nodes inguinal and leg	Any cancer
94005	B615z00	Hodgkin's disease, mixed cellularity NOS	Any cancer
67703	B616.00	Hodgkin's disease, lymphocytic depletion	Any cancer
95049	B616000	Hodgkin's lymphocytic depletion of unspecified site	Any cancer
63625	B616400	Hodgkin's lymphocytic depletion lymph nodes axilla and arm	Any cancer
110563	B616500	Hodgkin's lymphocytic depletion lymph nodes inguinal and leg	Any cancer
101715	B616700	Hodgkin's disease, lymphocytic depletion of spleen	Any cancer

Medcode	Readcode	Description	Cancer type
107032	B616800	Hodgkin's lymphocytic depletion lymph nodes multiple sites	Any cancer
101530	B616z00	Hodgkin's disease, lymphocytic depletion NOS	Any cancer
104895	B617.00	Nodular lymphocyte predominant Hodgkin lymphoma	Any cancer
105841	B618.00	Nodular sclerosis classical Hodgkin lymphoma	Any cancer
108775	B619.00	Mixed cellularity classical Hodgkin lymphoma	Any cancer
106597	B61B.00	Lymphocyte-rich classical Hodgkin lymphoma	Any cancer
104484	B61C.00	Other classical Hodgkin lymphoma	Any cancer
53397	B61z.00	Hodgkin's disease NOS	Any cancer
106349	B61z.11	Hodgkin lymphoma NOS	Any cancer
61662	B61z000	Hodgkin's disease NOS, unspecified site	Any cancer
59778	B61z100	Hodgkin's disease NOS of lymph nodes of head, face and neck	Any cancer
59755	B61z200	Hodgkin's disease NOS of intrathoracic lymph nodes	Any cancer
107804	B61z300	Hodgkin's disease NOS of intra-abdominal lymph nodes	Any cancer
91900	B61z400	Hodgkin's disease NOS of lymph nodes of axilla and arm	Any cancer
99012	B61z500	Hodgkin's disease NOS of lymph nodes inguinal region and leg	Any cancer
94279	B61z700	Hodgkin's disease NOS of spleen	Any cancer
97746	B61z800	Hodgkin's disease NOS of lymph nodes of multiple sites	Any cancer
42461	B61zz00	Hodgkin's disease NOS	Any cancer
43415	ByuD000	[X]Other Hodgkin's disease	Any cancer
41369	B60..00	Lymphosarcoma and reticulosarcoma	Any cancer
1481	B600.00	Reticulosarcoma	Any cancer
60242	B600000	Reticulosarcoma of unspecified site	Any cancer
71031	B600100	Reticulosarcoma of lymph nodes of head, face and neck	Any cancer
70374	B600300	Reticulosarcoma of intra-abdominal lymph nodes	Any cancer
95058	B600700	Reticulosarcoma of spleen	Any cancer
99240	B600z00	Reticulosarcoma NOS	Any cancer
27416	B601.00	Lymphosarcoma	Any cancer
71625	B601000	Lymphosarcoma of unspecified site	Any cancer
71238	B601100	Lymphosarcoma of lymph nodes of head, face and neck	Any cancer
62380	B601200	Lymphosarcoma of intrathoracic lymph nodes	Any cancer
64670	B601300	Lymphosarcoma of intra-abdominal lymph nodes	Any cancer
100352	B601500	Lymphosarcoma of lymph nodes of inguinal region and leg	Any cancer
103245	B601700	Lymphosarcoma of spleen	Any cancer
104790	B601800	Lymphosarcoma of lymph nodes of multiple sites	Any cancer
63723	B601z00	Lymphosarcoma NOS	Any cancer
21402	B602.00	Burkitt's lymphoma	Any cancer
59115	B602100	Burkitt's lymphoma of lymph nodes of head, face and neck	Any cancer
100006	B602200	Burkitt's lymphoma of intrathoracic lymph nodes	Any cancer
97577	B602300	Burkitt's lymphoma of intra-abdominal lymph nodes	Any cancer
92380	B602500	Burkitt's lymphoma of lymph nodes of inguinal region and leg	Any cancer
71304	B602z00	Burkitt's lymphoma NOS	Any cancer
99887	B60y.00	Other specified reticulosarcoma or lymphosarcoma	Any cancer
99951	B60z.00	Reticulosarcoma or lymphosarcoma NOS	Any cancer
33333	B62..00	Other malignant neoplasm of lymphoid and histiocytic tissue	Any cancer
5179	B620.00	Nodular lymphoma (Brill-Symmers disease)	Any cancer
66327	B620000	Nodular lymphoma of unspecified site	Any cancer

Medcode	Readcode	Description	Cancer type
45264	B620100	Nodular lymphoma of lymph nodes of head, face and neck	Any cancer
105203	B620200	Nodular lymphoma of intrathoracic lymph nodes	Any cancer
92068	B620300	Nodular lymphoma of intra-abdominal lymph nodes	Any cancer
94995	B620500	Nodular lymphoma of lymph nodes of inguinal region and leg	Any cancer
58082	B620800	Nodular lymphoma of lymph nodes of multiple sites	Any cancer
65701	B620z00	Nodular lymphoma NOS	Any cancer
12006	B621.00	Mycosis fungoides	Any cancer
95949	B621000	Mycosis fungoides of unspecified site	Any cancer
91674	B621300	Mycosis fungoides of intra-abdominal lymph nodes	Any cancer
96379	B621400	Mycosis fungoides of lymph nodes of axilla and upper limb	Any cancer
72714	B621500	Mycosis fungoides of lymph nodes of inguinal region and leg	Any cancer
95012	B621800	Mycosis fungoides of lymph nodes of multiple sites	Any cancer
38005	B621z00	Mycosis fungoides NOS	Any cancer
35014	B622.00	Sezary's disease	Any cancer
100532	B622z00	Sezary's disease NOS	Any cancer
44267	B623.00	Malignant histiocytosis	Any cancer
69497	B623000	Malignant histiocytosis of unspecified site	Any cancer
94415	B623100	Malignant histiocytosis of lymph nodes head, face and neck	Any cancer
65642	B623300	Malignant histiocytosis of intra-abdominal lymph nodes	Any cancer
58871	B623z00	Malignant histiocytosis NOS	Any cancer
27330	B624.00	Leukaemic reticuloendotheliosis	Any cancer
5137	B624.11	Leukaemic reticuloendotheliosis	Any cancer
65122	B624000	Leukaemic reticuloendotheliosis of unspecified sites	Any cancer
65123	B624300	Leukaemic reticuloend of intra-abdominal lymph nodes	Any cancer
73777	B624z00	Leukaemic reticuloendotheliosis NOS	Any cancer
34926	B625.00	Letterer-Siwe disease	Any cancer
4870	B625.11	Histiocytosis X (acute, progressive)	Any cancer
102715	B625000	Letterer-Siwe disease of unspecified sites	Any cancer
102158	B625200	Letterer-Siwe disease of intrathoracic lymph nodes	Any cancer
54083	B625800	Letterer-Siwe disease of lymph nodes of multiple sites	Any cancer
47204	B625z00	Letterer-Siwe disease NOS	Any cancer
15036	B626.00	Malignant mast cell tumours	Any cancer
103900	B626000	Mast cell malignancy of unspecified site	Any cancer
100615	B626500	Mast cell malignancy of lymph nodes inguinal region and leg	Any cancer
31324	B626800	Mast cell malignancy of lymph nodes of multiple sites	Any cancer
89657	B626z00	Malignant mast cell tumour NOS	Any cancer
3604	B627.00	Non-Hodgkin's lymphoma	Any cancer
104391	B627.11	Non-Hodgkin lymphoma	Any cancer
28639	B627000	Follicular non-Hodgkin's small cleaved cell lymphoma	Any cancer
70842	B627100	Follicular non-Hodg mixed sml cleavd & lge cell lymphoma	Any cancer
49262	B627200	Follicular non-Hodgkin's large cell lymphoma	Any cancer
50668	B627300	Diffuse non-Hodgkin's small cell (diffuse) lymphoma	Any cancer
108182	B627400	Diffuse non-Hodgkin's small cleaved cell (diffuse) lymphoma	Any cancer
50695	B627500	Diffuse non-Hodgkin mixed sml & lge cell (diffuse) lymphoma	Any cancer
53551	B627600	Diffuse non-Hodgkin's immunoblastic (diffuse) lymphoma	Any cancer
17460	B627700	Diffuse non-Hodgkin's lymphoblastic (diffuse) lymphoma	Any cancer

Medcode	Readcode	Description	Cancer type
65180	B627800	Diffuse non-Hodgkin's lymphoma undifferentiated (diffuse)	Any cancer
95715	B627900	Mucosa-associated lymphoma	Any cancer
95545	B627911	Maltoma	Any cancer
101114	B627A00	Diffuse non-Hodgkin's large cell lymphoma	Any cancer
31576	B627B00	Other types of follicular non-Hodgkin's lymphoma	Any cancer
21549	B627C00	Follicular non-Hodgkin's lymphoma	Any cancer
17182	B627C11	Follicular lymphoma NOS	Any cancer
70509	B627D00	Diffuse non-Hodgkin's centroblastic lymphoma	Any cancer
102594	B627E00	Diffuse large B-cell lymphoma	Any cancer
105966	B627F00	Extranod marg zone B-cell lymphom mucosa-assoc lymphoid tiss	Any cancer
105038	B627G00	Mediastinal (thymic) large B-cell lymphoma	Any cancer
31794	B627W00	Unspecified B-cell non-Hodgkin's lymphoma	Any cancer
39798	B627X00	Diffuse non-Hodgkin's lymphoma, unspecified	Any cancer
104152	B628.00	Follicular lymphoma	Any cancer
105889	B628000	Follicular lymphoma grade 1	Any cancer
105095	B628100	Follicular lymphoma grade 2	Any cancer
107166	B628200	Follicular lymphoma grade 3	Any cancer
105020	B628300	Follicular lymphoma grade 3a	Any cancer
107973	B628400	Follicular lymphoma grade 3b	Any cancer
106969	B628500	Diffuse follicle centre lymphoma	Any cancer
108719	B628600	Cutaneous follicle centre lymphoma	Any cancer
106063	B628700	Other types of follicular lymphoma	Any cancer
105792	B629.00	Multifocal multisystemic dissem Langerhans-cell histiocytosi	Any cancer
105335	B62A.00	Sarcoma of dendritic cells	Any cancer
110191	B62B.00	Multifocal and unisystemic Langerhans-cell histiocytosis	Any cancer
105762	B62C.00	Unifocal Langerhans-cell histiocytosis	Any cancer
105083	B62D.00	Histiocytic sarcoma	Any cancer
105085	B62E.00	T/NK-cell lymphoma	Any cancer
105559	B62E100	Anaplastic large cell lymphoma, ALK-positive	Any cancer
105955	B62E200	Anaplastic large cell lymphoma, ALK-negative	Any cancer
104862	B62E300	Cutaneous T-cell lymphoma	Any cancer
109780	B62E400	Extranodal NK/T-cell lymphoma, nasal type	Any cancer
107949	B62E500	Hepatosplenic T-cell lymphoma	Any cancer
105709	B62E600	Enteropathy-associated T-cell lymphoma	Any cancer
105925	B62E700	Subcutaneous panniculitic T-cell lymphoma	Any cancer
105375	B62E800	Blastic NK-cell lymphoma	Any cancer
105636	B62E900	Angioimmunoblastic T-cell lymphoma	Any cancer
105286	B62EA00	Primary cutaneous CD30-positive T-cell proliferations	Any cancer
104934	B62Ew00	Other mature T/NK-cell lymphoma	Any cancer
106884	B62F.00	Nonfollicular lymphoma	Any cancer
106867	B62F.11	Non-follicular lymphoma	Any cancer
104386	B62F000	Small cell B-cell lymphoma	Any cancer
104620	B62F100	Mantle cell lymphoma	Any cancer
104412	B62F200	Lymphoblastic (diffuse) lymphoma	Any cancer
17887	B62x.00	Malignant lymphoma otherwise specified	Any cancer
90201	B62x000	T-zone lymphoma	Any cancer

Medcode	Readcode	Description	Cancer type
57737	B62x100	Lymphoepithelioid lymphoma	Any cancer
12464	B62x200	Peripheral T-cell lymphoma	Any cancer
62437	B62x400	Malignant reticulosis	Any cancer
58962	B62x500	Malignant immunoproliferative small intestinal disease	Any cancer
95630	B62x600	True histiocytic lymphoma	Any cancer
44318	B62xX00	Oth and unspecif peripheral & cutaneous T-cell lymphomas	Any cancer
12335	B62y.00	Malignant lymphoma NOS	Any cancer
57427	B62y000	Malignant lymphoma NOS of unspecified site	Any cancer
50696	B62y100	Malignant lymphoma NOS of lymph nodes of head, face and neck	Any cancer
72725	B62y200	Malignant lymphoma NOS of intrathoracic lymph nodes	Any cancer
42579	B62y300	Malignant lymphoma NOS of intra-abdominal lymph nodes	Any cancer
34089	B62y400	Malignant lymphoma NOS of lymph nodes of axilla and arm	Any cancer
63105	B62y500	Malignant lymphoma NOS of lymph node inguinal region and leg	Any cancer
71262	B62y600	Malignant lymphoma NOS of intrapelvic lymph nodes	Any cancer
60092	B62y700	Malignant lymphoma NOS of spleen	Any cancer
15504	B62y800	Malignant lymphoma NOS of lymph nodes of multiple sites	Any cancer
15027	B62yz00	Malignant lymphoma NOS	Any cancer
65434	B62z.00	Malignant neoplasms of lymphoid and histiocytic tissue NOS	Any cancer
108037	B62z000	Unspec malig neop lymphoid/histiocytic of unspecified site	Any cancer
64427	B62z100	Unspec malig neop lymphoid/histiocytic lymph node head/neck	Any cancer
93384	B62z200	Unspec malig neop lymphoid/histiocytic of intrathoracic node	Any cancer
103353	B62z300	Unspec malig neop lymphoid/histiocytic intra-abdominal nodes	Any cancer
107638	B62z400	Unspec malig neop lymphoid/histiocytic lymph node axilla/arm	Any cancer
71609	B62z500	Unspec malig neop lymphoid/histiocytic nodes inguinal/leg	Any cancer
109342	B62z600	Unspec malig neop lymphoid/histiocytic of intrapelvic nodes	Any cancer
101465	B62z800	Unspec malig neop lymphoid/histiocytic of multiple sites	Any cancer
95792	B62zz00	Lymphoid and histiocytic malignancy NOS	Any cancer
70716	B62zz11	Immunoproliferative neoplasm	Any cancer
67518	ByuD100	[X]Other types of follicular non-Hodgkin's lymphoma	Any cancer
98596	ByuD200	[X]Other types of diffuse non-Hodgkin's lymphoma	Any cancer
64336	ByuD300	[X]Other specified types of non-Hodgkin's lymphoma	Any cancer
64515	ByuDC00	[X]Diffuse non-Hodgkin's lymphoma, unspecified	Any cancer
109714	ByuDD00	[X]Oth and unspecif peripheral & cutaneous T-cell lymphomas	Any cancer
63375	ByuDE00	[X]Unspecified B-cell non-Hodgkin's lymphoma	Any cancer
8649	ByuDF00	[X]Non-Hodgkin's lymphoma, unspecified type	Any cancer
7940	ByuDF11	[X]Non-Hodgkin's lymphoma NOS	Any cancer
18617	B51..00	Malignant neoplasm of brain	Any cancer
10851	B51..11	Cerebral tumour-malignant	Any cancer
15711	B510.00	Malignant neoplasm cerebrum (excluding lobes and ventricles)	Any cancer
48073	B510000	Malignant neoplasm of basal ganglia	Any cancer
61399	B510100	Malignant neoplasm of cerebral cortex	Any cancer
99913	B510300	Malignant neoplasm of globus pallidus	Any cancer
70942	B510400	Malignant neoplasm of hypothalamus	Any cancer
62126	B510500	Malignant neoplasm of thalamus	Any cancer
54133	B510z00	Malignant neoplasm of cerebrum NOS	Any cancer
42426	B511.00	Malignant neoplasm of frontal lobe	Any cancer

Medcode	Readcode	Description	Cancer type
46792	B512.00	Malignant neoplasm of temporal lobe	Any cancer
67236	B512000	Malignant neoplasm of hippocampus	Any cancer
47556	B512z00	Malignant neoplasm of temporal lobe NOS	Any cancer
19226	B513.00	Malignant neoplasm of parietal lobe	Any cancer
39088	B514.00	Malignant neoplasm of occipital lobe	Any cancer
52511	B515.00	Malignant neoplasm of cerebral ventricles	Any cancer
46789	B515000	Malignant neoplasm of choroid plexus	Any cancer
45154	B516.00	Malignant neoplasm of cerebellum	Any cancer
44089	B517.00	Malignant neoplasm of brain stem	Any cancer
64557	B517000	Malignant neoplasm of cerebral peduncle	Any cancer
49132	B517100	Malignant neoplasm of medulla oblongata	Any cancer
93537	B517200	Malignant neoplasm of midbrain	Any cancer
91240	B517300	Malignant neoplasm of pons	Any cancer
68641	B517z00	Malignant neoplasm of brain stem NOS	Any cancer
71139	B51y.00	Malignant neoplasm of other parts of brain	Any cancer
59170	B51y000	Malignant neoplasm of corpus callosum	Any cancer
65241	B51y200	Malignant neoplasm, overlapping lesion of brain	Any cancer
100733	B51yz00	Malignant neoplasm of other part of brain NOS	Any cancer
41520	B51z.00	Malignant neoplasm of brain NOS	Any cancer
65458	B52..00	Malig neop of other and unspecified parts of nervous system	Any cancer
99621	B520.00	Malignant neoplasm of cranial nerves	Any cancer
64971	B520000	Malignant neoplasm of olfactory bulb	Any cancer
70126	B520100	Malignant neoplasm of optic nerve	Any cancer
65599	B520200	Malignant neoplasm of acoustic nerve	Any cancer
101086	B520z00	Malignant neoplasm of cranial nerves NOS	Any cancer
28919	B521.00	Malignant neoplasm of cerebral meninges	Any cancer
109473	B521200	Malignant neoplasm of cerebral pia mater	Any cancer
70104	B521z00	Malignant neoplasm of cerebral meninges NOS	Any cancer
41515	ByuA100	[X]Malignant neoplasm/central nervous system, unspecified	Any cancer
63925	ByuA200	[X]Malignant neoplasm of meninges, unspecified	Any cancer
47633	ByuA300	[X]Malig neopl, overlap lesion brain & other part of CNS	Any cancer
3968	B34..00	Malignant neoplasm of female breast	Any cancer
348	B34..11	Ca female breast	Any cancer
26853	B340.00	Malignant neoplasm of nipple and areola of female breast	Any cancer
23380	B340000	Malignant neoplasm of nipple of female breast	Any cancer
64686	B340100	Malignant neoplasm of areola of female breast	Any cancer
59831	B340z00	Malignant neoplasm of nipple or areola of female breast NOS	Any cancer
31546	B341.00	Malignant neoplasm of central part of female breast	Any cancer
29826	B342.00	Malignant neoplasm of upper-inner quadrant of female breast	Any cancer
45222	B343.00	Malignant neoplasm of lower-inner quadrant of female breast	Any cancer
23399	B344.00	Malignant neoplasm of upper-outer quadrant of female breast	Any cancer
42070	B345.00	Malignant neoplasm of lower-outer quadrant of female breast	Any cancer
20685	B346.00	Malignant neoplasm of axillary tail of female breast	Any cancer
49148	B347.00	Malignant neoplasm, overlapping lesion of breast	Any cancer
56715	B34y.00	Malignant neoplasm of other site of female breast	Any cancer
95057	B34y000	Malignant neoplasm of ectopic site of female breast	Any cancer

Medcode	Readcode	Description	Cancer type
38475	B34yz00	Malignant neoplasm of other site of female breast NOS	Any cancer
9470	B34z.00	Malignant neoplasm of female breast NOS	Any cancer
19423	B35..00	Malignant neoplasm of male breast	Any cancer
54494	B350.00	Malignant neoplasm of nipple and areola of male breast	Any cancer
68480	B350000	Malignant neoplasm of nipple of male breast	Any cancer
67884	B350100	Malignant neoplasm of areola of male breast	Any cancer
54202	B35z.00	Malignant neoplasm of other site of male breast	Any cancer
95323	B35z000	Malignant neoplasm of ectopic site of male breast	Any cancer
48809	B35zz00	Malignant neoplasm of male breast NOS	Any cancer
105488	B36..00	Local recurrence of malignant tumour of breast	Any cancer
7833	B830.00	Carcinoma in situ of breast	Any cancer
10387	B830000	Lobular carcinoma in situ of breast	Any cancer
18694	B830100	Intraductal carcinoma in situ of breast	Any cancer
12499	Byu6.00	[X]Malignant neoplasm of breast	Any cancer
53803	ByuFG00	[X]Other carcinoma in situ of breast	Any cancer
108667	B1z1000	Angiosarcoma of spleen	Any cancer
72224	B1z1100	Fibrosarcoma of spleen	Any cancer
12539	B3...12	Sarcoma of bone and connective tissue	Any cancer
18314	B30..00	Malignant neoplasm of bone and articular cartilage	Any cancer
5062	B30..11	Chondroma	Any cancer
29735	B30..12	Osteoma	Any cancer
59036	B300.00	Malignant neoplasm of bones of skull and face	Any cancer
53594	B300000	Malignant neoplasm of ethmoid bone	Any cancer
53599	B300100	Malignant neoplasm of frontal bone	Any cancer
59520	B300200	Malignant neoplasm of malar bone	Any cancer
95458	B300300	Malignant neoplasm of nasal bone	Any cancer
55953	B300400	Malignant neoplasm of occipital bone	Any cancer
50298	B300500	Malignant neoplasm of orbital bone	Any cancer
54747	B300600	Malignant neoplasm of parietal bone	Any cancer
55595	B300700	Malignant neoplasm of sphenoid bone	Any cancer
62104	B300800	Malignant neoplasm of temporal bone	Any cancer
50299	B300900	Malignant neoplasm of zygomatic bone	Any cancer
17475	B300A00	Malignant neoplasm of maxilla	Any cancer
96445	B300B00	Malignant neoplasm of turbinate	Any cancer
44452	B300C00	Malignant neoplasm of vomer	Any cancer
69146	B300z00	Malignant neoplasm of bones of skull and face NOS	Any cancer
33833	B301.00	Malignant neoplasm of mandible	Any cancer
16704	B302.00	Malignant neoplasm of vertebral column	Any cancer
46939	B302000	Malignant neoplasm of cervical vertebra	Any cancer
32372	B302100	Malignant neoplasm of thoracic vertebra	Any cancer
54691	B302200	Malignant neoplasm of lumbar vertebra	Any cancer
49701	B302z00	Malignant neoplasm of vertebral column NOS	Any cancer
27528	B303.00	Malignant neoplasm of ribs, sternum and clavicle	Any cancer
37842	B303000	Malignant neoplasm of rib	Any cancer
49491	B303100	Malignant neoplasm of sternum	Any cancer
66639	B303200	Malignant neoplasm of clavicle	Any cancer

Medcode	Readcode	Description	Cancer type
60403	B303300	Malignant neoplasm of costal cartilage	Any cancer
67763	B303400	Malignant neoplasm of costo-vertebral joint	Any cancer
54493	B303500	Malignant neoplasm of xiphoid process	Any cancer
51237	B303z00	Malignant neoplasm of rib, sternum and clavicle NOS	Any cancer
71810	B304.00	Malignant neoplasm of scapula and long bones of upper arm	Any cancer
49054	B304000	Malignant neoplasm of scapula	Any cancer
105797	B304100	Malignant neoplasm of acromion	Any cancer
61741	B304200	Malignant neoplasm of humerus	Any cancer
92371	B304300	Malignant neoplasm of radius	Any cancer
64848	B304400	Malignant neoplasm of ulna	Any cancer
65880	B304z00	Malig neop of scapula and long bones of upper arm NOS	Any cancer
73530	B305.00	Malignant neoplasm of hand bones	Any cancer
106069	B305.11	Malignant neoplasm of carpal bones	Any cancer
72464	B305.12	Malignant neoplasm of metacarpal bones	Any cancer
57988	B305000	Malignant neoplasm of carpal bone-scaphoid	Any cancer
69104	B305100	Malignant neoplasm of carpal bone-lunate	Any cancer
108638	B305A00	Malignant neoplasm of third metacarpal bone	Any cancer
94427	B305C00	Malignant neoplasm of fifth metacarpal bone	Any cancer
86812	B305D00	Malignant neoplasm of phalanges of hand	Any cancer
73556	B305z00	Malignant neoplasm of hand bones NOS	Any cancer
54631	B306.00	Malignant neoplasm of pelvic bones, sacrum and coccyx	Any cancer
44609	B306000	Malignant neoplasm of ilium	Any cancer
59223	B306100	Malignant neoplasm of ischium	Any cancer
51921	B306200	Malignant neoplasm of pubis	Any cancer
40966	B306300	Malignant neoplasm of sacral vertebra	Any cancer
66908	B306400	Malignant neoplasm of coccygeal vertebra	Any cancer
50152	B306500	Malignant sacral teratoma	Any cancer
38938	B306z00	Malignant neoplasm of pelvis, sacrum or coccyx NOS	Any cancer
68055	B307.00	Malignant neoplasm of long bones of leg	Any cancer
56513	B307000	Malignant neoplasm of femur	Any cancer
50402	B307100	Malignant neoplasm of fibula	Any cancer
40814	B307200	Malignant neoplasm of tibia	Any cancer
62630	B307z00	Malignant neoplasm of long bones of leg NOS	Any cancer
105475	B308.00	Malignant neoplasm of short bones of leg	Any cancer
95182	B308100	Malignant neoplasm of talus	Any cancer
72212	B308200	Malignant neoplasm of calcaneum	Any cancer
34878	B308300	Malignant neoplasm of medial cuneiform	Any cancer
69927	B308800	Malignant neoplasm of first metatarsal bone	Any cancer
92382	B308B00	Malignant neoplasm of fourth metatarsal bone	Any cancer
58949	B308D00	Malignant neoplasm of phalanges of foot	Any cancer
103354	B308z00	Malignant neoplasm of short bones of leg NOS	Any cancer
67451	B30W.00	Malignant neoplasm/overlap lesion/bone+articulr cartilage	Any cancer
43614	B30X.00	Malignant neoplasm/bones+articular cartilage/limb,unspfd	Any cancer
16075	B30z.00	Malignant neoplasm of bone and articular cartilage NOS	Any cancer
19437	B30z000	Osteosarcoma	Any cancer
34451	B31..00	Malignant neoplasm of connective and other soft tissue	Any cancer

Medcode	Readcode	Description	Cancer type
43475	B310.00	Malig neop of connective and soft tissue head, face and neck	Any cancer
59382	B310000	Malignant neoplasm of soft tissue of head	Any cancer
40014	B310100	Malignant neoplasm of soft tissue of face	Any cancer
48517	B310200	Malignant neoplasm of soft tissue of neck	Any cancer
60035	B310300	Malignant neoplasm of cartilage of ear	Any cancer
49463	B310400	Malignant neoplasm of tarsus of eyelid	Any cancer
108389	B310500	Malignant neoplasm soft tissues of cervical spine	Any cancer
73718	B310z00	Malig neop connective and soft tissue head, face, neck NOS	Any cancer
53989	B311.00	Malig neop connective and soft tissue upper limb/shoulder	Any cancer
50222	B311000	Malignant neoplasm of connective and soft tissue of shoulder	Any cancer
64345	B311100	Malignant neoplasm of connective and soft tissue, upper arm	Any cancer
57482	B311200	Malignant neoplasm of connective and soft tissue of fore-arm	Any cancer
19321	B311300	Malignant neoplasm of connective and soft tissue of hand	Any cancer
91586	B311400	Malignant neoplasm of connective and soft tissue of finger	Any cancer
63988	B311500	Malignant neoplasm of connective and soft tissue of thumb	Any cancer
104913	B311z00	Malig neop connective soft tissue upper limb/shoulder NOS	Any cancer
66088	B312.00	Malig neop of connective and soft tissue of hip and leg	Any cancer
102949	B312000	Malignant neoplasm of connective and soft tissue of hip	Any cancer
44805	B312100	Malig neop of connective and soft tissue thigh and upper leg	Any cancer
54965	B312200	Malig neop connective and soft tissue of popliteal space	Any cancer
30542	B312300	Malig neop of connective and soft tissue of lower leg	Any cancer
54222	B312400	Malignant neoplasm of connective and soft tissue of foot	Any cancer
99572	B312500	Malignant neoplasm of connective and soft tissue of toe	Any cancer
90546	B312z00	Malig neop connective and soft tissue hip and leg NOS	Any cancer
22290	B313.00	Malignant neoplasm of connective and soft tissue of thorax	Any cancer
29160	B313000	Malignant neoplasm of connective and soft tissue of axilla	Any cancer
54186	B313100	Malignant neoplasm of diaphragm	Any cancer
72522	B313200	Malignant neoplasm of great vessels	Any cancer
104139	B313300	Malig neoplasm of connective and soft tissues of thor spine	Any cancer
98408	B313z00	Malig neop of connective and soft tissue of thorax NOS	Any cancer
45071	B314.00	Malignant neoplasm of connective and soft tissue of abdomen	Any cancer
66488	B314000	Malig neop of connective and soft tissue of abdominal wall	Any cancer
94272	B314100	Malig neoplasm of connective and soft tissues of lumb spine	Any cancer
60247	B314z00	Malig neop of connective and soft tissue of abdomen NOS	Any cancer
51965	B315.00	Malignant neoplasm of connective and soft tissue of pelvis	Any cancer
70463	B315000	Malignant neoplasm of connective and soft tissue of buttock	Any cancer
67324	B315100	Malig neop of connective and soft tissue of inguinal region	Any cancer
59152	B315200	Malignant neoplasm of connective and soft tissue of perineum	Any cancer
110192	B315300	Malig neopl of connective and soft tissue-sacrum or coccyx	Any cancer
58836	B315z00	Malig neop of connective and soft tissue of pelvis NOS	Any cancer
57471	B316.00	Malig neop of connective and soft tissue trunk unspecified	Any cancer
65233	B31y.00	Malig neop connective and soft tissue other specified site	Any cancer
15182	B31z.00	Malignant neoplasm of connective and soft tissue, site NOS	Any cancer
104128	B31z000	Kaposi's sarcoma of soft tissue	Any cancer
27931	B33z000	Kaposi's sarcoma of skin	Any cancer
40749	Byu3.00	[X]Malignant neoplasm of bone and articular cartilage	Any cancer

Medcode	Readcode	Description	Cancer type
73296	Byu3100	[X]Malignant neoplasm/bones+articular cartilage/limb,unspfd	Any cancer
63300	Byu3200	[X]Malignant neoplasm/overlap lesion/bone+articulr cartilage	Any cancer
43151	Byu3300	[X]Malignant neoplasm/bone+articular cartilage, unspecified	Any cancer
20160	B50..00	Malignant neoplasm of eye	Any cancer
98813	B500.00	Malig neop eyeball excl conjunctiva, cornea, retina, choroid	Any cancer
59041	B500000	Malignant neoplasm of ciliary body	Any cancer
59381	B500100	Malignant neoplasm of iris	Any cancer
106569	B500200	Malignant neoplasm of crystalline lens	Any cancer
56718	B500z00	Malignant neoplasm of eyeball NOS	Any cancer
45667	B501.00	Malignant neoplasm of orbit	Any cancer
86996	B501000	Malignant neoplasm of connective tissue of orbit	Any cancer
63104	B501z00	Malignant neoplasm of orbit NOS	Any cancer
64817	B502.00	Malignant neoplasm of lacrimal gland	Any cancer
63657	B503.00	Malignant neoplasm of conjunctiva	Any cancer
73992	B504.00	Malignant neoplasm of cornea	Any cancer
28069	B505.00	Malignant neoplasm of retina	Any cancer
15991	B506.00	Malignant neoplasm of choroid	Any cancer
71584	B507.00	Malignant neoplasm of lacrimal duct	Any cancer
101805	B507000	Malignant neoplasm of lacrimal sac	Any cancer
65357	B507100	Malignant neoplasm of nasolacrimal duct	Any cancer
45922	B508.00	Malignant neoplasm, overlapping lesion of eye and adnexa	Any cancer
40437	B50y.00	Malignant neoplasm of other specified site of eye	Any cancer
54956	B50z.00	Malignant neoplasm of eye NOS	Any cancer
15709	B1...00	Malignant neoplasm of digestive organs and peritoneum	Any cancer
3357	B1...11	Carcinoma of digestive organs and peritoneum	Any cancer
1062	B10..00	Malignant neoplasm of oesophagus	Any cancer
61695	B100.00	Malignant neoplasm of cervical oesophagus	Any cancer
41362	B101.00	Malignant neoplasm of thoracic oesophagus	Any cancer
63470	B102.00	Malignant neoplasm of abdominal oesophagus	Any cancer
50789	B103.00	Malignant neoplasm of upper third of oesophagus	Any cancer
54171	B104.00	Malignant neoplasm of middle third of oesophagus	Any cancer
42416	B105.00	Malignant neoplasm of lower third of oesophagus	Any cancer
67497	B106.00	Malignant neoplasm, overlapping lesion of oesophagus	Any cancer
98142	B107.00	Siewert type I adenocarcinoma	Any cancer
53591	B10y.00	Malignant neoplasm of other specified part of oesophagus	Any cancer
30700	B10z.00	Malignant neoplasm of oesophagus NOS	Any cancer
4865	B10z.11	Oesophageal cancer	Any cancer
8386	B11..00	Malignant neoplasm of stomach	Any cancer
10368	B11..11	Gastric neoplasm	Any cancer
32022	B110.00	Malignant neoplasm of cardia of stomach	Any cancer
100584	B110000	Malignant neoplasm of cardiac orifice of stomach	Any cancer
22894	B110100	Malignant neoplasm of cardio-oesophageal junction of stomach	Any cancer
94278	B110111	Malignant neoplasm of gastro-oesophageal junction	Any cancer
37859	B110z00	Malignant neoplasm of cardia of stomach NOS	Any cancer
21620	B111.00	Malignant neoplasm of pylorus of stomach	Any cancer
48237	B111000	Malignant neoplasm of prepylorus of stomach	Any cancer

Medcode	Readcode	Description	Cancer type
41215	B111100	Malignant neoplasm of pyloric canal of stomach	Any cancer
59092	B111z00	Malignant neoplasm of pylorus of stomach NOS	Any cancer
19318	B112.00	Malignant neoplasm of pyloric antrum of stomach	Any cancer
32362	B113.00	Malignant neoplasm of fundus of stomach	Any cancer
43572	B114.00	Malignant neoplasm of body of stomach	Any cancer
42193	B115.00	Malignant neoplasm of lesser curve of stomach unspecified	Any cancer
55434	B116.00	Malignant neoplasm of greater curve of stomach unspecified	Any cancer
51690	B117.00	Malignant neoplasm, overlapping lesion of stomach	Any cancer
97499	B118.00	Siewert type II adenocarcinoma	Any cancer
96094	B119.00	Siewert type III adenocarcinoma	Any cancer
55019	B11y.00	Malignant neoplasm of other specified site of stomach	Any cancer
65312	B11y000	Malignant neoplasm of anterior wall of stomach NEC	Any cancer
96802	B11y100	Malignant neoplasm of posterior wall of stomach NEC	Any cancer
65372	B11yz00	Malignant neoplasm of other specified site of stomach NOS	Any cancer
14800	B11z.00	Malignant neoplasm of stomach NOS	Any cancer
6806	B12..00	Malignant neoplasm of small intestine and duodenum	Any cancer
18613	B120.00	Malignant neoplasm of duodenum	Any cancer
43479	B121.00	Malignant neoplasm of jejunum	Any cancer
33871	B122.00	Malignant neoplasm of ileum	Any cancer
63995	B123.00	Malignant neoplasm of Meckel's diverticulum	Any cancer
66166	B124.00	Malignant neoplasm, overlapping lesion of small intestine	Any cancer
99896	B12y.00	Malignant neoplasm of other specified site small intestine	Any cancer
43390	B12z.00	Malignant neoplasm of small intestine NOS	Any cancer
1220	B13..00	Malignant neoplasm of colon	Any cancer
9088	B130.00	Malignant neoplasm of hepatic flexure of colon	Any cancer
6935	B131.00	Malignant neoplasm of transverse colon	Any cancer
10864	B132.00	Malignant neoplasm of descending colon	Any cancer
2815	B133.00	Malignant neoplasm of sigmoid colon	Any cancer
3811	B134.00	Malignant neoplasm of caecum	Any cancer
22163	B134.11	Carcinoma of caecum	Any cancer
18632	B135.00	Malignant neoplasm of appendix	Any cancer
10946	B136.00	Malignant neoplasm of ascending colon	Any cancer
18619	B137.00	Malignant neoplasm of splenic flexure of colon	Any cancer
93478	B138.00	Malignant neoplasm, overlapping lesion of colon	Any cancer
101700	B139.00	Hereditary nonpolyposis colon cancer	Any cancer
48231	B13y.00	Malignant neoplasm of other specified sites of colon	Any cancer
28163	B13z.00	Malignant neoplasm of colon NOS	Any cancer
9118	B13z.11	Colonic cancer	Any cancer
35357	B14..00	Malignant neoplasm of rectum, rectosigmoid junction and anus	Any cancer
27855	B140.00	Malignant neoplasm of rectosigmoid junction	Any cancer
1800	B141.00	Malignant neoplasm of rectum	Any cancer
7219	B141.11	Carcinoma of rectum	Any cancer
5901	B141.12	Rectal carcinoma	Any cancer
24370	B142.00	Malignant neoplasm of anal canal	Any cancer
9491	B142.11	Anal carcinoma	Any cancer
46159	B142000	Malignant neoplasm of cloacogenic zone	Any cancer

Medcode	Readcode	Description	Cancer type
27897	B143.00	Malignant neoplasm of anus unspecified	Any cancer
55659	B14y.00	Malig neop other site rectum, rectosigmoid junction and anus	Any cancer
50974	B14z.00	Malignant neoplasm rectum,rectosigmoid junction and anus NOS	Any cancer
17559	B1z0.00	Malignant neoplasm of intestinal tract, part unspecified	Any cancer
11628	B1z0.11	Cancer of bowel	Any cancer
94776	B1z2.00	Malignant neoplasm, overlapping lesion of digestive system	Any cancer
56918	B1zy.00	Malignant neoplasm other spec digestive tract and peritoneum	Any cancer
51255	B1zz.00	Malignant neoplasm of digestive tract and peritoneum NOS	Any cancer
8244	B801.00	Carcinoma in situ of oesophagus	Any cancer
99155	B801000	Carcinoma in situ of upper 1/3 oesophagus	Any cancer
64274	B801100	Carcinoma in situ of middle 1/3 oesophagus	Any cancer
56077	B801200	Carcinoma in situ of lower 1/3 oesophagus	Any cancer
44228	B801z00	Carcinoma in situ of oesophagus NOS	Any cancer
17093	B802.00	Carcinoma in situ of stomach	Any cancer
17258	B802000	Carcinoma in situ of cardia of stomach	Any cancer
72947	B802100	Carcinoma in situ of fundus of stomach	Any cancer
63087	B802200	Carcinoma in situ of body of stomach	Any cancer
51748	B802300	Carcinoma in situ of pyloric antrum	Any cancer
58883	B802400	Carcinoma in situ of pyloric canal	Any cancer
37774	B802z00	Carcinoma in situ of stomach NOS	Any cancer
6903	B803.00	Carcinoma in situ of colon	Any cancer
39080	B803000	Carcinoma in situ of hepatic flexure of colon	Any cancer
37125	B803100	Carcinoma in situ of transverse colon	Any cancer
47667	B803200	Carcinoma in situ of descending colon	Any cancer
17144	B803300	Carcinoma in situ of sigmoid colon	Any cancer
16916	B803400	Carcinoma in situ of caecum	Any cancer
47656	B803500	Carcinoma in situ of appendix	Any cancer
31893	B803600	Carcinoma in situ of ascending colon	Any cancer
22699	B803700	Carcinoma in situ of splenic flexure of colon	Any cancer
105228	B803800	High grade dysplasia of colon	Any cancer
33561	B803z00	Carcinoma in situ of colon NOS	Any cancer
60477	B804.00	Carcinoma in situ of rectum and rectosigmoid junction	Any cancer
27811	B804000	Carcinoma in situ of rectosigmoid junction	Any cancer
29975	B804100	Carcinoma in situ of rectum	Any cancer
38883	B804z00	Carcinoma in situ of rectum or rectosigmoid junction NOS	Any cancer
51054	B805.00	Carcinoma in situ of anal canal	Any cancer
34094	B805000	Anal intraepithelial neoplasia grade III	Any cancer
12273	B806.00	Carcinoma in situ of anus NOS	Any cancer
22392	B807.00	Carcinoma in situ of other and unspecified small intestine	Any cancer
45070	B807000	Carcinoma in situ of duodenum	Any cancer
63804	B807100	Carcinoma in situ of jejunum	Any cancer
45217	B807200	Carcinoma in situ of ileum	Any cancer
100183	B807300	Carcinoma in situ of Meckel's diverticulum	Any cancer
70728	B807z00	Carcinoma in situ other and unspecified small intestine NOS	Any cancer
35180	Byu1.00	[X]Malignant neoplasm of digestive organs	Any cancer
45766	Byu1200	[X]Malignant neoplasm of intestinal tract, part unspecified	Any cancer

Medcode	Readcode	Description	Cancer type
49292	Byu1300	[X]Malignant neoplsm/ill-defin sites within digestive system	Any cancer
102708	ByuF100	[X]Carcinoma in situ of other specified digestive organs	Any cancer
2744	B40..00	Malignant neoplasm of uterus, part unspecified	Any cancer
2747	B41..00	Malignant neoplasm of cervix uteri	Any cancer
3230	B41..11	Cervical carcinoma (uterus)	Any cancer
48820	B410.00	Malignant neoplasm of endocervix	Any cancer
57235	B410000	Malignant neoplasm of endocervical canal	Any cancer
53103	B410100	Malignant neoplasm of endocervical gland	Any cancer
50285	B410z00	Malignant neoplasm of endocervix NOS	Any cancer
50297	B411.00	Malignant neoplasm of exocervix	Any cancer
58094	B412.00	Malignant neoplasm, overlapping lesion of cervix uteri	Any cancer
32955	B41y.00	Malignant neoplasm of other site of cervix	Any cancer
95505	B41y000	Malignant neoplasm of cervical stump	Any cancer
57719	B41y100	Malignant neoplasm of squamocolumnar junction of cervix	Any cancer
43435	B41yz00	Malignant neoplasm of other site of cervix NOS	Any cancer
28311	B41z.00	Malignant neoplasm of cervix uteri NOS	Any cancer
93762	B42..00	Malignant neoplasm of placenta	Any cancer
28003	B420.00	Choriocarcinoma	Any cancer
7046	B43..00	Malignant neoplasm of body of uterus	Any cancer
3213	B430.00	Malignant neoplasm of corpus uteri, excluding isthmus	Any cancer
72723	B430000	Malignant neoplasm of cornu of corpus uteri	Any cancer
68155	B430100	Malignant neoplasm of fundus of corpus uteri	Any cancer
2890	B430200	Malignant neoplasm of endometrium of corpus uteri	Any cancer
49400	B430211	Malignant neoplasm of endometrium	Any cancer
45793	B430300	Malignant neoplasm of myometrium of corpus uteri	Any cancer
45490	B430z00	Malignant neoplasm of corpus uteri NOS	Any cancer
43940	B431.00	Malignant neoplasm of isthmus of uterine body	Any cancer
59097	B431000	Malignant neoplasm of lower uterine segment	Any cancer
70729	B431z00	Malignant neoplasm of isthmus of uterine body NOS	Any cancer
16967	B432.00	Malignant neoplasm of overlapping lesion of corpus uteri	Any cancer
31608	B43y.00	Malignant neoplasm of other site of uterine body	Any cancer
33617	B43z.00	Malignant neoplasm of body of uterus NOS	Any cancer
19141	B44..00	Malignant neoplasm of ovary and other uterine adnexa	Any cancer
7805	B440.00	Malignant neoplasm of ovary	Any cancer
1986	B440.11	Cancer of ovary	Any cancer
49828	B441.00	Malignant neoplasm of fallopian tube	Any cancer
101778	B442.00	Malignant neoplasm of broad ligament	Any cancer
46153	B443.00	Malignant neoplasm of parametrium	Any cancer
97996	B44y.00	Malignant neoplasm of other site of uterine adnexa	Any cancer
65106	B44z.00	Malignant neoplasm of uterine adnexa NOS	Any cancer
4555	B45..00	Malig neop of other and unspecified female genital organs	Any cancer
37328	B450.00	Malignant neoplasm of vagina	Any cancer
10698	B450100	Malignant neoplasm of vaginal vault	Any cancer
60772	B450z00	Malignant neoplasm of vagina NOS	Any cancer
43761	B451.00	Malignant neoplasm of labia majora	Any cancer
47899	B451000	Malignant neoplasm of greater vestibular (Bartholin's) gland	Any cancer

Medcode	Readcode	Description	Cancer type
59362	B451z00	Malignant neoplasm of labia majora NOS	Any cancer
58061	B452.00	Malignant neoplasm of labia minora	Any cancer
53910	B453.00	Malignant neoplasm of clitoris	Any cancer
4554	B454.00	Malignant neoplasm of vulva unspecified	Any cancer
11991	B454.11	Primary vulval cancer	Any cancer
26454	B45X.00	Malignant neoplasm/overlapping lesion/feml genital organs	Any cancer
95421	B45y.00	Malignant neoplasm of other specified female genital organ	Any cancer
27617	B45y000	Malignant neoplasm of overlapping lesion of vulva	Any cancer
20166	B45z.00	Malignant neoplasm of female genital organ NOS	Any cancer
3279	B831.00	Carcinoma in situ of cervix uteri	Any cancer
4087	B831.11	CIN III-carcinoma in situ of cervix	Any cancer
5295	B831.12	Cervical intraepithelial neoplasia	Any cancer
21886	B831.13	Cervical intraepithelial neoplasia grade III	Any cancer
24228	B831000	Carcinoma in situ of endocervix	Any cancer
50126	B831100	Carcinoma in situ of exocervix	Any cancer
29898	B832.00	Carcinoma in situ of other and unspecified parts of uterus	Any cancer
61803	B832.11	Carcinoma in situ of body of uterus	Any cancer
7904	B832000	Carcinoma in situ of endometrium	Any cancer
44915	B833.00	Carcinoma in situ other and unspecified female genital organ	Any cancer
17137	B833000	Carcinoma in situ of ovary	Any cancer
59499	B833100	Carcinoma in situ of fallopian tube	Any cancer
34946	B833200	Carcinoma in situ of vagina	Any cancer
12119	B833300	Carcinoma in situ of vulva	Any cancer
3281	B833311	Vulval intraepithelial neoplasia	Any cancer
40598	Byu7.00	[X]Malignant neoplasm of female genital organs	Any cancer
64497	Byu7000	[X]Malignant neoplasm of uterine adnexa, unspecified	Any cancer
57756	Byu7100	[X]Malignant neoplasm/other specified female genital organs	Any cancer
55588	Byu7300	[X]Malignant neoplasm of female genital organ, unspecified	Any cancer
72695	ByuFA00	[X]Carcinoma in situ of other parts of cervix	Any cancer
8918	B15..00	Malignant neoplasm of liver and intrahepatic bile ducts	Any cancer
25535	B150.00	Primary malignant neoplasm of liver	Any cancer
16126	B150000	Primary carcinoma of liver	Any cancer
31210	B150100	Hepatoblastoma of liver	Any cancer
68410	B150200	Primary angiosarcoma of liver	Any cancer
22187	B150300	Hepatocellular carcinoma	Any cancer
44399	B150z00	Primary malignant neoplasm of liver NOS	Any cancer
16915	B151.00	Malignant neoplasm of intrahepatic bile ducts	Any cancer
65124	B151000	Malignant neoplasm of interlobular bile ducts	Any cancer
89593	B151200	Malignant neoplasm of intrahepatic biliary passages	Any cancer
58088	B151400	Malignant neoplasm of intrahepatic gall duct	Any cancer
61643	B151z00	Malignant neoplasm of intrahepatic bile ducts NOS	Any cancer
26393	B152.00	Malignant neoplasm of liver unspecified	Any cancer
36147	B153.00	Secondary malignant neoplasm of liver	Any cancer
38978	B15z.00	Malignant neoplasm of liver and intrahepatic bile ducts NOS	Any cancer
54103	B16..00	Malignant neoplasm gallbladder and extrahepatic bile ducts	Any cancer
16105	B160.00	Malignant neoplasm of gallbladder	Any cancer

Medcode	Readcode	Description	Cancer type
31393	B160.11	Carcinoma gallbladder	Any cancer
23433	B161.00	Malignant neoplasm of extrahepatic bile ducts	Any cancer
72445	B161000	Malignant neoplasm of cystic duct	Any cancer
52537	B161100	Malignant neoplasm of hepatic duct	Any cancer
7982	B161200	Malignant neoplasm of common bile duct	Any cancer
36495	B161211	Carcinoma common bile duct	Any cancer
105613	B161300	Malignant neoplasm of sphincter of Oddi	Any cancer
74896	B161z00	Malignant neoplasm of extrahepatic bile ducts NOS	Any cancer
10949	B162.00	Malignant neoplasm of ampulla of Vater	Any cancer
35039	B163.00	Malignant neoplasm, overlapping lesion of biliary tract	Any cancer
60312	B16y.00	Malignant neoplasm other gallbladder/extrahepatic bile duct	Any cancer
15907	B16z.00	Malignant neoplasm gallbladder/extrahepatic bile ducts NOS	Any cancer
8166	B17..00	Malignant neoplasm of pancreas	Any cancer
8771	B170.00	Malignant neoplasm of head of pancreas	Any cancer
40810	B171.00	Malignant neoplasm of body of pancreas	Any cancer
39870	B172.00	Malignant neoplasm of tail of pancreas	Any cancer
35535	B173.00	Malignant neoplasm of pancreatic duct	Any cancer
35795	B174.00	Malignant neoplasm of Islets of Langerhans	Any cancer
97875	B175.00	Malignant neoplasm, overlapping lesion of pancreas	Any cancer
109782	B176.00	Somatostatinoma of pancreas	Any cancer
48537	B17y.00	Malignant neoplasm of other specified sites of pancreas	Any cancer
96635	B17y000	Malignant neoplasm of ectopic pancreatic tissue	Any cancer
95783	B17yz00	Malignant neoplasm of specified site of pancreas NOS	Any cancer
34388	B17z.00	Malignant neoplasm of pancreas NOS	Any cancer
66673	B808.00	Carcinoma in situ of liver and biliary system	Any cancer
51934	B808.11	Carcinoma in situ of biliary system	Any cancer
25310	B808000	Carcinoma in situ of liver	Any cancer
99580	B808100	Carcinoma in situ of intrahepatic bile ducts	Any cancer
37501	B808200	Carcinoma in situ of hepatic duct	Any cancer
46594	B808300	Carcinoma in situ of gall bladder	Any cancer
73164	B808400	Carcinoma in situ of cystic duct	Any cancer
64089	B808500	Carcinoma in situ of common bile duct	Any cancer
21792	B808600	Carcinoma in situ of ampulla of Vater	Any cancer
98540	B808z00	Carcinoma in situ of liver or biliary system NOS	Any cancer
44166	B80z.00	Carcinoma in situ of other and unspecified digestive organs	Any cancer
16931	B80z000	Carcinoma in situ of pancreas	Any cancer
43490	Byu1100	[X]Other specified carcinomas of liver	Any cancer
15148	B47..00	Malignant neoplasm of testis	Any cancer
64602	B470.00	Malignant neoplasm of undescended testis	Any cancer
7740	B470200	Seminoma of undescended testis	Any cancer
36325	B470300	Teratoma of undescended testis	Any cancer
96429	B470z00	Malignant neoplasm of undescended testis NOS	Any cancer
19475	B471.00	Malignant neoplasm of descended testis	Any cancer
21786	B471000	Seminoma of descended testis	Any cancer
9476	B471100	Teratoma of descended testis	Any cancer
91509	B471z00	Malignant neoplasm of descended testis NOS	Any cancer

Medcode	Readcode	Description	Cancer type
38510	B47z.00	Malignant neoplasm of testis NOS	Any cancer
2961	B47z.11	Seminoma of testis	Any cancer
15989	B47z.12	Teratoma of testis	Any cancer
3541	B48..00	Malignant neoplasm of penis and other male genital organs	Any cancer
50681	B480.00	Malignant neoplasm of prepuce (foreskin)	Any cancer
17841	B481.00	Malignant neoplasm of glans penis	Any cancer
48743	B482.00	Malignant neoplasm of body of penis	Any cancer
43392	B483.00	Malignant neoplasm of penis, part unspecified	Any cancer
72127	B484.00	Malignant neoplasm of epididymis	Any cancer
63331	B485.00	Malignant neoplasm of spermatic cord	Any cancer
47767	B486.00	Malignant neoplasm of scrotum	Any cancer
52570	B487.00	Malignant neoplasm, overlapping lesion of penis	Any cancer
67949	B48y.00	Malignant neoplasm of other male genital organ	Any cancer
68161	B48y000	Malignant neoplasm of seminal vesicle	Any cancer
47668	B48y100	Malignant neoplasm of tunica vaginalis	Any cancer
68824	B48y200	Malignant neoplasm, overlapping lesion male genital orgs	Any cancer
92329	B48yz00	Malignant neoplasm of other male genital organ NOS	Any cancer
63224	B48z.00	Malignant neoplasm of penis and other male genital organ NOS	Any cancer
27311	B835.00	Carcinoma in situ of penis	Any cancer
107958	B836.00	Carcinoma in situ other and unspecified male genital organs	Any cancer
8177	B836000	Carcinoma in situ of testis	Any cancer
58879	B836300	Carcinoma in situ of scrotum	Any cancer
109831	B836z00	Carcinoma in situ of male genital organs NOS	Any cancer
7187	B837.00	Carcinoma in situ of bladder	Any cancer
68358	B83z.00	Carcinoma in situ of urinary organs NOS	Any cancer
40671	Byu8.00	[X]Malignant neoplasm of male genital organs	Any cancer
57191	Byu8000	[X]Malignant neoplasm/other specified male genital organs	Any cancer
45262	Byu8200	[X]Malignant neoplasm of male genital organ, unspecified	Any cancer
106003	ByuFC00	[X]Carcinoma in situ of oth+unspecified male genital organs	Any cancer
19415	B0...00	Malignant neoplasm of lip, oral cavity and pharynx	Any cancer
24374	B0...11	Carcinoma of lip, oral cavity and pharynx	Any cancer
14712	B00..00	Malignant neoplasm of lip	Any cancer
9984	B00..11	Carcinoma of lip	Any cancer
73962	B000.00	Malignant neoplasm of upper lip, vermilion border	Any cancer
66270	B000000	Malignant neoplasm of upper lip, external	Any cancer
50296	B000100	Malignant neoplasm of upper lip, lipstick area	Any cancer
98740	B000z00	Malignant neoplasm of upper lip, vermilion border NOS	Any cancer
67446	B001.00	Malignant neoplasm of lower lip, vermilion border	Any cancer
66384	B001000	Malignant neoplasm of lower lip, external	Any cancer
95480	B001100	Malignant neoplasm of lower lip, lipstick area	Any cancer
101707	B001z00	Malignant neoplasm of lower lip, vermilion border NOS	Any cancer
99493	B002.00	Malignant neoplasm of upper lip, inner aspect	Any cancer
99001	B002100	Malignant neoplasm of upper lip, frenulum	Any cancer
98500	B002200	Malignant neoplasm of upper lip, mucosa	Any cancer
90610	B002300	Malignant neoplasm of upper lip, oral aspect	Any cancer
100721	B002z00	Malignant neoplasm of upper lip, inner aspect NOS	Any cancer

Medcode	Readcode	Description	Cancer type
71147	B003.00	Malignant neoplasm of lower lip, inner aspect	Any cancer
67504	B003000	Malignant neoplasm of lower lip, buccal aspect	Any cancer
91843	B003100	Malignant neoplasm of lower lip, frenulum	Any cancer
89909	B003200	Malignant neoplasm of lower lip, mucosa	Any cancer
94441	B003300	Malignant neoplasm of lower lip, oral aspect	Any cancer
96782	B003z00	Malignant neoplasm of lower lip, inner aspect NOS	Any cancer
61692	B004.00	Malignant neoplasm of lip unspecified, inner aspect	Any cancer
73614	B004000	Malignant neoplasm of lip unspecified, buccal aspect	Any cancer
68399	B004200	Malignant neoplasm of lip unspecified, mucosa	Any cancer
100144	B004300	Malignant neoplasm of lip, oral aspect	Any cancer
96783	B005.00	Malignant neoplasm of commissure of lip	Any cancer
18882	B006.00	Malignant neoplasm of overlapping lesion of lip	Any cancer
37553	B007.00	Malignant neoplasm of lip, unspecified	Any cancer
100906	B00z000	Malignant neoplasm of lip, unspecified, external	Any cancer
94251	B00z100	Malignant neoplasm of lip, unspecified, lipstick area	Any cancer
69761	B00zz00	Malignant neoplasm of lip, vermilion border NOS	Any cancer
10283	B01..00	Malignant neoplasm of tongue	Any cancer
43431	B010.00	Malignant neoplasm of base of tongue	Any cancer
69671	B010.11	Malignant neoplasm of posterior third of tongue	Any cancer
34409	B010000	Malignant neoplasm of base of tongue dorsal surface	Any cancer
91035	B010z00	Malignant neoplasm of fixed part of tongue NOS	Any cancer
43642	B011.00	Malignant neoplasm of dorsal surface of tongue	Any cancer
107258	B011100	Malignant neoplasm of midline of tongue	Any cancer
43781	B011z00	Malignant neoplasm of dorsum of tongue NOS	Any cancer
36161	B012.00	Malignant neoplasm of tongue, tip and lateral border	Any cancer
62840	B013.00	Malignant neoplasm of ventral surface of tongue	Any cancer
102142	B013000	Malignant neoplasm of anterior 2/3 of tongue ventral surface	Any cancer
63979	B013100	Malignant neoplasm of frenulum linguae	Any cancer
38488	B013z00	Malignant neoplasm of ventral tongue surface NOS	Any cancer
58121	B014.00	Malignant neoplasm of anterior 2/3 of tongue unspecified	Any cancer
37096	B015.00	Malignant neoplasm of tongue, junctional zone	Any cancer
24852	B016.00	Malignant neoplasm of lingual tonsil	Any cancer
47205	B017.00	Malignant overlapping lesion of tongue	Any cancer
41530	B01y.00	Malignant neoplasm of other sites of tongue	Any cancer
40557	B01z.00	Malignant neoplasm of tongue NOS	Any cancer
20292	B02..00	Malignant neoplasm of major salivary glands	Any cancer
4388	B020.00	Malignant neoplasm of parotid gland	Any cancer
51786	B021.00	Malignant neoplasm of submandibular gland	Any cancer
70928	B022.00	Malignant neoplasm of sublingual gland	Any cancer
70696	B02y.00	Malignant neoplasm of other major salivary glands	Any cancer
50475	B02z.00	Malignant neoplasm of major salivary gland NOS	Any cancer
43400	B03..00	Malignant neoplasm of gum	Any cancer
32024	B030.00	Malignant neoplasm of upper gum	Any cancer
49360	B031.00	Malignant neoplasm of lower gum	Any cancer
101753	B03y.00	Malignant neoplasm of other sites of gum	Any cancer
93218	B03z.00	Malignant neoplasm of gum NOS	Any cancer

Medcode	Readcode	Description	Cancer type
20092	B04..00	Malignant neoplasm of floor of mouth	Any cancer
45408	B040.00	Malignant neoplasm of anterior portion of floor of mouth	Any cancer
45986	B041.00	Malignant neoplasm of lateral portion of floor of mouth	Any cancer
17912	B042.00	Malignant neoplasm, overlapping lesion of floor of mouth	Any cancer
56709	B04y.00	Malignant neoplasm of other sites of floor of mouth	Any cancer
36716	B04z.00	Malignant neoplasm of floor of mouth NOS	Any cancer
14792	B05..00	Malignant neoplasm of other and unspecified parts of mouth	Any cancer
31364	B050.00	Malignant neoplasm of cheek mucosa	Any cancer
30402	B050.11	Malignant neoplasm of buccal mucosa	Any cancer
103796	B051.00	Malignant neoplasm of vestibule of mouth	Any cancer
95772	B051000	Malignant neoplasm of upper buccal sulcus	Any cancer
97530	B051100	Malignant neoplasm of lower buccal sulcus	Any cancer
37590	B052.00	Malignant neoplasm of hard palate	Any cancer
40292	B053.00	Malignant neoplasm of soft palate	Any cancer
37516	B054.00	Malignant neoplasm of uvula	Any cancer
70819	B055.00	Malignant neoplasm of palate unspecified	Any cancer
96003	B055000	Malignant neoplasm of junction of hard and soft palate	Any cancer
69951	B055100	Malignant neoplasm of roof of mouth	Any cancer
28559	B055z00	Malignant neoplasm of palate NOS	Any cancer
37724	B056.00	Malignant neoplasm of retromolar area	Any cancer
10314	B057.00	Overlapping lesion of other and unspecified parts of mouth	Any cancer
37916	B05y.00	Malignant neoplasm of other specified mouth parts	Any cancer
55015	B05z.00	Malignant neoplasm of mouth NOS	Any cancer
37549	B05z000	Kaposi's sarcoma of palate	Any cancer
22893	B06..00	Malignant neoplasm of oropharynx	Any cancer
16241	B060.00	Malignant neoplasm of tonsil	Any cancer
26448	B060000	Malignant neoplasm of faucial tonsil	Any cancer
101988	B060100	Malignant neoplasm of palatine tonsil	Any cancer
102151	B060200	Malignant neoplasm of overlapping lesion of tonsil	Any cancer
53884	B060z00	Malignant neoplasm tonsil NOS	Any cancer
24397	B061.00	Malignant neoplasm of tonsillar fossa	Any cancer
55066	B062.00	Malignant neoplasm of tonsillar pillar	Any cancer
51926	B062000	Malignant neoplasm of faucial pillar	Any cancer
99185	B062100	Malignant neoplasm of glossopalatine fold	Any cancer
61510	B062200	Malignant neoplasm of palatoglossal arch	Any cancer
93842	B062300	Malignant neoplasm of palatopharyngeal arch	Any cancer
100002	B062z00	Malignant neoplasm of tonsillar fossa NOS	Any cancer
39554	B063.00	Malignant neoplasm of vallecula	Any cancer
46728	B064.00	Malignant neoplasm of anterior epiglottis	Any cancer
26134	B064000	Malignant neoplasm of epiglottis, free border	Any cancer
91895	B064100	Malignant neoplasm of glossoepiglottic fold	Any cancer
73439	B064z00	Malignant neoplasm of anterior epiglottis NOS	Any cancer
48519	B065.00	Malignant neoplasm of junctional region of epiglottis	Any cancer
56355	B066.00	Malignant neoplasm of lateral wall of oropharynx	Any cancer
90124	B067.00	Malignant neoplasm of posterior wall of oropharynx	Any cancer
67323	B06y.00	Malignant neoplasm of oropharynx, other specified sites	Any cancer

Medcode	Readcode	Description	Cancer type
91037	B06yz00	Malignant neoplasm of other specified site of oropharynx NOS	Any cancer
43200	B06z.00	Malignant neoplasm of oropharynx NOS	Any cancer
24675	B07..00	Malignant neoplasm of nasopharynx	Any cancer
94390	B070.00	Malignant neoplasm of roof of nasopharynx	Any cancer
95429	B071.00	Malignant neoplasm of posterior wall of nasopharynx	Any cancer
33388	B071000	Malignant neoplasm of adenoid	Any cancer
46548	B071100	Malignant neoplasm of pharyngeal tonsil	Any cancer
96869	B071z00	Malignant neoplasm of posterior wall of nasopharynx NOS	Any cancer
59004	B072.00	Malignant neoplasm of lateral wall of nasopharynx	Any cancer
37940	B072000	Malignant neoplasm of pharyngeal recess	Any cancer
102205	B072z00	Malignant neoplasm of lateral wall of nasopharynx NOS	Any cancer
44139	B073.00	Malignant neoplasm of anterior wall of nasopharynx	Any cancer
106915	B073100	Malignant neoplasm of nasopharyngeal soft palate surface	Any cancer
99386	B073200	Malignant neoplasm posterior margin nasal septum and choanae	Any cancer
100918	B073z00	Malignant neoplasm of anterior wall of nasopharynx NOS	Any cancer
66422	B074.00	Malignant neoplasm, overlapping lesion of nasopharynx	Any cancer
55630	B07y.00	Malignant neoplasm of other specified site of nasopharynx	Any cancer
28665	B07z.00	Malignant neoplasm of nasopharynx NOS	Any cancer
34012	B08..00	Malignant neoplasm of hypopharynx	Any cancer
43548	B080.00	Malignant neoplasm of postcricoid region	Any cancer
39897	B081.00	Malignant neoplasm of pyriform sinus	Any cancer
57248	B082.00	Malignant neoplasm aryepiglottic fold, hypopharyngeal aspect	Any cancer
64462	B083.00	Malignant neoplasm of posterior pharynx	Any cancer
88362	B08y.00	Malignant neoplasm of other specified hypopharyngeal site	Any cancer
28451	B08z.00	Malignant neoplasm of hypopharynx NOS	Any cancer
46114	B0z..00	Malig neop other/ill-defined sites lip, oral cavity, pharynx	Any cancer
16297	B0z0.00	Malignant neoplasm of pharynx unspecified	Any cancer
95016	B0z1.00	Malignant neoplasm of Waldeyer's ring	Any cancer
39084	B0z2.00	Malignant neoplasm of laryngopharynx	Any cancer
49758	B0zy.00	Malignant neoplasm of other sites lip, oral cavity, pharynx	Any cancer
39430	B0zz.00	Malignant neoplasm of lip, oral cavity and pharynx NOS	Any cancer
23389	B200.00	Malignant neoplasm of nasal cavities	Any cancer
71204	B200000	Malignant neoplasm of cartilage of nose	Any cancer
98911	B200100	Malignant neoplasm of nasal conchae	Any cancer
62761	B200200	Malignant neoplasm of septum of nose	Any cancer
62182	B200300	Malignant neoplasm of vestibule of nose	Any cancer
42856	B200z00	Malignant neoplasm of nasal cavities NOS	Any cancer
24456	B201.00	Malig neop auditory tube, middle ear and mastoid air cells	Any cancer
107916	B201000	Malignant neoplasm of auditory (Eustachian) tube	Any cancer
98537	B201100	Malignant neoplasm of tympanic cavity	Any cancer
54613	B201200	Malignant neoplasm of tympanic antrum	Any cancer
71946	B201300	Malignant neoplasm of mastoid air cells	Any cancer
73537	B201z00	Malig neop auditory tube, middle ear, mastoid air cells NOS	Any cancer
32174	B202.00	Malignant neoplasm of maxillary sinus	Any cancer
54636	B203.00	Malignant neoplasm of ethmoid sinus	Any cancer
15684	B204.00	Malignant neoplasm of frontal sinus	Any cancer

Medcode	Readcode	Description	Cancer type
65215	B205.00	Malignant neoplasm of sphenoidal sinus	Any cancer
39590	B206.00	Malignant neoplasm, overlapping lesion of accessory sinuses	Any cancer
96971	B20y.00	Malig neop other site nasal cavity, middle ear and sinuses	Any cancer
55246	B20z.00	Malignant neoplasm of accessory sinus NOS	Any cancer
95390	B800.00	Carcinoma in situ of lip, oral cavity and pharynx	Any cancer
37505	B800.11	Carcinoma in situ of oral cavity	Any cancer
42129	B800.12	Carcinoma in situ of pharynx	Any cancer
47737	B800000	Carcinoma in situ of lip	Any cancer
27944	B800100	Carcinoma in situ of tongue	Any cancer
50288	B800200	Carcinoma in situ of salivary glands	Any cancer
57866	B800300	Carcinoma in situ of gums	Any cancer
24801	B800400	Carcinoma in situ of floor of mouth	Any cancer
34823	B800500	Carcinoma in situ of cheek	Any cancer
30966	B800600	Carcinoma in situ of palate	Any cancer
36104	B800700	Carcinoma in situ of nasopharynx	Any cancer
50419	B800800	Carcinoma in situ of oropharynx	Any cancer
44663	B800900	Carcinoma in situ of hypopharynx	Any cancer
37187	B800z00	Carcinoma in situ of lip, oral cavity and pharynx NOS	Any cancer
58973	Byu0.00	[X]Malignant neoplasm of lip, oral cavity and pharynx	Any cancer
102314	1427000	H/O: prostate cancer	Any cancer
780	B46..00	Malignant neoplasm of prostate	Any cancer
6328	B834.00	Carcinoma in situ of prostate	Any cancer
54599	B834000	High grade prostatic intraepithelial neoplasia	Any cancer
105236	B834100	Prostatic intraepithelial neoplasia	Any cancer
37306	ZV10415	[V]Personal history of malignant neoplasm of prostate	Any cancer
319	B21..00	Malignant neoplasm of larynx	Any cancer
318	B210.00	Malignant neoplasm of glottis	Any cancer
26165	B211.00	Malignant neoplasm of supraglottis	Any cancer
22441	B212.00	Malignant neoplasm of subglottis	Any cancer
43111	B213.00	Malignant neoplasm of laryngeal cartilage	Any cancer
63460	B213000	Malignant neoplasm of arytenoid cartilage	Any cancer
37805	B213100	Malignant neoplasm of cricoid cartilage	Any cancer
107878	B213200	Malignant neoplasm of cuneiform cartilage	Any cancer
47862	B213300	Malignant neoplasm of thyroid cartilage	Any cancer
97332	B213z00	Malignant neoplasm of laryngeal cartilage NOS	Any cancer
50579	B214.00	Malignant neoplasm, overlapping lesion of larynx	Any cancer
55374	B215.00	Malignant neoplasm of epiglottis NOS	Any cancer
26813	B21y.00	Malignant neoplasm of larynx, other specified site	Any cancer
9237	B21z.00	Malignant neoplasm of larynx NOS	Any cancer
13243	B22..00	Malignant neoplasm of trachea, bronchus and lung	Any cancer
15221	B220.00	Malignant neoplasm of trachea	Any cancer
103946	B220100	Malignant neoplasm of mucosa of trachea	Any cancer
37810	B220z00	Malignant neoplasm of trachea NOS	Any cancer
12870	B221.00	Malignant neoplasm of main bronchus	Any cancer
17391	B221000	Malignant neoplasm of carina of bronchus	Any cancer
33444	B221100	Malignant neoplasm of hilus of lung	Any cancer

Medcode	Readcode	Description	Cancer type
21698	B221z00	Malignant neoplasm of main bronchus NOS	Any cancer
10358	B222.00	Malignant neoplasm of upper lobe, bronchus or lung	Any cancer
20170	B222.11	Pancoast's syndrome	Any cancer
31700	B222000	Malignant neoplasm of upper lobe bronchus	Any cancer
25886	B222100	Malignant neoplasm of upper lobe of lung	Any cancer
44169	B222z00	Malignant neoplasm of upper lobe, bronchus or lung NOS	Any cancer
31268	B223.00	Malignant neoplasm of middle lobe, bronchus or lung	Any cancer
41523	B223000	Malignant neoplasm of middle lobe bronchus	Any cancer
39923	B223100	Malignant neoplasm of middle lobe of lung	Any cancer
54134	B223z00	Malignant neoplasm of middle lobe, bronchus or lung NOS	Any cancer
31188	B224.00	Malignant neoplasm of lower lobe, bronchus or lung	Any cancer
18678	B224000	Malignant neoplasm of lower lobe bronchus	Any cancer
12582	B224100	Malignant neoplasm of lower lobe of lung	Any cancer
42566	B224z00	Malignant neoplasm of lower lobe, bronchus or lung NOS	Any cancer
36371	B225.00	Malignant neoplasm of overlapping lesion of bronchus & lung	Any cancer
7484	B226.00	Mesothelioma	Any cancer
38961	B22y.00	Malignant neoplasm of other sites of bronchus or lung	Any cancer
3903	B22z.00	Malignant neoplasm of bronchus or lung NOS	Any cancer
2587	B22z.11	Lung cancer	Any cancer
31573	B23..00	Malignant neoplasm of pleura	Any cancer
67107	B230.00	Malignant neoplasm of parietal pleura	Any cancer
106194	B231.00	Malignant neoplasm of visceral pleura	Any cancer
9600	B232.00	Mesothelioma of pleura	Any cancer
98104	B23y.00	Malignant neoplasm of other specified pleura	Any cancer
34742	B23z.00	Malignant neoplasm of pleura NOS	Any cancer
64050	B81..00	Carcinoma in situ of respiratory system	Any cancer
11403	B810.00	Carcinoma in situ of larynx	Any cancer
35772	B810000	Carcinoma in situ of thyroid cartilage	Any cancer
36948	B810100	Carcinoma in situ of cricoid cartilage	Any cancer
53460	B810200	Carcinoma in situ of epiglottis	Any cancer
65953	B810300	Carcinoma in situ of arytenoid cartilage	Any cancer
31860	B810600	Carcinoma in situ of aryepiglottic fold	Any cancer
73076	B810700	Carcinoma in situ of vestibular fold	Any cancer
7697	B810800	Carcinoma in situ of vocal fold-glottis	Any cancer
10375	B810811	Carcinoma in situ of glottis	Any cancer
53882	B810z00	Carcinoma in situ of larynx NOS	Any cancer
51714	B811.00	Carcinoma in situ of trachea	Any cancer
9267	B812.00	Carcinoma in situ of bronchus and lung	Any cancer
49159	B812000	Carcinoma in situ of carina of bronchus	Any cancer
35058	B812100	Carcinoma in situ of main bronchus	Any cancer
37579	B812200	Carcinoma in situ of upper lobe bronchus and lung	Any cancer
47897	B812300	Carcinoma in situ of middle lobe bronchus and lung	Any cancer
52373	B812400	Carcinoma in situ of lower lobe bronchus and lung	Any cancer
25372	B812z00	Carcinoma in situ of bronchus or lung NOS	Any cancer
97954	B81y.00	Carcinoma in situ of other specified part respiratory system	Any cancer
59426	B81y.11	Carcinoma in situ of nasal sinuses	Any cancer

Medcode	Readcode	Description	Cancer type
46497	B81y000	Carcinoma in situ of pleura	Any cancer
95559	B81yz00	Carcinoma in situ of specified parts respiratory system NOS	Any cancer
62610	B81z.00	Carcinoma in situ of respiratory organ NOS	Any cancer
35325	Byu2.00	[X]Malignant neoplasm of respiratory and intrathoracic orga	Any cancer
40595	Byu2000	[X]Malignant neoplasm of bronchus or lung, unspecified	Any cancer
21715	Byu5011	[X]Mesothelioma of lung	Any cancer
100781	ByuF300	[X]Carcinoma in situ of other parts of respiratory system	Any cancer
4632	B33..00	Other malignant neoplasm of skin	Any cancer
876	B33..11	Basal cell carcinoma	Any cancer
5034	B33..12	Epithelioma	Any cancer
1940	B33..13	Rodent ulcer	Any cancer
37016	B33..14	Malignant neoplasm of sebaceous gland	Any cancer
40443	B33..15	Malignant neoplasm of sweat gland	Any cancer
3445	B33..16	Epithelioma basal cell	Any cancer
18245	B330.00	Malignant neoplasm of skin of lip	Any cancer
43087	B331.00	Malignant neoplasm of eyelid including canthus	Any cancer
36731	B331000	Malignant neoplasm of canthus	Any cancer
55550	B331100	Malignant neoplasm of upper eyelid	Any cancer
41958	B331200	Malignant neoplasm of lower eyelid	Any cancer
53515	B332.00	Malignant neoplasm skin of ear and external auricular canal	Any cancer
33997	B332000	Malignant neoplasm of skin of auricle (ear)	Any cancer
62080	B332100	Malignant neoplasm of skin of external auditory meatus	Any cancer
33271	B332200	Malignant neoplasm of pinna NEC	Any cancer
62399	B332z00	Malig neop skin of ear and external auricular canal NOS	Any cancer
27370	B333.00	Malignant neoplasm skin of other and unspecified parts face	Any cancer
30645	B333000	Malignant neoplasm of skin of cheek, external	Any cancer
49403	B333100	Malignant neoplasm of skin of chin	Any cancer
55670	B333200	Malignant neoplasm of skin of eyebrow	Any cancer
30576	B333300	Malignant neoplasm of skin of forehead	Any cancer
16202	B333400	Malignant neoplasm of skin of nose (external)	Any cancer
21327	B333500	Malignant neoplasm of skin of temple	Any cancer
46008	B333z00	Malignant neoplasm skin other and unspec part of face NOS	Any cancer
54234	B334.00	Malignant neoplasm of scalp and skin of neck	Any cancer
37165	B334000	Malignant neoplasm of scalp	Any cancer
43619	B334100	Malignant neoplasm of skin of neck	Any cancer
73760	B334z00	Malignant neoplasm of scalp or skin of neck NOS	Any cancer
57446	B335.00	Malignant neoplasm of skin of trunk, excluding scrotum	Any cancer
70380	B335000	Malignant neoplasm of skin of axillary fold	Any cancer
37969	B335100	Malignant neoplasm of skin of chest, excluding breast	Any cancer
30543	B335200	Malignant neoplasm of skin of breast	Any cancer
18618	B335300	Malignant neoplasm of skin of abdominal wall	Any cancer
67748	B335400	Malignant neoplasm of skin of umbilicus	Any cancer
66319	B335500	Malignant neoplasm of skin of groin	Any cancer
46458	B335600	Malignant neoplasm of skin of perineum	Any cancer
45077	B335700	Malignant neoplasm of skin of back	Any cancer
62305	B335800	Malignant neoplasm of skin of buttock	Any cancer

Medcode	Readcode	Description	Cancer type
23480	B335900	Malignant neoplasm of perianal skin	Any cancer
66447	B335A00	Malignant neoplasm of skin of scapular region	Any cancer
15868	B335z00	Malignant neoplasm of skin of trunk, excluding scrotum, NOS	Any cancer
30747	B336.00	Malignant neoplasm of skin of upper limb and shoulder	Any cancer
43122	B336000	Malignant neoplasm of skin of shoulder	Any cancer
42707	B336100	Malignant neoplasm of skin of upper arm	Any cancer
30577	B336200	Malignant neoplasm of skin of fore-arm	Any cancer
54352	B336300	Malignant neoplasm of skin of hand	Any cancer
25245	B336400	Malignant neoplasm of skin of finger	Any cancer
64406	B336500	Malignant neoplasm of skin of thumb	Any cancer
60526	B336z00	Malignant neoplasm of skin of upper limb or shoulder NOS	Any cancer
57442	B337.00	Malignant neoplasm of skin of lower limb and hip	Any cancer
70988	B337000	Malignant neoplasm of skin of hip	Any cancer
58601	B337100	Malignant neoplasm of skin of thigh	Any cancer
56954	B337200	Malignant neoplasm of skin of knee	Any cancer
68197	B337300	Malignant neoplasm of skin of popliteal fossa area	Any cancer
33682	B337400	Malignant neoplasm of skin of lower leg	Any cancer
64270	B337500	Malignant neoplasm of skin of ankle	Any cancer
104025	B337600	Malignant neoplasm of skin of heel	Any cancer
70587	B337700	Malignant neoplasm of skin of foot	Any cancer
65782	B337800	Malignant neoplasm of skin of toe	Any cancer
67914	B337900	Malignant neoplasm of skin of great toe	Any cancer
61194	B337z00	Malignant neoplasm of skin of lower limb or hip NOS	Any cancer
93352	B338.00	Squamous cell carcinoma of skin	Any cancer
24375	B339.00	Dermatofibrosarcoma protuberans	Any cancer
42429	B33X.00	Malignant neoplasm overlapping lesion of skin	Any cancer
18354	B33y.00	Malignant neoplasm of other specified skin sites	Any cancer
2492	B33z.00	Malignant neoplasm of skin NOS	Any cancer
93490	B33z.11	Squamous cell carcinoma of skin NOS	Any cancer
12084	B82..00	Carcinoma in situ of skin	Any cancer
63957	B820.00	Carcinoma in situ of skin of lip	Any cancer
57550	B821.00	Carcinoma in situ of skin of eyelid including canthus	Any cancer
50189	B822.00	Carcinoma in situ skin of ear and external auricular canal	Any cancer
32249	B822.11	Carcinoma in situ of ear	Any cancer
59614	B822000	Carcinoma in situ of skin of auricle	Any cancer
70295	B822z00	Carcinoma in situ skin of ear/external auricular canal NOS	Any cancer
49254	B823.00	Carcinoma in situ of skin of other parts of face	Any cancer
47789	B823000	Carcinoma in situ of skin of forehead skin	Any cancer
69720	B823100	Carcinoma in situ of skin of eyebrow	Any cancer
61103	B823300	Carcinoma in situ of skin of cheek	Any cancer
3135	B823400	Carcinoma in situ of skin of nose	Any cancer
31511	B823500	Carcinoma in situ of skin of temple	Any cancer
65222	B823600	Carcinoma in situ of skin of jaw	Any cancer
110614	B823z00	Carcinoma in situ of skin of other parts of face NOS	Any cancer
69345	B824.00	Carcinoma in situ of scalp and skin of neck	Any cancer
19665	B824000	Carcinoma in situ of scalp	Any cancer

Medcode	Readcode	Description	Cancer type
54140	B824100	Carcinoma in situ of skin of neck	Any cancer
52328	B825.00	Carcinoma in situ of skin of trunk, excluding scrotum	Any cancer
8647	B825000	Carcinoma in situ of skin of breast	Any cancer
62939	B825100	Carcinoma in situ of skin of chest wall NOS	Any cancer
39390	B825200	Carcinoma in situ of skin of axilla	Any cancer
38032	B825300	Carcinoma in situ of skin of back	Any cancer
42212	B825400	Carcinoma in situ of skin of abdominal wall	Any cancer
57358	B825500	Carcinoma in situ of skin of groin	Any cancer
38777	B825600	Carcinoma in situ of skin of perineum	Any cancer
61321	B825700	Carcinoma in situ of skin of buttock	Any cancer
56374	B825800	Carcinoma in situ of perianal skin	Any cancer
60563	B825z00	Carcinoma in situ of skin of trunk NOS	Any cancer
46568	B826.00	Carcinoma in situ of skin of upper limb and shoulder	Any cancer
56554	B826000	Carcinoma in situ of skin of shoulder	Any cancer
57284	B826100	Carcinoma in situ of skin of upper arm	Any cancer
54790	B826200	Carcinoma in situ of skin of lower arm	Any cancer
49358	B826300	Carcinoma in situ of skin of hand	Any cancer
90339	B826z00	Carcinoma in situ of skin of upper limb or shoulder NOS	Any cancer
14815	B827.00	Carcinoma in situ of skin of lower limb and hip	Any cancer
708	B827.11	Carcinoma in situ of skin of leg	Any cancer
71655	B827000	Carcinoma in situ of skin of hip	Any cancer
46469	B827100	Carcinoma in situ of skin of thigh	Any cancer
69601	B827200	Carcinoma in situ of skin of knee	Any cancer
27542	B827300	Carcinoma in situ of skin of lower leg	Any cancer
67755	B827400	Carcinoma in situ of skin of foot	Any cancer
64630	B827z00	Carcinoma in situ of skin of lower limb or hip NOS	Any cancer
779	B49..00	Malignant neoplasm of urinary bladder	Any cancer
38862	B490.00	Malignant neoplasm of trigone of urinary bladder	Any cancer
44996	B491.00	Malignant neoplasm of dome of urinary bladder	Any cancer
35963	B492.00	Malignant neoplasm of lateral wall of urinary bladder	Any cancer
19162	B493.00	Malignant neoplasm of anterior wall of urinary bladder	Any cancer
42012	B494.00	Malignant neoplasm of posterior wall of urinary bladder	Any cancer
41571	B495.00	Malignant neoplasm of bladder neck	Any cancer
28241	B496.00	Malignant neoplasm of ureteric orifice	Any cancer
42023	B497.00	Malignant neoplasm of urachus	Any cancer
105388	B498.00	Local recurrence of malignant tumour of urinary bladder	Any cancer
36949	B49y.00	Malignant neoplasm of other site of urinary bladder	Any cancer
47801	B49y000	Malignant neoplasm, overlapping lesion of bladder	Any cancer
31102	B49z.00	Malignant neoplasm of urinary bladder NOS	Any cancer
13559	B4A..00	Malig neop of kidney and other unspecified urinary organs	Any cancer
18712	B4A..11	Renal malignant neoplasm	Any cancer
1599	B4A0.00	Malignant neoplasm of kidney parenchyma	Any cancer
7978	B4A0000	Hypernephroma	Any cancer
12389	B4A1.00	Malignant neoplasm of renal pelvis	Any cancer
27540	B4A1000	Malignant neoplasm of renal calyces	Any cancer
101608	B4A1100	Malignant neoplasm of ureteropelvic junction	Any cancer

Medcode	Readcode	Description	Cancer type
54184	B4A1z00	Malignant neoplasm of renal pelvis NOS	Any cancer
15223	B4A2.00	Malignant neoplasm of ureter	Any cancer
15644	B4A3.00	Malignant neoplasm of urethra	Any cancer
72174	B4A4.00	Malignant neoplasm of paraurethral glands	Any cancer
44884	B4Ay.00	Malignant neoplasm of other urinary organs	Any cancer
59286	B4Ay000	Malignant neoplasm of overlapping lesion of urinary organs	Any cancer
29462	B4Az.00	Malignant neoplasm of kidney or urinary organs NOS	Any cancer
38931	B4y..00	Malignant neoplasm of genitourinary organ OS	Any cancer
52594	B4z..00	Malignant neoplasm of genitourinary organ NOS	Any cancer
35113	Byu9.00	[X]Malignant neoplasm of urinary tract	Any cancer
45260	Byu9000	[X]Malignant neoplasm of urinary organ, unspecified	Any cancer
865	B32..00	Malignant melanoma of skin	Any cancer
70637	B320.00	Malignant melanoma of lip	Any cancer
54632	B321.00	Malignant melanoma of eyelid including canthus	Any cancer
57260	B322.00	Malignant melanoma of ear and external auricular canal	Any cancer
59061	B322000	Malignant melanoma of auricle (ear)	Any cancer
102145	B322100	Malignant melanoma of external auditory meatus	Any cancer
73744	B322z00	Malignant melanoma of ear and external auricular canal NOS	Any cancer
47252	B323.00	Malignant melanoma of other and unspecified parts of face	Any cancer
41278	B323000	Malignant melanoma of external surface of cheek	Any cancer
71136	B323100	Malignant melanoma of chin	Any cancer
47094	B323200	Malignant melanoma of eyebrow	Any cancer
68133	B323300	Malignant melanoma of forehead	Any cancer
45139	B323400	Malignant melanoma of external surface of nose	Any cancer
58958	B323500	Malignant melanoma of temple	Any cancer
67806	B323z00	Malignant melanoma of face NOS	Any cancer
65625	B324.00	Malignant melanoma of scalp and neck	Any cancer
55881	B324000	Malignant melanoma of scalp	Any cancer
45306	B324100	Malignant melanoma of neck	Any cancer
99257	B324z00	Malignant melanoma of scalp and neck NOS	Any cancer
38689	B325.00	Malignant melanoma of trunk (excluding scrotum)	Any cancer
49814	B325000	Malignant melanoma of axilla	Any cancer
32768	B325100	Malignant melanoma of breast	Any cancer
53629	B325200	Malignant melanoma of buttock	Any cancer
34259	B325300	Malignant melanoma of groin	Any cancer
109002	B325400	Malignant melanoma of perianal skin	Any cancer
95629	B325500	Malignant melanoma of perineum	Any cancer
43715	B325600	Malignant melanoma of umbilicus	Any cancer
43463	B325700	Malignant melanoma of back	Any cancer
51209	B325800	Malignant melanoma of chest wall	Any cancer
45760	B325z00	Malignant melanoma of trunk, excluding scrotum, NOS	Any cancer
65164	B326.00	Malignant melanoma of upper limb and shoulder	Any cancer
50505	B326000	Malignant melanoma of shoulder	Any cancer
54685	B326100	Malignant melanoma of upper arm	Any cancer
45755	B326200	Malignant melanoma of fore-arm	Any cancer
62475	B326300	Malignant melanoma of hand	Any cancer

Medcode	Readcode	Description	Cancer type
25602	B326400	Malignant melanoma of finger	Any cancer
63997	B326500	Malignant melanoma of thumb	Any cancer
55292	B326z00	Malignant melanoma of upper limb or shoulder NOS	Any cancer
46255	B327.00	Malignant melanoma of lower limb and hip	Any cancer
73536	B327000	Malignant melanoma of hip	Any cancer
51873	B327100	Malignant melanoma of thigh	Any cancer
54305	B327200	Malignant melanoma of knee	Any cancer
39878	B327300	Malignant melanoma of popliteal fossa area	Any cancer
37872	B327400	Malignant melanoma of lower leg	Any cancer
42714	B327500	Malignant melanoma of ankle	Any cancer
61246	B327600	Malignant melanoma of heel	Any cancer
41490	B327700	Malignant melanoma of foot	Any cancer
36899	B327800	Malignant melanoma of toe	Any cancer
53369	B327900	Malignant melanoma of great toe	Any cancer
64327	B327z00	Malignant melanoma of lower limb or hip NOS	Any cancer
42153	B32y.00	Malignant melanoma of other specified skin site	Any cancer
96585	B32y000	Overlapping malignant melanoma of skin	Any cancer
28556	B32z.00	Malignant melanoma of skin NOS	Any cancer
19686	B828.00	Melanoma in situ of skin	Any cancer
46536	B828000	Melanoma in situ of lip	Any cancer
37108	B828100	Melanoma in situ of eyelid, including canthus	Any cancer
72032	B828200	Melanoma in situ of ear and external auricular canal	Any cancer
97858	B828300	Melanoma in situ of scalp and neck	Any cancer
59768	B828400	Melanoma in situ of trunk	Any cancer
56694	B828500	Melanoma in situ of upper limb, including shoulder	Any cancer
47850	B828600	Melanoma in situ of lower limb, including hip	Any cancer
49572	B828700	Melanoma in situ of scalp	Any cancer
52332	B828800	Melanoma in situ of back of hand	Any cancer
71044	B828900	Melanoma in situ of back	Any cancer
54246	B828W00	Melanoma in situ, unspecified	Any cancer
61989	B828X00	Melanoma in situ of other and unspecified parts of face	Any cancer
56925	Byu4000	[X]Malignant melanoma of other+unspecified parts of face	Any cancer
19444	Byu4100	[X]Malignant melanoma of skin, unspecified	Any cancer
97628	ByuF600	[X]Melanoma in situ of other sites	Any cancer
73261	ByuFF00	[X]Melanoma in situ, unspecified	Any cancer
87335	B624.12	Hairy cell leukaemia	Any cancer
37182	B63..00	Multiple myeloma and immunoproliferative neoplasms	Any cancer
4944	B630.00	Multiple myeloma	Any cancer
43552	B630.11	Kahler's disease	Any cancer
15211	B630.12	Myelomatosis	Any cancer
22158	B630000	Malignant plasma cell neoplasm, extramedullary plasmacytoma	Any cancer
19028	B630100	Solitary myeloma	Any cancer
21329	B630200	Plasmacytoma NOS	Any cancer
46042	B630300	Lambda light chain myeloma	Any cancer
104418	B630400	Solitary plasmacytoma	Any cancer
39187	B631.00	Plasma cell leukaemia	Any cancer

Medcode	Readcode	Description	Cancer type
64567	B63y.00	Other immunoproliferative neoplasms	Any cancer
43450	B63z.00	Immunoproliferative neoplasm or myeloma NOS	Any cancer
19372	B64..00	Lymphoid leukaemia	Any cancer
4222	B64..11	Lymphatic leukaemia	Any cancer
4251	B640.00	Acute lymphoid leukaemia	Any cancer
104325	B640000	B-cell acute lymphoblastic leukaemia	Any cancer
8625	B641.00	Chronic lymphoid leukaemia	Any cancer
27790	B641.11	Chronic lymphatic leukaemia	Any cancer
104328	B641000	B-cell chronic lymphocytic leukaemia	Any cancer
107017	B641011	Chronic lymphocytic leukaemia of B-cell type	Any cancer
107052	B641100	Clinical stage A chronic lymphocytic leukaemia	Any cancer
106924	B641200	Clinical stage B chronic lymphocytic leukaemia	Any cancer
107163	B641300	Clinical stage C chronic lymphocytic leukaemia	Any cancer
72774	B642.00	Subacute lymphoid leukaemia	Any cancer
49725	B64y.00	Other lymphoid leukaemia	Any cancer
31586	B64y100	Prolymphocytic leukaemia	Any cancer
37461	B64y200	Adult T-cell leukaemia	Any cancer
108656	B64y300	B-cell prolymphocytic leukaemia	Any cancer
107643	B64y400	T-cell prolymphocytic leukaemia	Any cancer
104939	B64y500	Adult T-cell lymphoma/leukaemia (HTLV-1-associated)	Any cancer
38331	B64yz00	Other lymphoid leukaemia NOS	Any cancer
38914	B64z.00	Lymphoid leukaemia NOS	Any cancer
7176	B65..00	Myeloid leukaemia	Any cancer
4413	B650.00	Acute myeloid leukaemia	Any cancer
10726	B651.00	Chronic myeloid leukaemia	Any cancer
31701	B651.11	Chronic granulocytic leukaemia	Any cancer
100786	B651000	Chronic eosinophilic leukaemia	Any cancer
105957	B651100	Chronic myeloid leukaemia, BCR/ABL positive	Any cancer
102783	B651200	Chronic neutrophilic leukaemia	Any cancer
107236	B651300	Atypical chronic myeloid leukaemia, BCR/ABL negative	Any cancer
27520	B651z00	Chronic myeloid leukaemia NOS	Any cancer
63475	B652.00	Subacute myeloid leukaemia	Any cancer
70724	B653.00	Myeloid sarcoma	Any cancer
52327	B653000	Chloroma	Any cancer
39629	B653100	Granulocytic sarcoma	Any cancer
104788	B654.00	Acute myeloblastic leukaemia	Any cancer
27664	B65y100	Acute promyelocytic leukaemia	Any cancer
66089	B65yz00	Other myeloid leukaemia NOS	Any cancer
33344	B65z.00	Myeloid leukaemia NOS	Any cancer
35875	B66..00	Monocytic leukaemia	Any cancer
108715	B66..11	Histiocytic leukaemia	Any cancer
67700	B66..12	Monoblastic leukaemia	Any cancer
19974	B660.00	Acute monocytic leukaemia	Any cancer
27458	B661.00	Chronic monocytic leukaemia	Any cancer
101606	B662.00	Subacute monocytic leukaemia	Any cancer
108424	B663.00	Acute monoblastic leukaemia	Any cancer

Medcode	Readcode	Description	Cancer type
99015	B66y.00	Other monocytic leukaemia	Any cancer
103645	B66yz00	Other monocytic leukaemia NOS	Any cancer
93342	B66z.00	Monocytic leukaemia NOS	Any cancer
37272	B67..00	Other specified leukaemia	Any cancer
42539	B670.00	Acute erythraemia and erythroleukaemia	Any cancer
27340	B670.11	Di Guglielmo's disease	Any cancer
37468	B671.00	Chronic erythraemia	Any cancer
63653	B671.11	Heilmeyer-Schoner disease	Any cancer
57671	B672.00	Megakaryocytic leukaemia	Any cancer
65777	B672.11	Thrombocytic leukaemia	Any cancer
65721	B673.00	Mast cell leukaemia	Any cancer
50858	B674.00	Acute panmyelosis	Any cancer
28276	B675.00	Acute myelofibrosis	Any cancer
104273	B677.00	Myelodysplastic and myeloproliferative disease	Any cancer
94174	B67y.00	Other and unspecified leukaemia	Any cancer
72197	B67y000	Lymphosarcoma cell leukaemia	Any cancer
99413	B67yz00	Other and unspecified leukaemia NOS	Any cancer
30632	B67z.00	Other specified leukaemia NOS	Any cancer
25191	B68..00	Leukaemia of unspecified cell type	Any cancer
4072	B680.00	Acute leukaemia NOS	Any cancer
16416	B681.00	Chronic leukaemia NOS	Any cancer
54793	B682.00	Subacute leukaemia NOS	Any cancer
34692	B68y.00	Other leukaemia of unspecified cell type	Any cancer
4250	B68z.00	Leukaemia NOS	Any cancer
20440	B69..00	Myelomonocytic leukaemia	Any cancer
61500	B690.00	Acute myelomonocytic leukaemia	Any cancer
22050	B691.00	Chronic myelomonocytic leukaemia	Any cancer
104475	B692.00	Subacute myelomonocytic leukaemia	Any cancer
105069	B693.00	Juvenile myelomonocytic leukaemia	Any cancer
30646	B6y..00	Malignant neoplasm lymphatic or haematopoietic tissue OS	Any cancer
6115	B6y0.00	Myeloproliferative disorder	Any cancer
17056	B6y0.11	Myeloproliferative disease	Any cancer
39336	B6y1.00	Myelosclerosis with myeloid metaplasia	Any cancer
110065	B6y1.12	Osteomyelofibrosis	Any cancer
67029	ByuD500	[X]Other lymphoid leukaemia	Any cancer
61693	ByuD600	[X]Other myeloid leukaemia	Any cancer
89762	ByuD700	[X]Other monocytic leukaemia	Any cancer
89329	ByuD800	[X]Other specified leukaemias	Any cancer
65165	ByuD900	[X]Other leukaemia of unspecified cell type	Any cancer
