# Supplementary material for: Increasing cancer risk over calendar year in people with multiple sclerosis: a case–control study
Source: J Neurol. 2020 Oct 21;268(3):817–24. doi: 10.1007/s00415-020-10170-5 (PMC7914231; doi:10.1007/s00415-020-10170-5)
Supplement: Supplementary file 2 — Supplementary file2 (PDF 5 kb) [file 415_2020_10170_MOESM2_ESM.pdf]

Supplementary Table 2 Frequency of cancer specific read codes among MS patients and matched controls after index date

Cancer type	MS patients n (%)	Matched controls n (%)
Hodgkin lymphoma	2 (0.02)	13 (0.03)
Non-Hodgkin lymphoma	15 (0.15)	64 (0.16)
Leukaemia	11 (0.11)	72 (0.18)
Brain cancer	4 (0.039)	14 (0.04)
Eye cancer	0 (0)	2 (0.01)
Ear-nose-throat cancer	5 (0.05)	31 (0.08)
Lung and respiratory cancer	38 (0.37)	211(0.54)
Gastrointestinal cancer	45 (0.44)	260 (0.66)
Liver and biliopancreatic cancer	14 (0.13)	57 (0.14)
Connective tissue cancer	2 (0.02)	12 (0.03)
Non melanoma skin cancer	115 (1.14)	586 (1.5)
Melanoma	11 (0.11)	67 (0.17)
Urinary tract cancer	22 (0.22)	90 (0.23)
Breast cancer	132 (1.83)	510 (1.83)
Gynecologic cancer	31 (0.43)	124 (0.44)
Male genital cancer	2 (0.07)	10 (0.09)
Prostate cancer	26 (0.9)	153 (1.37)
