# Supplementary material for: Increasing cancer risk over calendar year in people with multiple sclerosis: a case–control study
Source: J Neurol. 2020 Oct 21;268(3):817–24. doi: 10.1007/s00415-020-10170-5 (PMC7914231; doi:10.1007/s00415-020-10170-5)
Supplement: Supplementary file 3 — Supplementary file3 (PDF 13 kb) [file 415_2020_10170_MOESM3_ESM.pdf]

Supplementary Table 3 Multivariable Cox regression models testing the association of MS status, age at index date, gender and calendar year at index date with risk of specific types of cancer

Cancer type	Predicting variables	HR	95% CI	p
Hodgkin lymphoma	MS status (MS)	0.64	0.15-2.86	0.563
	Gender (F)	0.58	0.21-1.63	0.300
	Age at index date	1.01	0.97-1.05	0.662
	Calendar year at index date	1.12	0.99-1.26	0.079
Non-Hodgkin lymphoma	MS status (MS)	1.03	0.59-1.81	0.911
	Gender (F)	0.66	0.42-1.04	0.072
	Age at index date	1.07	1.05-1.09	**<0.001**
	Calendar year at index date	1.04	0.99-1.09	0.116
Leukaemia	MS status (MS)	0.69	0.37-1.30	0.251
	Gender (F)	0.79	0.50-1.25	0.320
	Age at index date	1.08	1.06-1.10	**<0.001**
	Calendar year at index date	1.03	0.98-1.08	0.240
Brain cancer	MS status (MS)	1.22	0.40-3.72	0.722
	Gender (F)	1.02	0.36-2.86	0.972
	Age at index date	1.03	0.99-1.07	0.165
	Calendar year at index date	1.07	0.96-1.19	0.202
Eye cancer	MS status (MS)	0.00	0.00-Inf	0.999
	Gender (F)	0.39	0.03-6.28	0.508
	Age at index date	1.02	0.91-1.15	0.693
	Calendar year at index date	1.09	0.76-1.55	0.646
Ear-nose-throat cancer	MS status (MS)	0.71	0.28-1.82	0.474
	Gender (F)	0.29	0.15-0.56	**<0.001**
	Age at index date	1.05	1.02-1.08	**<0.001**
	Calendar year at index date	1.00	0.93-1.07	0.993
Lung and respiratory	MS status (MS)	0.84	0.56-1.19	0.328
	Gender (F)	0.61	0.47-0.78	**<0.001**
	Age at index date	1.08	1.07-1.09	**<0.001**
	Calendar year at index date	0.99	0.96-1.01	0.348
Gastrointestinal cancer	MS status (MS)	0.80	0.59-1.10	0.175
	Gender (F)	0.52	0.41-0.65	**<0.001**
	Age at index date	1.08	1.07-1.09	**<0.001**
	Calendar year at index date	0.99	0.97-1.02	0.457
Liver and biliopancreatic cancer	MS status (MS)	1.12	0.62-2.01	0.711
	Gender (F)	1.10	0.65-1.86	0.729
	Age at index date	1.07	1.05-1.09	**<0.001**
	Calendar year at index date	1.07	1.01-1.13	0.017
Connective tissue cancer	MS status (MS)	0.76	0.17-3.42	0.723
	Gender (F)	0.99	0.31-3.16	0.986

Cancer type	Predicting variables	HR	95% CI	p
Non melanoma skin cancer	Age at index date	1.05	1.00-1.10	**0.033**
	Calendar year at index date	0.97	0.86-1.09	0.564
	MS status (MS)	0.89	0.73-1.09	0.251
	Gender (F)	0.76	0.65-0.88	**<0.001**
Melanoma	Age at index date	1.07	1.06-1.08	**<0.001**
	Calendar year at index date	1.00	0.99-1.02	0.891
	MS status (MS)	0.72	0.38-1.36	0.305
	Gender (F)	1.06	0.64-1.76	0.815
Urinary tract cancer	Age at index date	1.03	1.01-1.05	**0.003**
	Calendar year at index date	1.09	1.03-1.15	**0.002**
	MS status (MS)	1.14	0.72-1.82	0.580
	Gender (F)	0.33	0.23-0.48	**<0.001**
Breast cancer	Age at index date	1.08	1.06-1.09	**<0.001**
	Calendar year at index date	1.00	0.96-1.04	0.882
	MS status (MS)	1.12	0.93-1.36	0.234
	Gender (F)	..	..	..
Gynecologic cancer	Age at index date	1.03	1.03-1.04	**<0.001**
	Calendar year at index date	1.01	1.00-1.03	0.108
	MS status (MS)	1.08	0.73-1.60	0.701
	Gender (F)	..	..	..
Male genital cancer	Age at index date	1.03	1.02-1.05	**<0.001**
	Calendar year at index date	1.01	0.98-1.05	0.458
	MS status (MS)	0.82	0.18-3.75	0.800
	Gender (F)	..	..	..
Prostate	Age at index date	1.01	0.96-1.05	0.791
	Calendar year at index date	1.11	0.98-1.26	0.102
	MS status (MS)	0.81	0.53-1.22	0.311
	Gender (F)	..	..	..
	Age at index date	1.10	1.09-1.12	**<0.001**
	Calendar year at index date	1.02	0.99-1.06	0.166
